# Supplementary material for: Ultra-deep long-read metagenomics captures diverse taxonomic and biosynthetic potential of soil microbes
Source: Gigascience. 2025 Oct 24;14:giaf135. doi: 10.1093/gigascience/giaf135 (PMC12690461; doi:10.1093/gigascience/giaf135)
Supplement: giaf135_Supplemental_Files [file giaf135_supplemental_files.zip › Supplementary Figures.pdf]

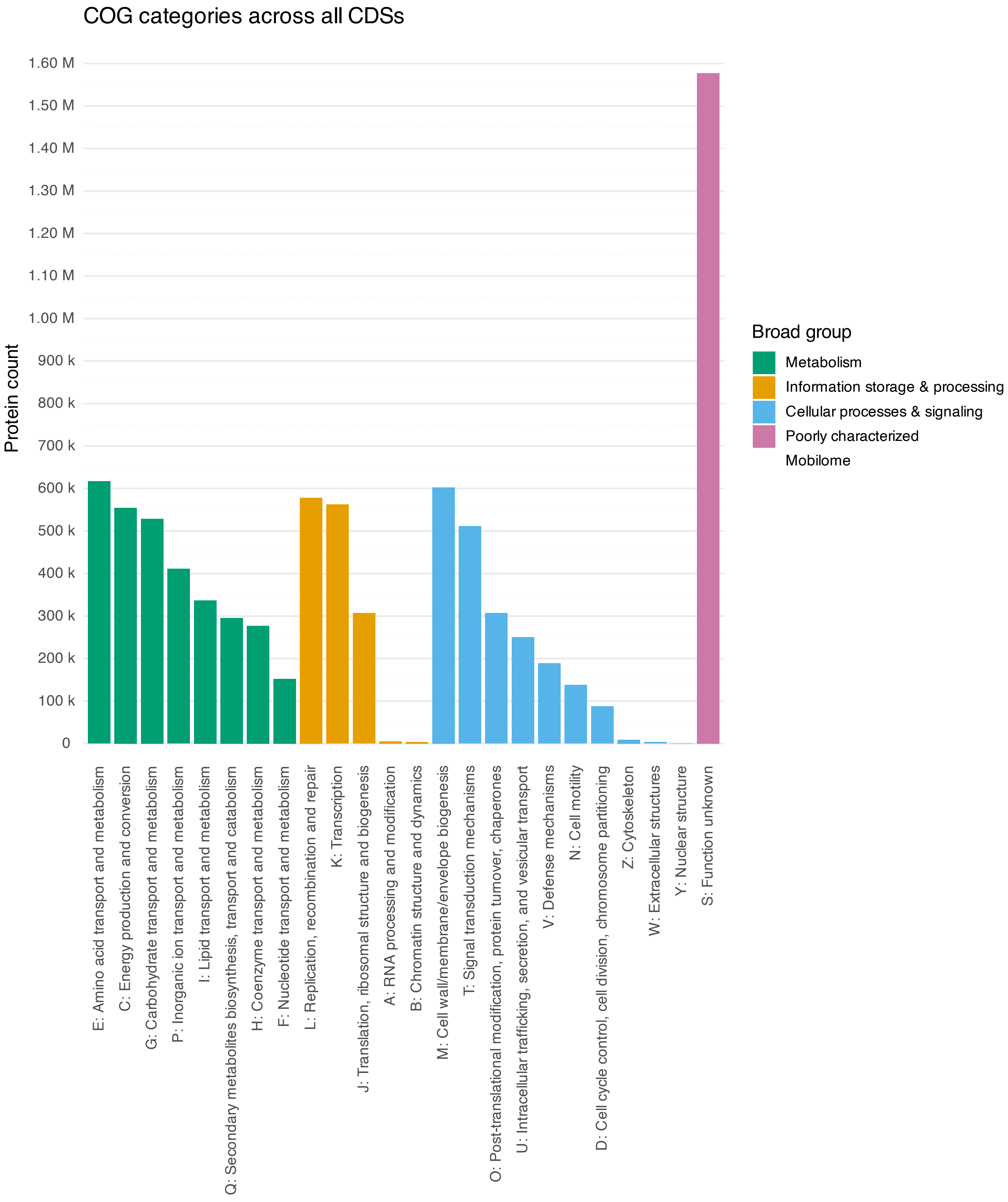

Supplementary Figure 1: Distribution of predicted proteins into COG categories.

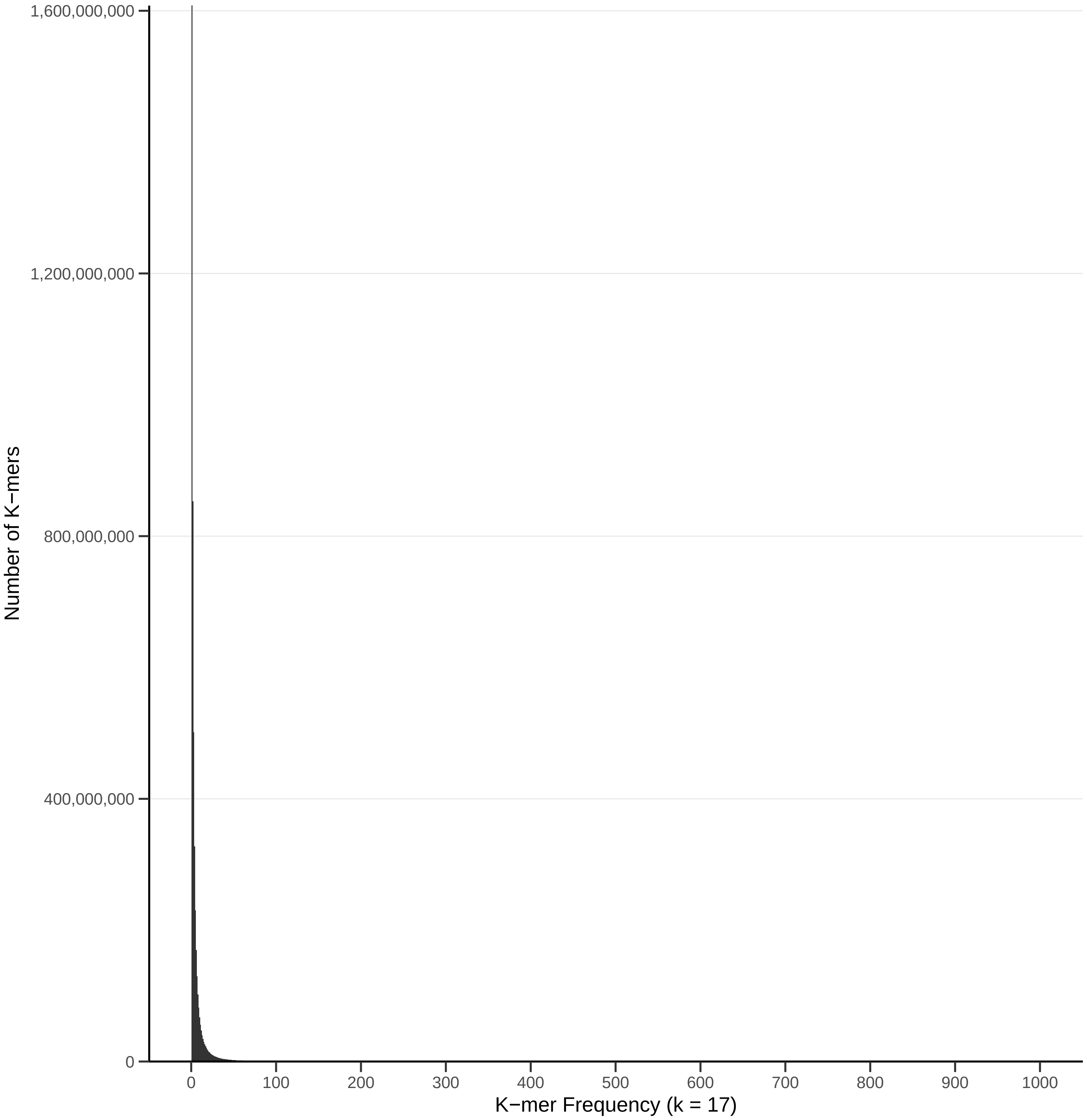

Supplementary Figure 2: 17-mer spectrum of Illumina reads.

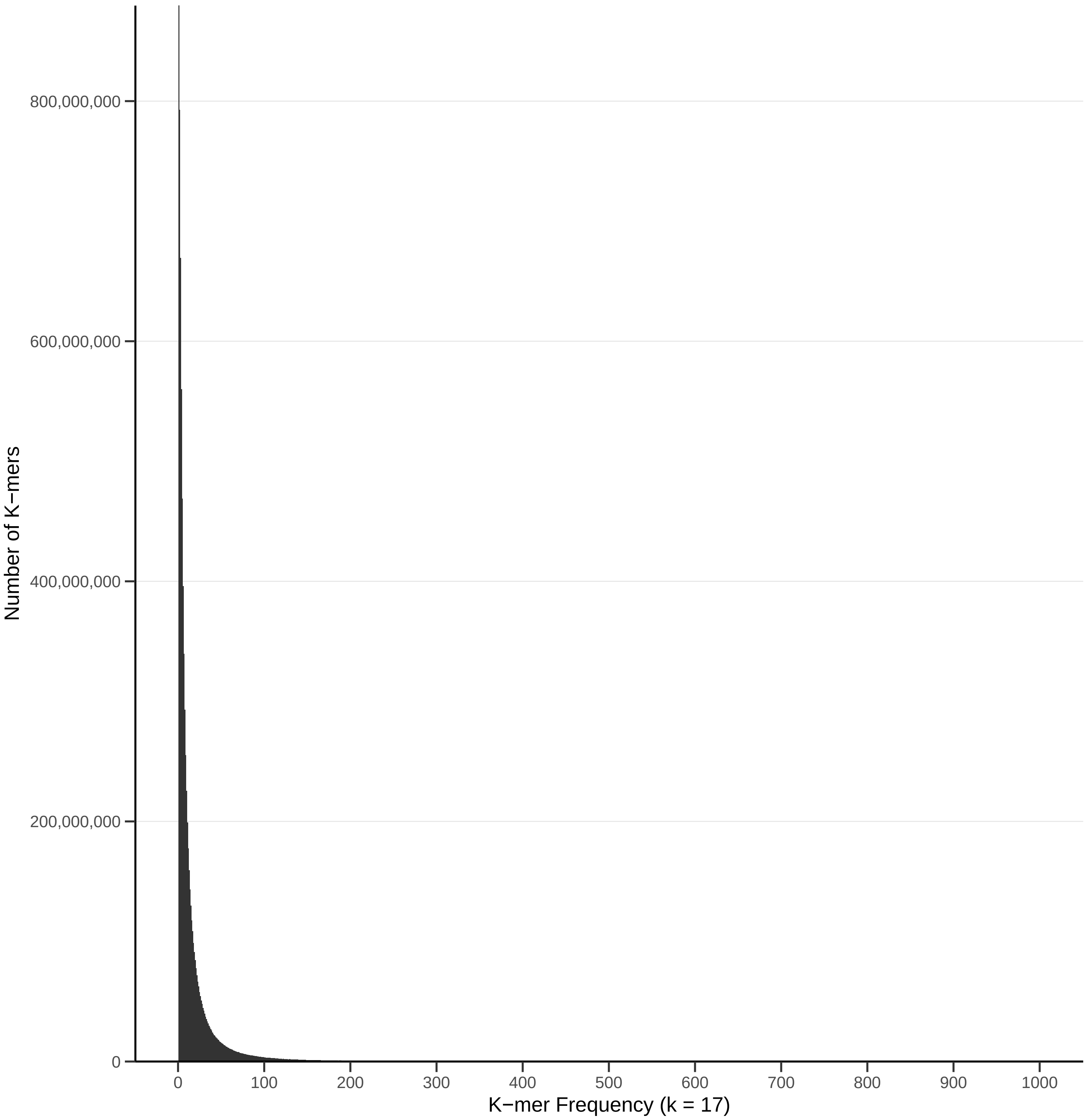

Supplementary Figure 3: 17-mer spectrum of Nanopore reads.

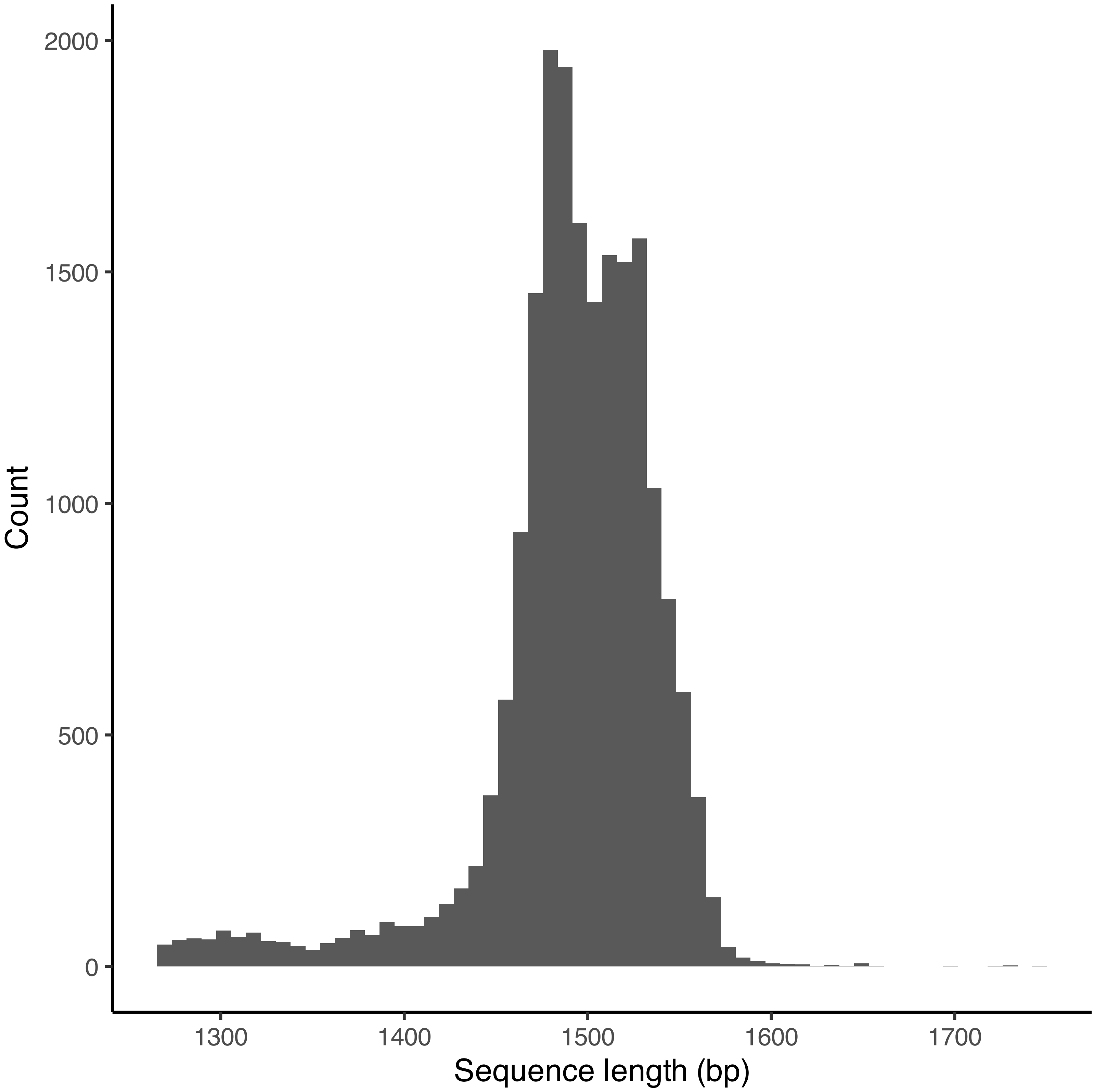

Supplementary Figure 4: Length distribution of the 16S rRNA gene sequences extracted from raw Nanopore reads.

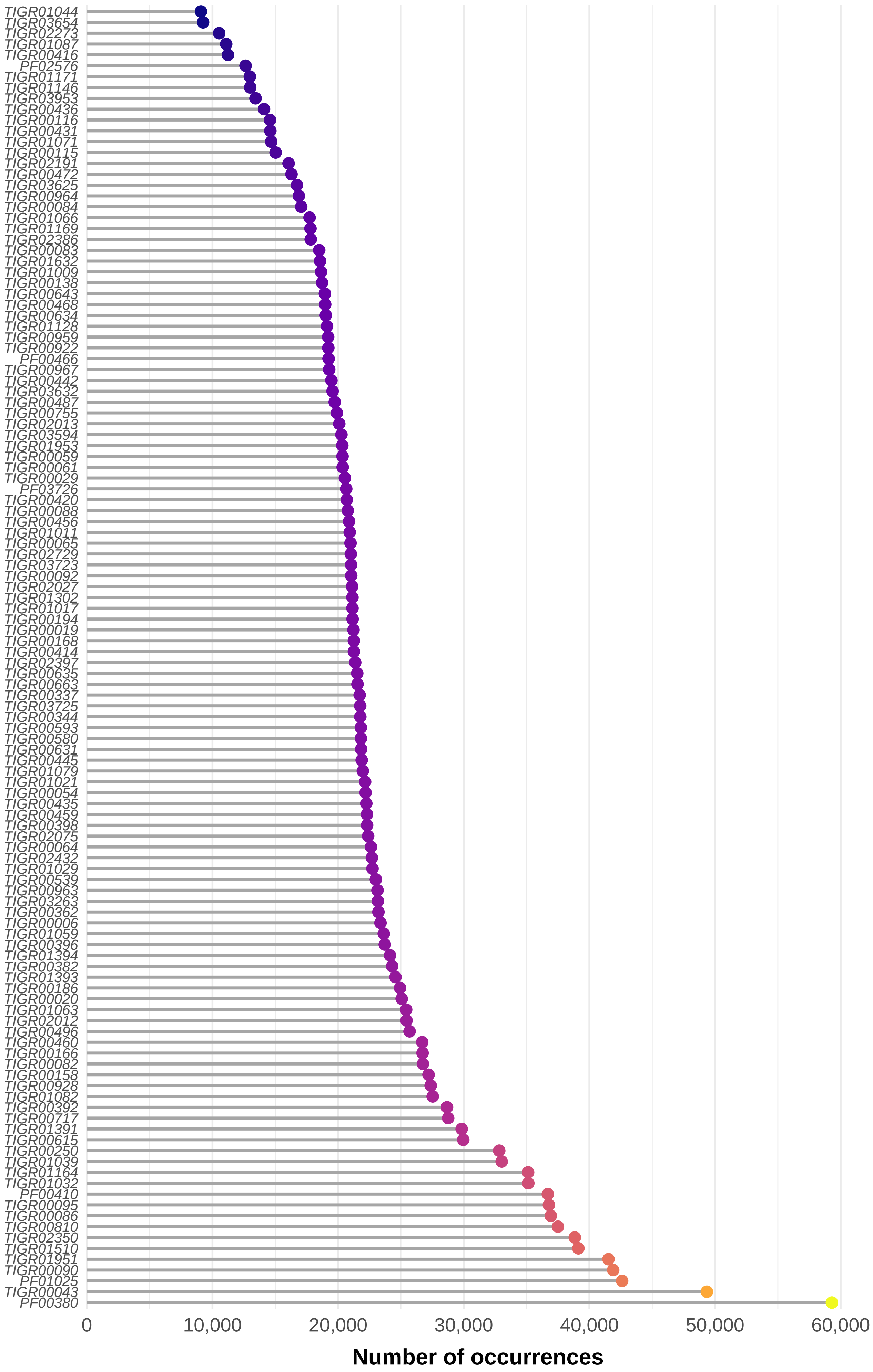

Supplementary Figure 5: The number of occurrences of each marker gene from the bac120 set in raw Nanopore reads.
