# Supplementary material for: Ultra-deep long-read metagenomics captures diverse taxonomic and biosynthetic potential of soil microbes
Source: Gigascience. 2025 Oct 24;14:giaf135. doi: 10.1093/gigascience/giaf135 (PMC12690461; doi:10.1093/gigascience/giaf135)
Supplement: giaf135_GIGA-D-25-00264_Original_Submission [file giaf135_giga-d-25-00264_original_submission.pdf]

# GigaScience

# Ultra-deep long-read metagenomics captures diverse taxonomic and biosynthetic potential of soil microbes

--Manuscript Draft--

| | |
|---|---|
| **Manuscript Number:** | GIGA-D-25-00264 |
| **Full Title:** | Ultra-deep long-read metagenomics captures diverse taxonomic and biosynthetic potential of soil microbes |
| **Article Type:** | Research |
| **Funding Information:** | Bundesministerium für Bildung und Forschung (161L0284C) — Dr. Nadine Ziemert<br>Deutsches Zentrum für Infektionsforschung (TTU09.716) — Dr. Nadine Ziemert<br>Deutsche Forschungsgemeinschaft (INST 37/1049-1) — Dr. Stephan Ossowski |
| **Abstract:** | Background: Soil ecosystems have long been recognised as hotspots of microbial diversity, but most estimates of their microbial and functional complexity remain speculative despite decades of study, in part because conventional sequencing campaigns lack the depth and contiguity required to recover low-abundance and repetitive genomes. Here, we revisit this question using one of the deepest metagenomic sequencing efforts to date, applying 148 billion base pairs of Nanopore long-read and 122 billion base pairs of Illumina short-read data to a single forest soil sample.<br>Results: Our hybrid assembly reconstructed 837 metagenome-assembled genomes, including 466 that meet high- and medium-quality standards, nearly all lacking close relatives among cultivated taxa. Rarefaction and k-mer analyses reveal that, even at this depth, we capture only a fraction of the extant diversity: non-parametric models project that more than ten trillion base pairs of sequencing data would be required to approach saturation. These findings offer a quantitative, technology-enabled update to long-standing diversity estimates and demonstrate that conventional metagenomic sequencing efforts likely miss the majority of microbial and biosynthetic potential in soil. We further identify more than 11 000 biosynthetic gene clusters, over 99% of which have no match in current databases, underscoring the breadth of unexplored metabolic capacity.<br>Conclusions: Taken together, our results emphasise both the power and the present limitations of metagenomics in resolving natural microbial complexity, and they provide a new baseline for evaluating future advances in microbial genome recovery, taxonomic classification, and natural product discovery. |
| **Corresponding Author:** | Nadine Ziemert<br>Universitätsklinikum Tübingen: Universitatsklinikum Tubingen<br>Tuebingen, GERMANY |
| **Corresponding Author Secondary Information:** | |
| **Corresponding Author's Institution:** | Universitätsklinikum Tübingen: Universitatsklinikum Tubingen |
| **Corresponding Author's Secondary Institution:** | |
| **First Author:** | Caner Bağcı, Dr. |
| **First Author Secondary Information:** | |
| **Order of Authors:** | Caner Bağcı, Dr.<br>Timo Negri, Dr.<br>Elena Buena Atienza, Dr.<br>Caspar Gross, MSc. |

Powered by Editorial Manager® and ProduXion Manager® from Aries Systems Corporation

| | |
|---|---|
| | Stephan Ossowski, Prof. Dr. |
| | Nadine Ziemert, Prof. Dr. |
| **Order of Authors Secondary Information:** | |
| **Additional Information:** | |
| **Question** | **Response** |
| Are you submitting this manuscript to a special series or article collection? | No |
| **Experimental design and statistics**<br><br>Full details of the experimental design and statistical methods used should be given in the Methods section, as detailed in our Minimum Standards Reporting Checklist. Information essential to interpreting the data presented should be made available in the figure legends.<br><br>Have you included all the information requested in your manuscript? | Yes |
| **Resources**<br><br>A description of all resources used, including antibodies, cell lines, animals and software tools, with enough information to allow them to be uniquely identified, should be included in the Methods section. Authors are strongly encouraged to cite Research Resource Identifiers (RRIDs) for antibodies, model organisms and tools, where possible.<br><br>Have you included the information requested as detailed in our Minimum Standards Reporting Checklist? | Yes |
| **Availability of data and materials**<br><br>All datasets and code on which the conclusions of the paper rely must be either included in your submission or deposited in publicly available repositories (where available and ethically appropriate), referencing such data using | Yes |

Powered by Editorial Manager® and ProduXion Manager® from Aries Systems Corporation

| | |
|---|---|
| a unique identifier in the references and in the “Availability of Data and Materials” section of your manuscript.<br><br>Have you have met the above requirement as detailed in our Minimum Standards Reporting Checklist? | |
| GigaScience has policies and guidelines in place for the use of generative AI-writing tools such as ChatGPT. If you have used such writing tools to assist with writing the manuscript this must be declared and cited in the text. Authors should not list AI-writing tools and other AI-assisted technologies as an author or co-author and should acknowledge that they are fully responsible for text generated or refined by AI-writing tools.<p><br><br>A summary of use (particularly in the introduction or among methods) needs to be included at the end of the paper, and the outputs should also be included as a supplementary file hosted in GigaDB or other open repositories. Please <a href=https://academic.oup.com/gigascience/pages/editorial_policies_and_reporting_standards target="_new" > read our guidelines for more information. </a> <p><br><br>By submitting to GigaScience, you are aware of the journal's AI-writing tools policy, and if you have declared use of such tools below, you have acknowledged this where appropriate in your manuscript and have made a summary of use and outputs available. </b><p><br><b>AI-assisted writing tools have been used in the preparation of this manuscript? | No |

LaTeX - Main Document (TeX file)
Click here to access/download;LaTeX - Main Document (TeX file);main.tex

Placeholder for OUP logo oup.pdf

Placeholder for journal logo gigascience-logo.pdf

*GigaScience*, 2025, 1–12

doi: xx.xxxx/xxxx
**Manuscript in Preparation**
**Research**

RESEARCH

# Ultra-deep long-read metagenomics captures diverse taxonomic and biosynthetic potential of soil microbes

Caner Bağcı[1,2,3], Timo Negri[1,2,3], Elena Buena Atienza[4,5], Caspar Gross[4,5], Stephan Ossowski[4,5] and Nadine Ziemert[1,2,3,*]

[1]Translational Genome Mining for Natural Products, Interfaculty Institute of Microbiology and Infection Medicine Tübingen (IMIT), University of Tübingen, Auf der Morgenstelle 24, 72076, Tübingen, Baden Württemberg, Germany and [2]Interfaculty Institute for Biomedical Informatics (IBMI), University of Tübingen, Auf der Morgenstelle 24, 72076, Tübingen, Baden Württemberg, Germany and [3]German Center for Infection Research (DZIF), Partner Site Tübingen, Auf der Morgenstelle 24, 72076, Tübingen, Baden Württemberg, Germany and [4]Institute of Medical Genetics and Applied Genomics, University of Tübingen, Calwerstrasse 7, 72076, Tübingen, Baden Württemberg, Germany and [5]NGS Competence Center, University of Tübingen, Calwerstrasse 7, 72076, Tübingen, Baden Württemberg, Germany

[*]nadine.ziemert@uni-tuebingen.de

## Abstract

**Background:** Soil ecosystems have long been recognised as hotspots of microbial diversity, but most estimates of their microbial and functional complexity remain speculative despite decades of study, in part because conventional sequencing campaigns lack the depth and contiguity required to recover low-abundance and repetitive genomes. Here, we revisit this question using one of the deepest metagenomic sequencing efforts to date, applying 148 billion base pairs of Nanopore long-read and 122 billion base pairs of Illumina short-read data to a single forest soil sample.
**Results:** Our hybrid assembly reconstructed 837 metagenome-assembled genomes, including 466 that meet high- and medium-quality standards, nearly all lacking close relatives among cultivated taxa. Rarefaction and k-mer analyses reveal that, even at this depth, we capture only a fraction of the extant diversity: non-parametric models project that more than ten trillion base pairs of sequencing data would be required to approach saturation. These findings offer a quantitative, technology-enabled update to long-standing diversity estimates and demonstrate that conventional metagenomic sequencing efforts likely miss the majority of microbial and biosynthetic potential in soil. We further identify more than 11 000 biosynthetic gene clusters, over 99% of which have no match in current databases, underscoring the breadth of unexplored metabolic capacity.
**Conclusions:** Taken together, our results emphasise both the power and the present limitations of metagenomics in resolving natural microbial complexity, and they provide a new baseline for evaluating future advances in microbial genome recovery, taxonomic classification, and natural product discovery.

**Key words**: microbiome; soil; metagenomics; nanopore; ultra-deep; long-read sequencing; diversity; natural products; (meta)genome mining

## Data Description

In order to quantify how much taxonomic and biosynthetic novelty present-day sequencing can recover from soil, we extracted high-molecular-weight DNA from a single Cambisol forest soil sample (Schönbuch, Germany) and generated an ultra-deep 270 Gbp dataset — 148 Gbp of Oxford Nanopore PromethION long reads (read-length N50 = 12.2 kb) plus 122 Gbp of Illumina NovaSeq

**Compiled on:** July 3, 2025.
Draft manuscript prepared by the author.

**Key Points**

- Ultra-deep hybrid sequencing (148 Gbp Nanopore + 122 Gbp Illumina) of a single forest soil sample yielded 837 metagenome-assembled genomes, all lacking cultured counterparts.
- Despite this unprecedented 270 Gbp depth, rarefaction and coverage modelling indicate that more than 10 Tbp of data would still be needed to reach saturation in soil.
- Only 0.7% and 16.7% of all assembled contigs can be assigned to a species and genus, respectively, with at least one cultured representative, highlighting an unprecedented level of novelty in soil.
- The assembly uncovers 11 381 biosynthetic gene clusters forming over 10 000 mostly novel families, spotlighting an immense, untapped reservoir of microbial natural-product potential.

reads. The hybrid metagenome assembly we conducted produced a 10.5 Gbp assembly, from which multi-tool binning/refinement recovered 837 MAGs, and antiSMASH/BiG-SCAPE annotation revealed >11 000 largely novel biosynthetic gene clusters, creating a resource for benchmarking assembly or binning pipelines, modelling diversity-coverage relationships and mining natural products; all raw reads are archived under ENA BioProject PRJEB89893 and the polished assembly, MAG set and BGC catalogue are available via Zenodo (https://zenodo.org/records/15533781; doi.org/10.5281/zenodo.15529477) for unrestricted reuse.

## Introduction

Soil is one of the most biologically diverse ecosystems on Earth, hosting a massive and largely uncharacterised diversity of microbial life. These microbial communities play critical roles in biogeochemical cycling, soil formation, nutrient turnover, plant health, and climate regulation [1]. Despite their ecological and biotechnological importance, the full extent and functional potential of soil microbiomes remain poorly understood.

A single gram of soil can contain up to $10^9$ microbial cells and hundreds of thousands of species, spanning all domains of life [2, 3]. These communities are not only taxonomically complex but also exhibit highly uneven abundance distributions, where rare taxa – collectively termed the "rare biosphere" – may be functionally consequential despite their low abundance [4].

Although the vast diversity of soil microbes has been widely acknowledged for decades, most estimates of their richness are based on indirect approaches, including 16S rRNA gene surveys, limited shotgun datasets, or predictive modelling. As a result, foundational claims about the extent of soil microbial diversity are frequently cited but rarely re-examined with contemporary data. Much of the soil microbiome remains part of the so-called microbial dark matter: lineages with no cultured representatives and no reference genomes [5, 6, 7].

Metagenomics has enabled the cultivation-independent study of microbial communities, allowing for genome-resolved insights into uncultured organisms [8, 9, 10]. However, most metagenomic studies rely on short-read sequencing, which presents challenges in assembling highly complex, strain-rich communities like soil. These limitations hinder the recovery of low-abundance taxa, reduce the completeness of biosynthetic gene clusters, and often lead to fragmented or ambiguous assemblies [9]. Recent advances in long-read sequencing, particularly with Oxford Nanopore Technologies (ONT), have opened new opportunities for soil microbiome research. Long reads can span repeat-rich regions and operons, improving genome recovery and assembly contiguity [11, 12, 13]. Hybrid approaches combining long and short reads can further mitigate the high error rates of long reads while leveraging their structural advantages [14, 15]. Despite these improvements, it remains unclear how far even the most advanced sequencing technologies can go in capturing the full taxonomic and functional diversity of soil.

To establish an empirical reference point for assessing microbial and biosynthetic diversity in soil, we performed one of the deepest metagenomic sequencing efforts to date on a single soil sample, combining 148 billion base pairs (Gbp) of ONT long-read and 122 Gbp of Illumina short-read data. We use this dataset to empirically assess the power and current limitations of metagenomics in resolving complex microbial communities. Specifically, we ask: How much taxonomic and biosynthetic diversity can be recovered from a single soil sample using state-of-the-art sequencing? What proportion of this diversity is represented in existing databases? Can ultra-deep sequencing approach saturation, or are we still only scratching the surface? By addressing these questions, we aim to provide a data-driven reassessment of microbial diversity in soil and to establish a benchmark for future metagenomic studies of complex ecosystems.

## Methods

### Sample collection, DNA extraction, and metagenomic sequencing

The A horizon of the soil type Cambisol was sampled from the Schönbuch Forest, near Tübingen, Germany (the same site previously reported by [16]), on 31 May 2022. High molecular weight metagenomic DNA was isolated using a protocol described in detail in our previous studies [16, 17]. Genomic integrity was assessed using pulse-field capillary electrophoresis with the Genomic DNA 165 kb Analysis Kit on a FemtoPulse (Agilent) instrument. Quantitation of DNA was assessed using the dsDNA High Sensitivity Assay on a Qubit 3 fluorometer (Thermo Fisher), and purity was assessed by Nanodrop. A total of 2.4 µg of genomic DNA was used as input for the library preparation with the 1D Ligation Kit SQK-LSK109-XL Sequencing Kit (ONT). Four PromethION R9 flow cells were utilised for long-read Nanopore sequencing, each loaded with 600 ng (50 fmol) of genomic DNA. The raw signal data from the PromethION runs were basecalled with guppy (v 5.0.7) in high-accuracy mode. For complementary short-read sequencing, three NovaSeq 6000 flowcells were loaded with the same isolated DNA; two of them ran with 200 cycles, and one with 300 cycles.

A total of 148 Gbp of Nanopore sequencing data was generated, with a read length N50 of 12.2 kb. The three Illumina sequencing runs yielded a total of 122 Gbp raw data, with a mean Q-score of 35. The raw Illumina reads were adapter and quality trimmed using fastp [18] (v 0.23.4) with default settings.

### Metagenomic assembly

Nanopore reads from all four runs were pooled together and assembled using metaFlye (v2.9.5-b1801) [19] with the `-meta` option optimised for metagenomic data, and the `-nano-raw` option for the error-prone Nanopore reads. The resulting draft assem-

bly was polished with one round of medaka [20] (v 2.0.1) using the r941_prom_hac_g507 model and the -bacteria flag. The medaka polished assembly was further corrected using the trimmed Illumina reads in a single round of NextPolish [21] (v 1.4.1), with parameters -max_depth 100 for short-read mapping with bwa [22] (v 0.7.18), and -min_read_len 1k -max_depth 100 -x map-ont for long-read mapping minimap2 [23] (v 2.28-r1209).

In parallel, an Illumina-only assembly was performed using MEGAHIT [24] (v1.2.9) with default metagenomic assembly parameters. All subsequent analyses were based on the hybrid Nanopore-Illumina assembly, which exhibited superior contiguity (contig N50 = 77.8 kb) and total assembled length (10.5 Gbp).

## Taxonomic analysis

Raw reads were taxonomically classified using Metabuli [25] (v 1.0.8), against the precomputed database provided by the authors, which includes GTDB release 214.1 [26] and the human T2T genome [27]. Classification was performed with parameters -seq-mode 2 for Illumina reads, and -seq-mode 3 for Nanopore reads. Contigs from the final hybrid assembly were taxonomically classified using MMSeqs2 [28] (v 16.747c6) in easy-taxonomy mode, with GTDB release 214.1 [26].

## Reconstruction of metagenome-assembled genomes

Metagenome-assembled genomes(MAGs) were recovered using a combination of complementary binning strategies to maximise genome quality. Binning was first performed independently on the final NextPolish-corrected assembly using COMEBin [29], MetaDecoder [30], SemiBin2 [31], VAMB [32], executed in a CUDA-enabled environment when possible.

COMEBin (v 1.0.4), together with CheckM (v 1.1.3) [33], was run on mapping files generated by minimap2 [23], with the following parameters: NUM_VIEWS=6, TEMPERATURE=0.07, EMBEDDING_SIZE=2048, COVERAGE_EMBEDDING_SIZE=2048, and BATCH _SIZE=1024. MetaDecoder (v 1.1.0) was executed with default parameters according to the authors' recommendations. SemiBin2 (v 2.1.0) was run in both -self-supervised and -semi-supervised modes, specifying soil as the environment type and long_reads as the sequencing type. Gene prediction was performed using Prodigal (v 2.6.3) [34], and the GTDB v95 database [26] was used alongside the previously described minimap2 mappings of the long reads.

For VAMB (v 4.1.4) [32], six binning results were generated by combining default and taxonomy-aware binning modes with three input configurations: long reads, short reads, and both. Abundance profiles were computed using Strobealign (v 0.15.0) [35]. For TaxVAMB [36], contig-level taxonomic annotations were derived using Metabuli [25] against the GTDB reference database [26].

In order to produce a final, unified metagenome-assembled-genome binning, the binning results from the above-mentioned ten runs were collected, and refined using MAGScoT [37] (v 1.1). MAGScot was run on gene-calling results from prodigal [34] (v 2.6.3), and hmmsearch results from the HMMER package (v 2.6.3) [38], using domain profiles from PFAM [39] and TIGRFAMs [40] databases.

The resulting MAGs were evaluated for their completeness and contamination with CheckM2 [41] (v 1.0.2). Pairwise genomic distances were computed using Mash [42] (v 2.3) with default parameters. Taxonomic assignments were performed with GTDB-Tk [43] (v 2.4.0) using the GTDB r220 release [26] in classify mode. A phylogenetic tree of high- and medium-quality MAGs was constructed with GTDB-Tk in *de novo* mode and visualised with iTOL [44].

## Rarefaction analysis

Microbial diversity and sequencing coverage were assessed using multiple complementary approaches. K-mer frequency analysis was conducted with ntCard [45] using 15-mers and 17-mers for both Nanopore and Illumina datasets. Nonpareil [46] (v3.3.3) was used to estimate sequence coverage and to project additional sequencing efforts required for complete community recovery. Full-length 16S rRNA gene sequences were extracted from Nanopore reads using Barrnap [47] (v0.9), and clustered into operational taxonomic units (OTU) at various identity thresholds (100%, 97%, 95%, etc.) using VSEARCH [48] (v2.15.2). Rarefaction curves were constructed by subsampling reads and calculating cumulative OTU counts. In addition, conserved single-copy marker genes from the bac120 set [10] were identified in Nanopore reads using LAST aligner (v 1615) in frameshify mode (-F 15) against the GTDB bac120 reference set, and quantified to independently assess the taxonomic diversity.

## Annotation, detection, and clustering of biosynthetic gene clusters

The final assembly and refined MAGs were annotated using Bakta [49] (v 1.11.0, database version 5.1). Biosynthetic gene clusters (BGCs) were identified using antiSMASH [50] (v7) with the options -fullhmmer -clusterhmmer -tigrfam -asf -cc-mibig -cb-general -cb-subclusters -cb-knownclusters -pfam2go -rre -smcog-trees -tfb. Identified BGCs were subsequently clustered into gene cluster families (GCFs) using BiG-SCAPE [51] (v2 beta5), based on domain architecture and sequence similarity. The novelty of BGCs was assessed by comparisons against the MIBiG 4.0 [52] and BGC Atlas [53] databases.

# Results

## Ultra-deep hybrid sequencing of a temperate forest sample

To evaluate the limits of current sequencing technologies in capturing soil microbial diversity, we generated an ultra-deep metagenomic dataset from a single sample collected in the Schönbuch forest, a temperate mixed forest in southwestern Germany. The sample was taken from the A horizon of a Cambisol soil, a type of soil known for its rich microbial diversity and favourable characteristics for DNA extraction. This particular site and soil type were previously shown to harbour high biosynthetic potential and taxonomic richness [16], and were therefore selected as a representative model system for deep metagenomic exploration.

We extracted total environmental DNA from this soil and sequenced it using a hybrid strategy that combined 148 Gbp of ONT long-read and 122 Gbp of Illumina short-read sequencing. Nanopore reads had a read length N50 of 12.2 kb, while Illumina reads achieved a mean Q-score of 35. Together, this 270 Gbp dataset represents one of the deepest single-sample soil metagenomes reported to date, and provides a unique opportunity to empirically assess taxonomic and functional complexity.

## Hybrid assembly recovers hundreds of genomes with high taxonomic and biosynthetic novelty

We employed a hybrid assembly strategy to combine the complementary strengths of long and short reads. Long-read Nanopore data were assembled and subsequently polished using Illumina data, resulting in 10.5 Gbp of assembled sequence with a contig N50 of 77.8 kb. For comparison, an Illumina-only assembly produced a more fragmented assembly with an N50 of only 865 bp and a total length of 9.2 Gbp, confirming the advantages of long-read

inclusion.

From the hybrid assembly, we recovered 837 metagenome-assembled genomes (MAGs), of which 466 met the MIMAG high- or medium-quality criteria [54]. All MAGs recovered represented unique species-level units, defined by > 5% pairwise Mash distance. These genomes spanned a wide range of bacterial phyla typical of soils, including Acidobacteriota, Pseudomonadota, Verrucomicrobiota, Actinomycetota, and Myxococcota. Remarkably, only four of the 837 MAGs had species-level matches (>= 95% ANI) in the GTDB database, and none had cultured representatives.

Thirteen MAGs were assigned to phyla with no cultured members, such as Desulfobacterota, and candidate phyla radiation (CPR) groups FCPU426 and JAJYCY01, harbouring between 2 and 19 BGCs (Figure 1). At other taxonomic levels, 9, 45, 155, and 611 MAGs belong to novel lineages that correspond to class, order, family, and genus levels, respectively.

High- and medium-quality MAGs ranged in size from 1 Mbp (Patescibacteria) to 15 Mbp (Planctomycetota) (Figure 1 and Supplementary Table S1).

Many of the recovered MAGs are assigned to taxonomic groups that do not correspond to any known families or even classes in current reference databases (Figure 1). This points to a substantial fraction of microbial diversity in soil that remains undescribed, even at higher taxonomic ranks. In addition to their taxonomic novelty, many of these genomes contain a large number of BGCs, in some cases reaching 30 distinct clusters within a single genome. This suggests that the potential for secondary metabolite production is not restricted to well-characterised soil taxa (such as Actinomycetota), but is also widespread among uncharacterised lineages.

## Taxonomic analysis reveals unprecedented levels of novelty

Taxonomic classification of both the raw reads and the assembled contigs revealed a highly diverse and complex microbial community, comprising members from 69 different phyla. As expected for soil environments, the community was dominated by taxa such as Pseudomonodata and Acidobacteriota (Figure 2A), along with representatives from many other phyla. A small fraction (approximately 1%) of the reads originated from eukaryotes, while 8% remained unclassified, indicating the presence of deeply novel sequences absent from current databases.

Taxonomic analysis of the assembled contigs (Figure 2B) further demonstrated that a substantial portion of the dataset originates from uncultivated organisms across all taxonomic ranks. While soil has long been recognised as a reservoir of uncultivated diversity, with historical estimates suggesting 90–99% of taxa remain uncultured, our data now provide quantitative, genome-resolved confirmation of this. At the species level, more than 99% of the assembled contigs could not be assigned to any taxon with a cultured representative. Only 0.7% of contigs could be linked to species with at least one cultured strain, and 16.7% to a genus with cultured members.

Even at broader taxonomic levels, substantial novelty is evident: 9% of contigs could not be assigned to any known class, and 6.2% could not be linked to any known phylum (Figure 2B). Moreover, for those contigs that were classified, the majority were affiliated with taxa composed exclusively of uncultivated representatives, often known only from metagenomic or single-cell sequencing studies. These patterns are in contrast to metagenomic datasets from more tractable environments, such as the human gut or ocean microbiomes, where a larger fraction of genomes can be linked to cultivated taxa [55, 56].

Despite the expansion of public genome collections such as GTDB [26] and the GEM catalog [9], fewer than five of the 837 MAGs recovered in this study share at least 95% ANI with any known species. This confirms that soil remains one of the most genomically undercharacterised microbial ecosystems.

## Soil diversity remains undersampled

To evaluate whether ultra-deep sequencing could fully capture the microbial diversity present in the sample, we applied a combination of k-mer analysis, rarefaction based on marker genes and full-length 16S rRNA genes. All approaches consistently indicated that, despite the unprecedented depth of sequencing, the dataset remains far from saturating the underlying biological diversity.

K-mer analysis, which examines the frequency distribution of short subsequences within the sequencing reads, revealed a strong left-skewed distribution (Figure 3A). The majority of k-mers occurred only once or a few times, indicating a high prevalence of unique or rare sequences. This pattern is characteristic of highly diverse communities and reflects the presence of many low-abundance taxa whose genomes were either incompletely covered or missed entirely in assembly. A long right-hand tail, representing high-frequency k-mers, likely originates from abundant taxa or repetitive genomic elements (such as 16S rRNA genes). These patterns were observed both in Nanopore and Illumina datasets, and are consistent with extreme sequence heterogeneity of the sample (Figure 3A, and Supplementary Figures 1 and 2).

Rarefaction analysis using Nonpareil further supported this conclusion. By estimating the redundancy of reads, the model projected that approximately 10 terabases of sequencing data would be necessary to achieve near-complete coverage of the microbial diversity in this single soil sample — a nearly 50-fold increase over the current deep-sequencing effort (Figure 3B). This analysis confirms that even the most comprehensive sequencing efforts to date fall short of fully resolving the taxonomic complexity of soil microbiomes.

In parallel, we extracted full-length 16S rRNA gene sequences from Nanopore reads and clustered them into operational taxonomic units (OTUs) at various sequence identity thresholds. At 100% and 97% identity, the number of observed OTUs continued to increase linearly with sequencing depth, showing no indication of saturation (Figure 4A). While higher-order groupings (e.g., family or phylum) began to plateau, even genus-level rarefaction curves remained unsaturated. A complementary analysis based on 120 conserved single-copy bacterial marker genes showed similar trends. For each marker gene, we detected between 9000 and 59 000 unique copies across the dataset (Supplementary Figure 3).

Taken together, these results provide robust and multi-dimensional evidence that the soil microbial community remains vastly undersampled, even with sequencing efforts far beyond typical practice. The failure to reach saturation was especially apparent at the species level, where both OTU-based and marker gene rarefaction curves continued to rise linearly across all subsampling depths. This indicates an exceptional degree of fine-scale taxonomic resolution, with many closely related but genomically distinct lineages present at low abundance.

Such strain- and species-level diversity is particularly important given that many ecologically and functionally relevant traits – such as niche specialisation, symbiosis, and secondary metabolite biosynthesis – can differ even between closely related strains. In contrast to more constrained environments such as the human gut, where species-level diversity tends to plateau with a few hundred genomes [57], or marine planktonic communities, which show relatively stable core microbiomes [58], the soil microbiome appears to exhibit near-limitless diversity at the genomic level.

These findings not only demonstrate the limitations of current sequencing depths but also call into question the accuracy of historical species richness estimates derived from much shallower data. They suggest that achieving anything approaching species-level saturation in soil will likely require terabase- to petabase-scale sequencing efforts, combined with refined computational methods to resolve low-abundance and highly diverse taxa. They demon-

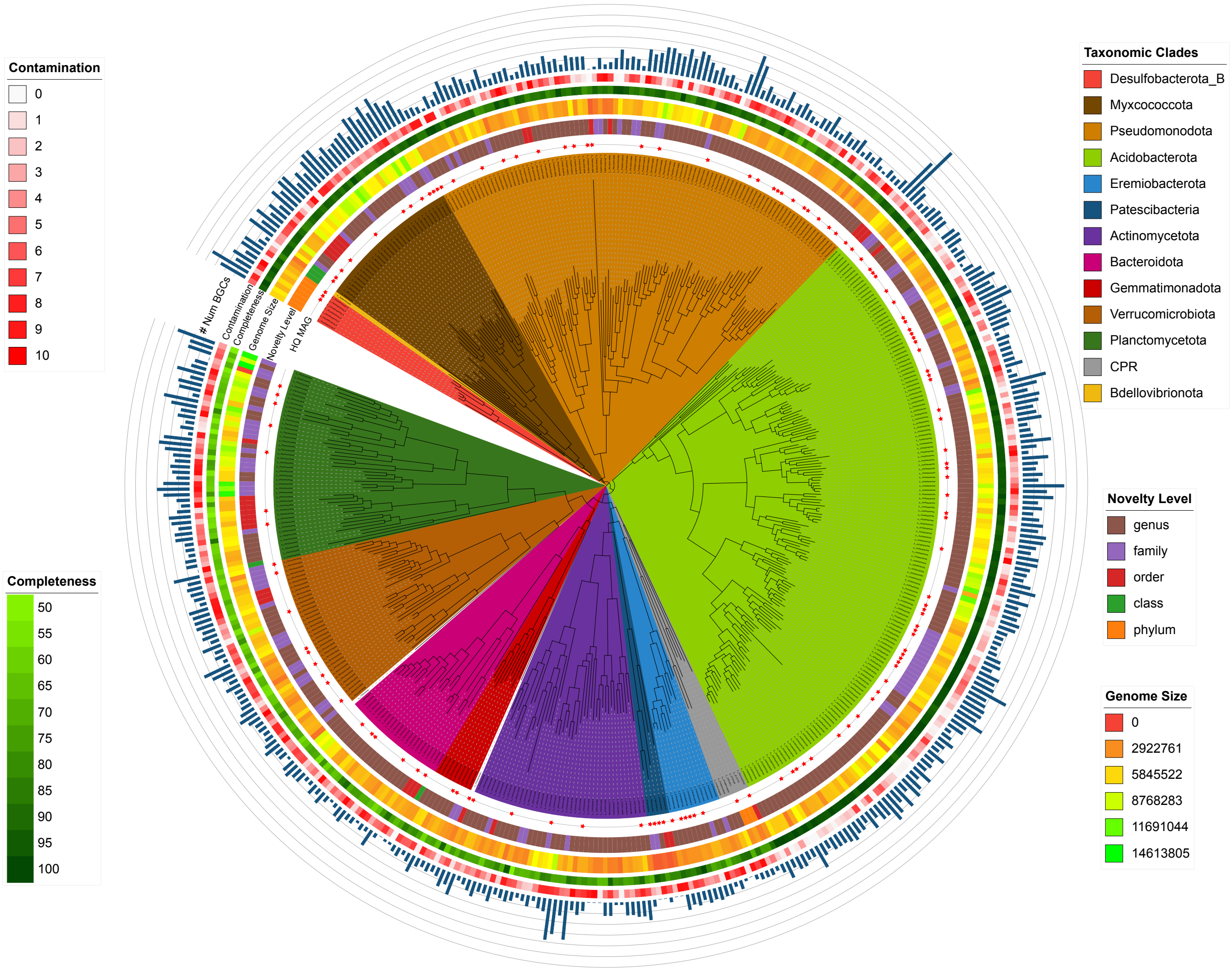


**Figure 1.** Genome quality statistics and taxonomic classification of newly reconstructed high-quality and medium-quality metagenome-assembled genomes (MAGs). Each leaf represents a MAG, coloured by its assigned phylum. Concentric rings around the leaves indicate, from innermost to outermost: (i) HQ MAGs, (ii) novelty level (genus to phylum), (iii) genome size (bp), (iv) completeness (%), (v) contamination (%), and (vi) the number of BGCs encoded by the MAG (range: 0-30).

strate that many of the diversity estimates historically used in soil microbiome research, often based on much shallower sequencing, likely underestimate the true scale of taxonomic complexity.

## Long-read metagenomics recovers thousands of novel and genome-linked BGCs

From the hybrid metagenomic assembly, we identified a total of 11 381 BGCs, including 5652 classified as complete, reflecting the improved contiguity made possible through long-read sequencing. This proportion of complete clusters (~50%) represents a substantial improvement over previous metagenomic surveys, where fewer than 10% of BGCs are typically annotated as complete [53].

Clustering the identified BGCs with BiG-SCAPE resulted in 10 215 gene cluster families, indicating that most BGCs in our dataset are non-redundant and functionally distinct. The distribution of GCF sizes (Figure 5A) was strongly skewed: most families consisted of singletons or doubletons, with only a handful of larger families, further illustrating the functional uniqueness and fine-grained metabolic specialisation within the community. The largest GCF contains 115 ribosomally synthesised and post-transcriptionally modified peptide (RiPP) BGCs. The cumulative ordering of the GCFs by size shows that the largest eight families account for 3.1% of all BGCs, whereas each of the remaining over ten thousand families contributes only very little to the total number (Figure 5B).

Taxonomic classification of the BGC-containing contigs revealed that biosynthetic potential is broadly distributed across the phylogenetic spectrum. While well-known producers such as Actinomycetota and Pseudomonadata were represented, we also detected numerous BGCs in less-studied phyla such as Verrucomicrobiota and Acidobacteriota (Figure 5C). These groups have historically been overlooked due to cultivation barriers but appear to harbour substantial secondary metabolic capacity.

Only 108 BGCs (~ 1%) had significant matches to known GCFs in the BGC Atlas database, which compiles nearly two million BGCs from metagenomic sources. This confirms earlier observations that soil metagenomes are rich in biosynthetic novelty [12, 51], but also demonstrates that even with massive reference expansions, a typical soil still contains an overwhelming majority of uncharacterised BGCs. Notably, our dataset contains more novel BGCs than reported in most large-scale studies of marine or human microbiomes, where the rate of novel BGC discovery is often constrained by lower microbial diversity and shorter contigs [51, 53].

Of all BGCs identified, 6279 could be linked to metagenome-assembled genomes, enabling genome-resolved exploration of natural product potential. This represents one of the largest genome-resolved inventories of BGCs from a single environmental sample to date.

Together, these results point to a biosynthetic landscape in soil that is both expansive and largely underexplored. The ability to

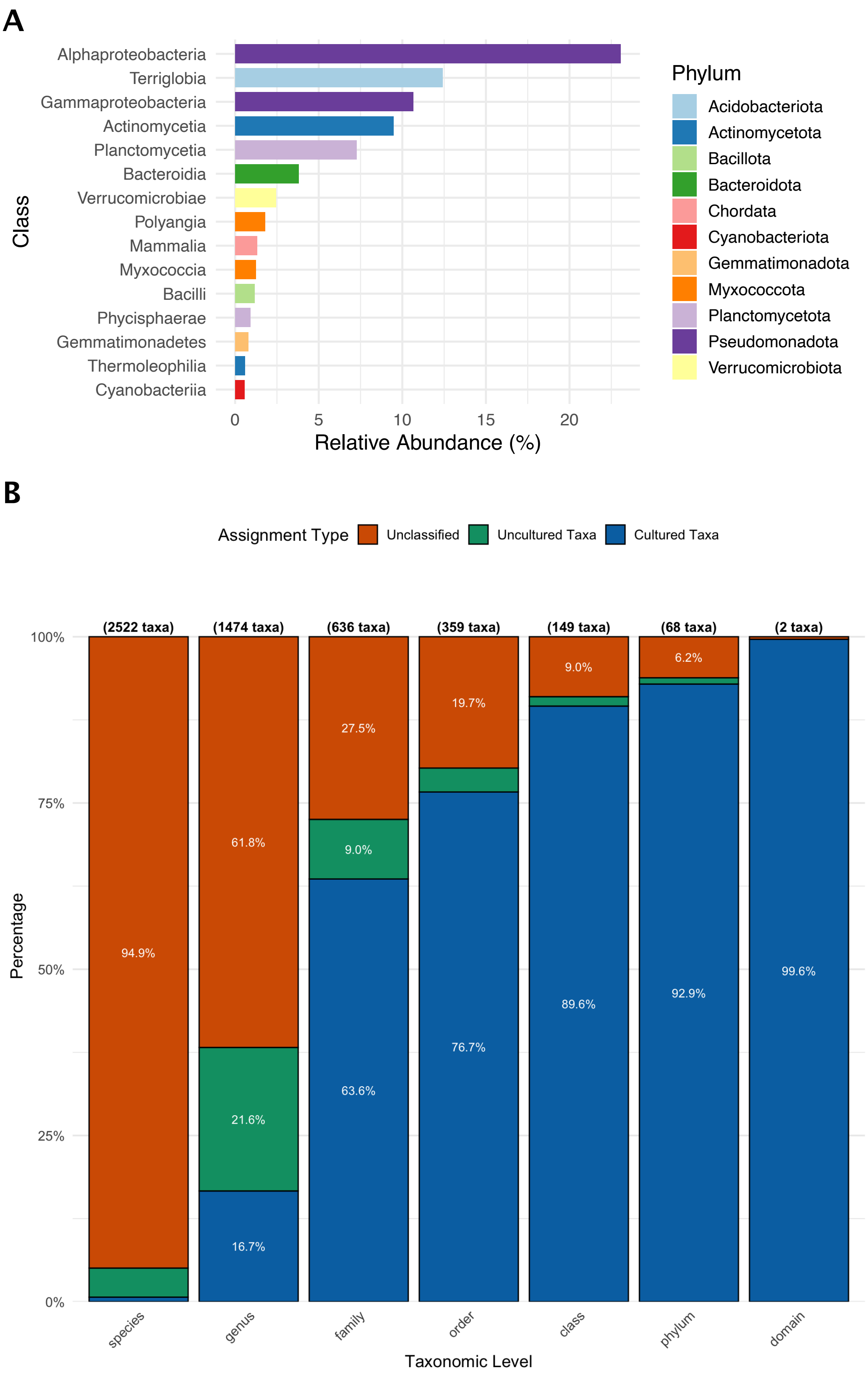


**Figure 2.** Taxonomic summary of the assembled contigs. **(A)** Class-level relative abundances of the assembled contigs, coloured by the phyla they belong to. **(B)** Taxonomic novelty levels of the assembled contigs across different ranks. Bars represent the proportion of contigs assigned to cultured taxa (blue), uncultured taxa (only observed in other metagenome studies) (green), or remaining unclassified (completely novel) (orange) at each taxonomic level, from species to domain. The number of taxa identified at each level is shown above each bar. Most contigs could only be classified at higher taxonomic ranks, while species- and genus-level assignments were predominantly unclassified.

recover thousands of complete and genome-linked BGCs from a single soil metagenome demonstrates the power of ultra-deep long-read sequencing in capturing microbial metabolic potential. In addition, the high novelty rate and wide taxonomic distribution of these clusters emphasise a vast and largely untapped chemical repertoire – one that is unlikely to be captured through shallow

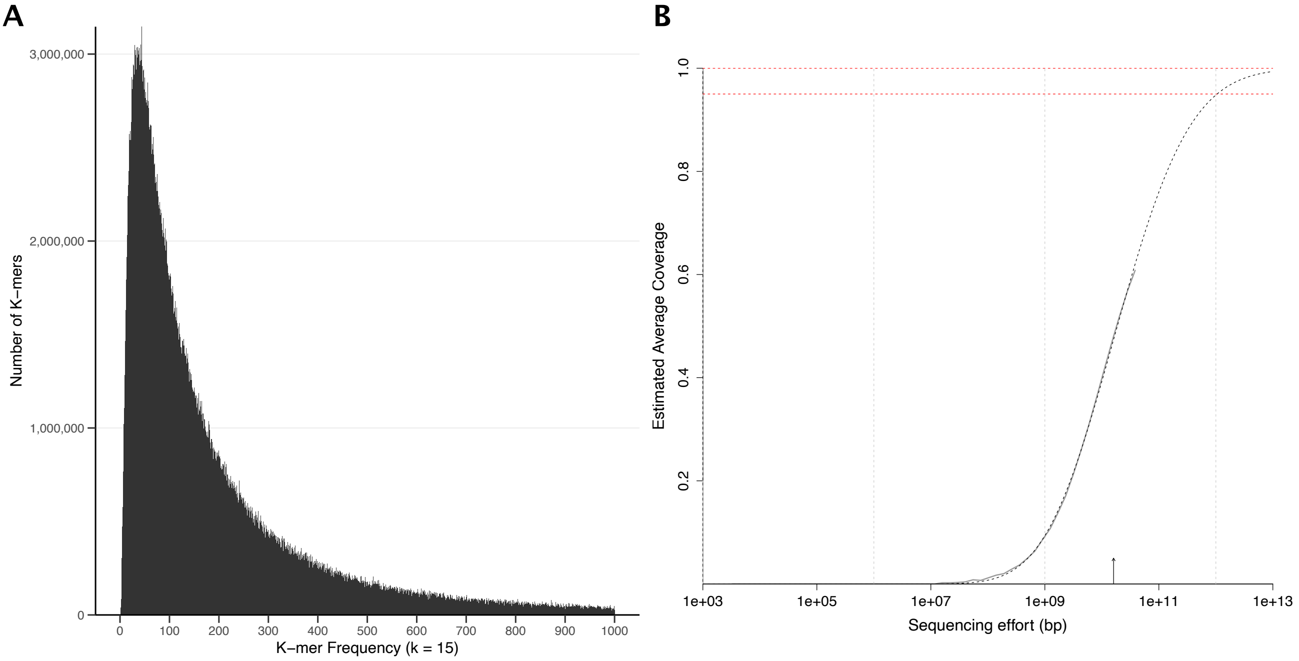


**Figure 3.** Sequencing depth diagnostics show that ultra-deep sequencing still undersamples the total soil diversity. **(A)** 15-mer spectrum of Nanopore reads from the soil metagenome. Bars show the number of distinct 15-mers observed at each copy number. The left-skewed peak at low frequencies reflects a very large pool of unique or low-coverage k-mers, showing extreme sequence diversity. The long right-hand tail originates from higher coverage k-mers, possibly from abundant taxa and repetitive genomic regions. **(B)** Nonpareil curve estimating the sequencing coverage of the combined Illumina dataset. The curve shows the relationship between sequencing effort and estimated coverage, with the solid line representing the observed dataset and the dashed extrapolation indicating expected gains with additional sequencing. The curve suggests that substantially more sequencing effort would be required to cover the whole estimated diversity at least once.

sequencing or cultivation-based surveys alone.

# Discussion

## Soil diversity still outruns ultra-deep sequencing

Our study presents one of the most comprehensive metagenomic investigations of a single soil microbiome to date, combining ultra-deep Oxford Nanopore and Illumina sequencing to yield nearly 300 Gbp of data. This effort enabled high-quality hybrid assemblies and genome-resolved analysis of taxonomic and functional potential in a temperate forest soil sample, providing an empirical benchmark for the capabilities and current limitations of metagenomic sequencing in one of the most complex microbial habitats on Earth.

Despite the ultra-deep sequencing depth, our analyses demonstrate that the microbial diversity of the sample remains far from saturated. Rarefaction curves, k-mer profiles, and 16S rRNA gene and marker gene analyses consistently indicate that an estimated 10 Tbp of sequencing would be required to approach near-complete community recovery. By comparison, MAG recovery in human-gut communities reach saturation with 5-10 Gbp per sample, and 25 Gbp is estimated to be enough even to classify taxa of very low abundances (< 1e-06) [59]. Similarly, Ni et al. [60] estimate this number to be around 7 Gbp to detect gene content of organisms with relative abundances over 1% in human faecal samples.

Ultra-deep campaigns such as Tara Oceans have shown that planktonic marine communities also benefit from multi-terabase sequencing – yielding thousands of novel genes and genomes and greatly refining oceanic reference catalogues. Yet, the incremental discovery of truly new sequence space in the ocean has begun to flatten at these depths [58, 61, 62]. In soils, by contrast, every additional sequencing effort still brings a steeper growth of previously unseen sequence diversity, and MAGs, with no hint of saturation.

## Uncharted novelty of soil microbes and their functional capabilities

Taxonomic profiling of assembled contigs revealed the presence of a diverse range of phyla in the soil, including the expected dominance of well-known soil phyla, such as Acidobacteriota, Actinomycetota, and Pseudomonadota. Yet, over 95% of contigs could not be assigned to a species present currently in databases, and less than 0.7% could be linked to a species with at least one cultured representative. We recovered MAGs that represent candidate novel phyla, highlighting the ability of genome-resolved metagenomics to reveal deeply divergent lineages missed by conventional approaches. Only four MAGs recovered in this study share >= 95% ANI with entries in GTDB, and none correspond to cultured isolates, reaffirming how incompletely current databases represent soil ecosystems. These lineages not only expand the bacterial tree of life but, in some cases, also harbour unique biosynthetic gene clusters, suggesting functional capacities that remain entirely uncharted.

The scale of biosynthetic diversity uncovered is similarly striking. We identified over 11 000 BGCs, more than 5600 of which are complete and many of which could be linked to MAGs. Fewer than 1% of these BGCs matched known families in public databases, suggesting that soils remain a massive and underexplored reservoir for natural product discovery. In other words, the chemistry encoded in a single forest soil sample still sits almost entirely outside the reach of current reference catalogues. The heavy-tailed GCF distribution we observe, where more than 96% of families are singletons, suggests that every additional increment of sequencing continues to reveal functionally distinct BGCs, rather than finding copies of known ones.

These BGCs come from virtually every major bacterial phylum detected in the sample, from well-studied Actinomycetota and Pseudomonadata to under-explored Acidobacteriota, Verrucomicrobiota, and candidate phyla radiation groups. This phylogenetic breadth and the underestimated functional diversity of soil microbes indicate that the soil's metabolic repertoire is not restricted

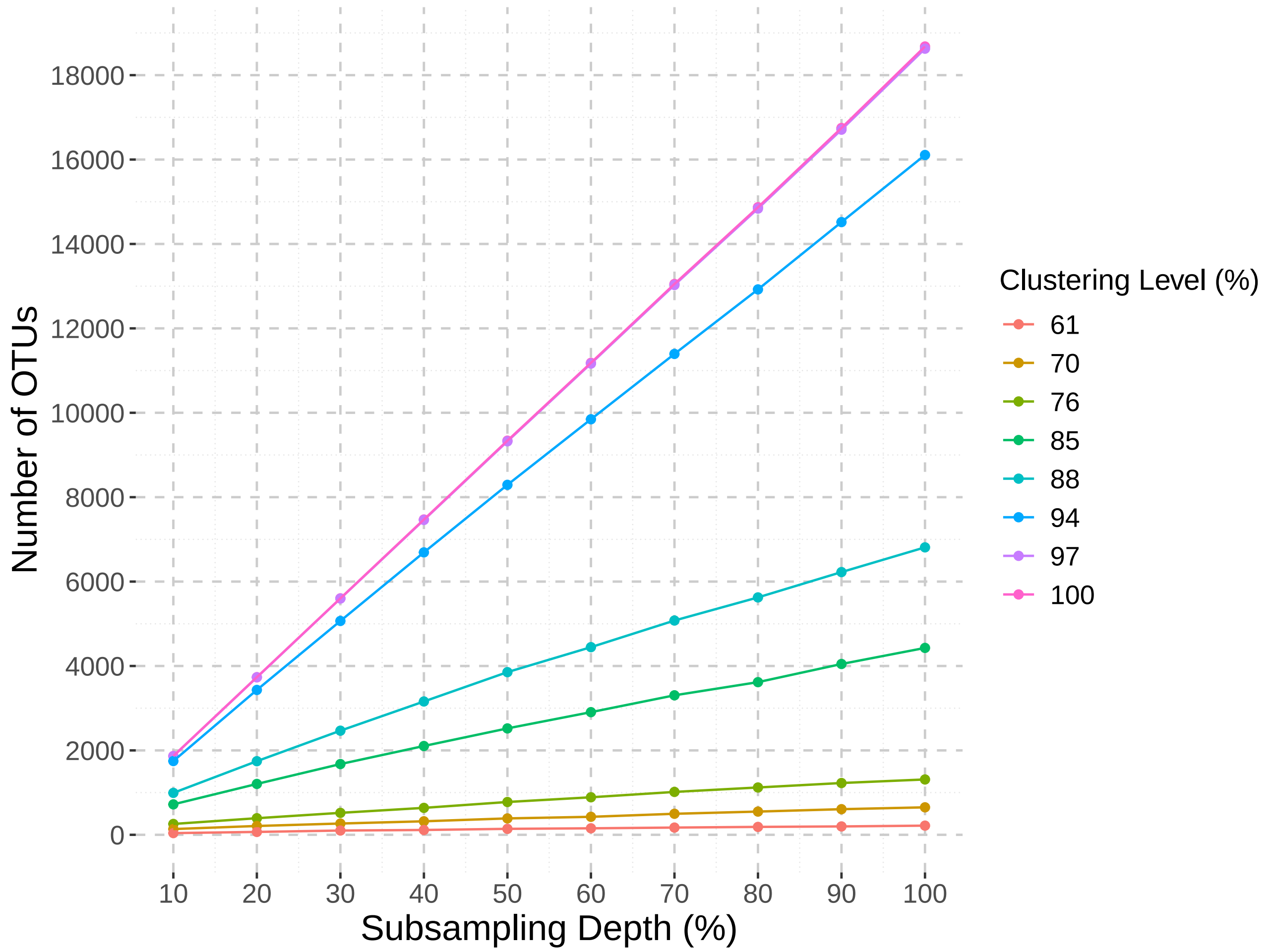


**Figure 4.** Rarefaction curves of 16S rRNA gene sequences at different clustering thresholds. The number of observed operational taxonomic units (OTUs) is plotted against subsampling depth for clustering thresholds ranging from 61% to 100% sequence identity. Higher clustering thresholds (e.g., 97% and 100%) result in a greater number of OTUs detected, gradually increasing by the subsampling depth, while the increase is less steep at lower clustering thresholds. This highlights that the number of observed species is not saturated despite the deep sequencing efforts.

to a few prolific lineages but is dispersed across the microbial tree of life, with implications for microbial interactions, nutrient cycling, defensive traits, and overall ecosystem stability.

Furthermore, our dataset shows that long-read sequencing can address common limitations of metagenomics approaches, including incomplete recovery of BGCs and fragmented assemblies, thereby enabling genome-resolved assessments of secondary metabolism. In contrast to earlier studies depending exclusively on short-read data [53], our approach yields a high proportion of complete BGCs and facilitates the association of biosynthetic capacity with uncultured taxa.

## Conclusions

Together, these results redefine the scale of the unknown in soil microbiomes. They demonstrate that even the deepest sequencing efforts to date barely scratch the surface of microbial and metabolic diversity, particularly in complex environments. Moving forward, terabase- to petabase-scale sequencing will likely be necessary to achieve species-level saturation in soil. However, deeper sequencing alone will not be sufficient. Advances in bioinformatics for assembly, binning, and functional annotation, combined with complementary data from metatranscriptomics, metabolomics, and single-cell approaches, will be essential for translating sequence data into ecological and biochemical insight.

In conclusion, this study sets a new reference point for what is currently achievable in soil metagenomics. By combining ultra-deep sequencing with advanced computational methods, we provide an updated, data-driven view of soil microbial and biosynthetic diversity. The dataset offers an empirical baseline for evaluating sequencing depth, genome recovery, and biosynthetic novelty in complex environments. As such, it can serve as a valuable resource for guiding future studies, benchmarking new tools, and refining diversity estimates. Our findings highlight both the power of current technologies and the need for continued investment in sequencing and analysis to uncover the vast, largely uncharacterised microbial life in soil ecosystems.

## Potential implications

Beyond its soil-ecological focus, this ultra-deep, genome-resolved dataset can (i) act as a benchmark for soil diversity in future studies. (ii) It supplies hundreds of genomes from previously unseen lineages to extend genome databases and taxonomies such as MGnify [63, 64] and GTDB [26], and refine the bacterial tree of life. (iii) It contains >11 000 largely novel and complete BGCs, many linked to host genomes, which can extend the secondary metabolite databases such as BGC Atlas [53] and the SMC [65]; (iv) and this phylogenetically diverse and novel collection of genomes and BGCs can be used as a training set for machine-learning models that predict compound functions [66] and structures [67], and the ecological traits of microbes [68].

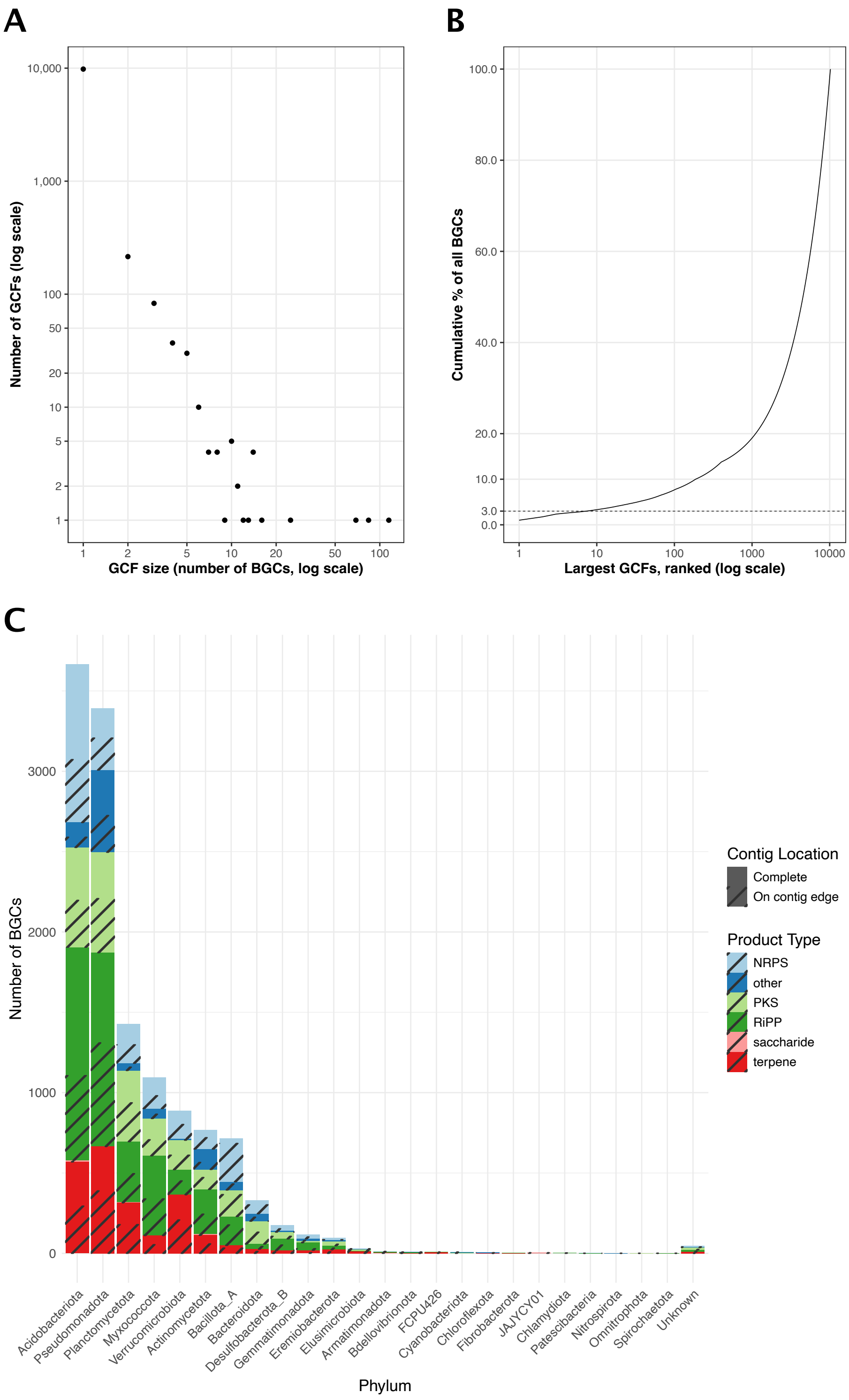


**Figure 5.** The diversity and taxonomic composition of the biosynthetic gene clusters. **(A)** The size distribution of gene cluster families (GCFs). Each dot represents one distinct GCF size in the dataset (11 381 biosynthetic gene clusters grouped into 10 215 GCFs). The x-axis shows the size of the family (number of BGCs), the y-axis shows the number of families that have that exact size; both axes are log10 scaled. The plot reveals a heavy tail: 9813 families (96.1 %) are singletons, only 20 families contain >= 10 BGCs, and the largest family comprises 115 BGCs. **(B)** Cumulative contribution of the largest GCFs to the total BGC dataset. Families are ordered from largest to smallest along the log10-scaled x-axis; the y-axis tracks the running percentage of all 11 381 BGCs accounted for as each additional family is added. The first eight GCFs explain 3.1% of all BGCs, whereas 7939 families are needed to reach 80%. The rapid rise followed by a very shallow tail show that a small handful of families contribute to about 3% of BGCs in the dataset, while thousands of minor families are unique. **(C)** Distribution of biosynthetic gene cluster (BGC) product types across bacterial phyla. Each bar represents the total number of BGCs assigned to a given phylum, coloured by predicted product type. The striped lines indicate the proportion of BGCs that were classified as "on contig edge" by antiSMASH. Phyla are ordered by total BGC count, with "Unknown" indicating contigs for which no confident phylum-level classification could be assigned.

## Data availability

The datasets generated during and/or analysed during the current study are available in the ENA repository, `https://www.ebi.ac.uk/ena/browser/view/PRJEB89893`. The extracted MAGs and identified biosynthetic gene clusters (BGCs) are available at Zenodo (DOI: 10.5281/zenodo.15533781 for the total metagenome assembly and DOI: 10.5281/zenodo.15529477 for MAGs) and in the BGC Atlas repos-

itory at https://bgc-atlas.cs.uni-tuebingen.de/ultra-deep-soil/.

## Additional Files

*Supplementary Fig. S1.* 17-mer spectrum of Illumina reads.

*Supplementary Fig. S2.* 17-mer spectrum of Nanopore reads

*Supplementary Fig. S3.* The number of occurences of each marker gene from bac120 set in raw Nanopore reads.

*Supplementary Table S1.* The details of the extracted MAGs, including their completeness and contamination values, taxonomic classification, assembly statistics, genome annotations statistics, and the number of BGCs identified.

## Declarations

### List of abbreviations

ANI: average nucleotide identity; BGC: biosynthetic gene cluster; bp: base pairs; CPR: Candidate phyla radiation; DNA: deoxyribonucleic acid; ENA: European Nucleotide Archive; Gbp: giga (billion) base pairs; GCF: gene cluster family; GTDB: Genome Taxonomy Database; HQ: high quality; MAG: metagenome-assembled genome; MIBIG: Minimum Information about a Biosynthetic Gene Cluster; MIMAG: Minimum Information about a Metagenome-Assembled Genome; MQ: medium quality; NGS: next-generation sequencing; NRPS: non-ribosomal peptide synthetase; kbp: kilo (thousand) base pairs; Mbp: mega (million) base pairs; ONT: Oxford Nanopore Technologies; OTU: operational taxonomic unit, PKS: polyketide synthase; Q-score: quality score (Phred); RiPP: ribosomally synthesised and post-transcriptionally modified peptide

### Ethical Approval

Not applicable

### Consent for publication

Not applicable

### Competing Interests

The authors declare that they have no competing interests'.

### Funding

This work was supported by Bundesministerium für Bildung und Forschung (BMBF) [161L0284C] to N.Z., Deutsches Zentrum für Infektionsforschung (DZIF) [TTU09.716] to N.Z., and Deutsche Forschungsgemeinschaft (DFG) [INST 37/1049-1] to S.O..

### Author's Contributions

C.B. and N.Z. wrote the main manuscript. C.B. performed the data analysis and prepared the figures. T.N. conducted the soil sampling and DNA isolation. E.A.B. carried out the Nanopore sequencing. C.G. performed basecalling and preliminary quality control of the sequencing data. C.B., S.O., and N.Z. conceptualised the study. All authors reviewed the manuscript.

## Acknowledgements

The authors acknowledge the support by the High Performance and Cloud Computing Group at the Zentrum für Datenverarbeitung of the University of Tübingen and the Federal Ministry of Education and Research (BMBF) through grant no. 031 A535A. We thank the Interfaculty Institute for Biomedical Informatics (IBMI) at the University of Tübingen for providing the computational resources essential for this study. NGS sequencing methods were performed with the support of the DFG-funded NGS Competence Center Tübingen (INST 37/1049-1). Additionally, we thank the Deutsche Forschungsgemeinschaft (DFG, German Research Foundation) under Germany's Excellence Strategy—EXC 2124—390838134 for structural support.

## References

1. Fierer N. Embracing the unknown: disentangling the complexities of the soil microbiome. Nature Reviews Microbiology 2017;15(10):579–590.
2. Roesch LF, Fulthorpe RR, Riva A, Casella G, Hadwin AK, Kent AD, et al. Pyrosequencing enumerates and contrasts soil microbial diversity. The ISME journal 2007;1(4):283–290.
3. Torsvik V, Øvreås L. Microbial diversity and function in soil: from genes to ecosystems. Current opinion in microbiology 2002;5(3):240–245.
4. Jousset A, Bienhold C, Chatzinotas A, Gallien L, Gobet A, Kurm V, et al. Where less may be more: how the rare biosphere pulls ecosystems strings. The ISME journal 2017;11(4):853–862.
5. Lok C. Mining the microbial dark matter. Nature 2015;522(7556):270.
6. Hug LA, Baker BJ, Anantharaman K, Brown CT, Probst AJ, Castelle CJ, et al. A new view of the tree of life. Nature microbiology 2016;1(5):1–6.
7. Lloyd KG, Steen AD, Ladau J, Yin J, Crosby L. Phylogenetically novel uncultured microbial cells dominate earth microbiomes. MSystems 2018;3(5):10–1128.
8. Nascimento Lemos L, Manoharan L, William Mendes L, Monteiro Venturini A, Satler Pylro V, Tsai SM. Metagenome assembled-genomes reveal similar functional profiles of CPR/Patescibacteria phyla in soils. Environmental microbiology reports 2020;12(6):651–655.
9. Nayfach S, Roux S, Seshadri R, Udwary D, Varghese N, Schulz F, et al. A genomic catalog of Earth's microbiomes. Nature biotechnology 2021;39(4):499–509.
10. Parks DH, Rinke C, Chuvochina M, Chaumeil PA, Woodcroft BJ, Evans PN, et al. Recovery of nearly 8,000 metagenome-assembled genomes substantially expands the tree of life. Nature microbiology 2017;2(11):1533–1542.
11. Bertrand D, Shaw J, Kalathiyappan M, Ng AHQ, Kumar MS, Li C, et al. Hybrid metagenomic assembly enables high-resolution analysis of resistance determinants and mobile elements in human microbiomes. Nature biotechnology 2019;37(8):937–944.
12. Van Goethem MW, Osborn AR, Bowen BP, Andeer PF, Swenson TL, Clum A, et al. Long-read metagenomics of soil communities reveals phylum-specific secondary metabolite dynamics. Communications biology 2021;4(1):1302.
13. Agustinho DP, Fu Y, Menon VK, Metcalf GA, Treangen TJ, Sedlazeck FJ. Unveiling microbial diversity: harnessing long-read sequencing technology. Nature methods 2024;21(6):954–966.
14. Wick RR, Judd LM, Holt KE. Assembling the perfect bacterial genome using Oxford Nanopore and Illumina sequencing. PLOS Computational Biology 2023;19(3):e1010905.
15. Zhang T, Li H, Jiang M, Hou H, Gao Y, Li Y, et al. Nanopore sequencing: Flourishing in its teenage years. Journal of Genetics and Genomics 2024;.

16. Mantri SS, Negri T, Sales-Ortells H, Angelov A, Peter S, Neidhardt H, et al. Metagenomic sequencing of multiple soil horizons and sites in close vicinity revealed novel secondary metabolite diversity. Msystems 2021;6(5):10–1128.
17. Negri T, Mantri S, Angelov A, Peter S, Muth G, Eustáquio AS, et al. A rapid and efficient strategy to identify and recover biosynthetic gene clusters from soil metagenomes. Applied Microbiology and Biotechnology 2022;106(8):3293–3306.
18. Chen S. Ultrafast one-pass FASTQ data preprocessing, quality control, and deduplication using fastp. Imeta 2023;2(2):e107.
19. Kolmogorov M, Bickhart DM, Behsaz B, Gurevich A, Rayko M, Shin SB, et al. metaFlye: scalable long-read metagenome assembly using repeat graphs. Nature methods 2020;17(11):1103–1110.
20. Oxford Nanopore Technologies, Medaka: sequence correction provided by ONT Research; 2023. Accessed: 2025-05-26. https://github.com/nanoporetech/medaka.
21. Hu J, Fan J, Sun Z, Liu S. NextPolish: a fast and efficient genome polishing tool for long-read assembly. Bioinformatics 2020;36(7):2253–2255.
22. Li H, Durbin R. Fast and accurate short read alignment with Burrows–Wheeler transform. bioinformatics 2009;25(14):1754–1760.
23. Li H. Minimap2: pairwise alignment for nucleotide sequences. Bioinformatics 2018;34(18):3094–3100.
24. Li D, Liu CM, Luo R, Sadakane K, Lam TW. MEGAHIT: an ultra-fast single-node solution for large and complex metagenomics assembly via succinct de Bruijn graph. Bioinformatics 2015;31(10):1674–1676.
25. Kim J, Steinegger M. Metabuli: sensitive and specific metagenomic classification via joint analysis of amino acid and DNA. Nature Methods 2024;21(6):971–973.
26. Parks DH, Chuvochina M, Rinke C, Mussig AJ, Chaumeil PA, Hugenholtz P. GTDB: an ongoing census of bacterial and archaeal diversity through a phylogenetically consistent, rank normalized and complete genome-based taxonomy. Nucleic acids research 2022;50(D1):D785–D794.
27. Rhie A, Nurk S, Cechova M, Hoyt SJ, Taylor DJ, Altemose N, et al. The complete sequence of a human Y chromosome. Nature 2023;621(7978):344–354.
28. Steinegger M, Söding J. MMseqs2 enables sensitive protein sequence searching for the analysis of massive data sets. Nature biotechnology 2017;35(11):1026–1028.
29. Wang Z, You R, Han H, Liu W, Sun F, Zhu S. Effective binning of metagenomic contigs using contrastive multi-view representation learning. Nature Communications 2024;15(1):585.
30. Liu CC, Dong SS, Chen JB, Wang C, Ning P, Guo Y, et al. MetaDecoder: a novel method for clustering metagenomic contigs. Microbiome 2022;10(1):46.
31. Pan S, Zhao XM, Coelho LP. SemiBin2: self-supervised contrastive learning leads to better MAGs for short-and long-read sequencing. Bioinformatics 2023;39(Supplement_1):i21–i29.
32. Nissen JN, Johansen J, Allesøe RL, Sønderby CK, Armenteros JJA, Grønbech CH, et al. Improved metagenome binning and assembly using deep variational autoencoders. Nature biotechnology 2021;39(5):555–560.
33. Parks DH, Imelfort M, Skennerton CT, Hugenholtz P, Tyson GW. CheckM: assessing the quality of microbial genomes recovered from isolates, single cells, and metagenomes. Genome research 2015;25(7):1043–1055.
34. Hyatt D, Chen GL, LoCascio PF, Land ML, Larimer FW, Hauser LJ. Prodigal: prokaryotic gene recognition and translation initiation site identification. BMC bioinformatics 2010;11:1–11.
35. Sahlin K. Strobealign: flexible seed size enables ultra-fast and accurate read alignment. Genome Biology 2022;23(1):260.
36. Kutuzova S, Piera P, Nor Nielsen K, Olsen NS, Riber L, Gobbi A, et al. Binning meets taxonomy: TaxVAMB improves metagenome binning using bi-modal variational autoencoder. bioRxiv 2024;p. 2024–10.
37. Rühlemann MC, Wacker EM, Ellinghaus D, Franke A. MAGScoT: a fast, lightweight and accurate bin-refinement tool. Bioinformatics 2022;38(24):5430–5433.
38. Eddy SR. Accelerated profile HMM searches. PLoS computational biology 2011;7(10):e1002195.
39. Mistry J, Chuguransky S, Williams L, Qureshi M, Salazar GA, Sonnhammer EL, et al. Pfam: The protein families database in 2021. Nucleic acids research 2021;49(D1):D412–D419.
40. Haft DH, Selengut JD, Richter RA, Harkins D, Basu MK, Beck E. TIGRFAMs and genome properties in 2013. Nucleic acids research 2012;41(D1):D387–D395.
41. Chklovski A, Parks DH, Woodcroft BJ, Tyson GW. CheckM2: a rapid, scalable and accurate tool for assessing microbial genome quality using machine learning. Nature methods 2023;20(8):1203–1212.
42. Ondov BD, Treangen TJ, Melsted P, Mallonee AB, Bergman NH, Koren S, et al. Mash: fast genome and metagenome distance estimation using MinHash. Genome biology 2016;17:1–14.
43. Chaumeil PA, Mussig AJ, Hugenholtz P, Parks DH. GTDB-Tk v2: memory friendly classification with the genome taxonomy database. Bioinformatics 2022;38(23):5315–5316.
44. Letunic I, Bork P. Interactive Tree of Life (iTOL) v6: recent updates to the phylogenetic tree display and annotation tool. Nucleic acids research 2024;52(W1):W78–W82.
45. Mohamadi H, Khan H, Birol I. ntCard: a streaming algorithm for cardinality estimation in genomics data. Bioinformatics 2017;33(9):1324–1330.
46. Rodriguez-R LM, Gunturu S, Tiedje JM, Cole JR, Konstantinidis KT. Nonpareil 3: fast estimation of metagenomic coverage and sequence diversity. MSystems 2018;3(3):10–1128.
47. Seeman T, Barrnap 0.9: rapid ribosomal RNA prediction; 2013. https://github.com/tseemann/barrnap.
48. Rognes T, Flouri T, Nichols B, Quince C, Mahé F. VSEARCH: a versatile open source tool for metagenomics. PeerJ 2016;4:e2584.
49. Schwengers O, Jelonek L, Dieckmann MA, Beyvers S, Blom J, Goesmann A. Bakta: rapid and standardized annotation of bacterial genomes via alignment-free sequence identification. Microbial genomics 2021;7(11):000685.
50. Blin K, Shaw S, Augustijn HE, Reitz ZL, Biermann F, Alanjary M, et al. antiSMASH 7.0: new and improved predictions for detection, regulation, chemical structures and visualisation. Nucleic acids research 2023;51(W1):W46–W50.
51. Navarro-Muñoz JC, Selem-Mojica N, Mullowney MW, Kautsar SA, Tryon JH, Parkinson EI, et al. A computational framework to explore large-scale biosynthetic diversity. Nature chemical biology 2020;16(1):60–68.
52. Zdouc MM, Blin K, Louwen NL, Navarro J, Loureiro C, Bader CD, et al. MIBiG 4.0: advancing biosynthetic gene cluster curation through global collaboration. Nucleic acids research 2025;53(D1):D678–D690.
53. Bağcı C, Nuhamunada M, Goyat H, Ladanyi C, Sehnal L, Blin K, et al. BGC Atlas: a web resource for exploring the global chemical diversity encoded in bacterial genomes. Nucleic acids research 2025;53(D1):D618–D624.
54. Bowers RM, Kyrpides NC, Stepanauskas R, Harmon-Smith M, Doud D, Reddy T, et al. Minimum information about a single amplified genome (MISAG) and a metagenome-assembled genome (MIMAG) of bacteria and archaea. Nature biotechnology 2017;35(8):725–731.
55. Blanco-Míguez A, Beghini F, Cumbo F, McIver LJ, Thompson KN, Zolfo M, et al. Extending and improving metagenomic taxonomic profiling with uncharacterized species using MetaPhlAn 4. Nature Biotechnology 2023;41(11):1633–1644.
56. Chen J, Jia Y, Sun Y, Liu K, Zhou C, Liu C, et al. Global marine microbial diversity and its potential in bioprospecting. Nature 2024;633(8029):371–379.

57. Pasolli E, Asnicar F, Manara S, Zolfo M, Karcher N, Armanini F, et al. Extensive unexplored human microbiome diversity revealed by over 150,000 genomes from metagenomes spanning age, geography, and lifestyle. Cell 2019;176(3):649–662.
58. Sunagawa S, Coelho LP, Chaffron S, Kultima JR, Labadie K, Salazar G, et al. Structure and function of the global ocean microbiome. Science 2015;348(6237):1261359.
59. Kim CY, Lee M, Yang S, Kim K, Yong D, Kim HR, et al. Human reference gut microbiome catalog including newly assembled genomes from under-represented Asian metagenomes. Genome Medicine 2021;13:1–20.
60. Ni J, Yan Q, Yu Y. How much metagenomic sequencing is enough to achieve a given goal? Scientific reports 2013;3(1):1968.
61. Duarte CM, Ngugi DK, Alam I, Pearman J, Kamau A, Eguiluz VM, et al. Sequencing effort dictates gene discovery in marine microbial metagenomes. Environmental Microbiology 2020;22(11):4589–4603.
62. Nishimura Y, Yoshizawa S. The OceanDNA MAG catalog contains over 50,000 prokaryotic genomes originated from various marine environments. Scientific Data 2022;9(1):305.
63. Richardson L, Allen B, Baldi G, Beracochea M, Bileschi ML, Burdett T, et al. MGnify: the microbiome sequence data analysis resource in 2023. Nucleic acids research 2023;51(D1):D753–D759.
64. Gurbich TA, Almeida A, Beracochea M, Burdett T, Burgin J, Cochrane G, et al. MGnify genomes: a resource for biome-specific microbial genome catalogues. Journal of Molecular Biology 2023;435(14):168016.
65. Udwary DW, Doering DT, Foster B, Smirnova T, Kautsar SA, Mouncey NJ. The secondary metabolism collaboratory: a database and web discussion portal for secondary metabolite biosynthetic gene clusters. Nucleic Acids Research 2025;53(D1):D717–D723.
66. Larralde M, Zeller G. Machine learning inference of natural product chemistry across biosynthetic gene cluster types. bioRxiv 2025;p. 2025–03.
67. Skinnider MA, Johnston CW, Gunabalasingam M, Merwin NJ, Kieliszek AM, MacLellan RJ, et al. Comprehensive prediction of secondary metabolite structure and biological activity from microbial genome sequences. Nature communications 2020;11(1):6058.
68. Li Z, Riley WJ, Marschmann GL, Karaoz U, Shirley IA, Wu Q, et al. A framework for integrating genomics, microbial traits, and ecosystem biogeochemistry. Nature Communications 2025;16(1):2186.

manuscript

Click here to access/download;Manuscript;ultra-deep-long-read.pdf

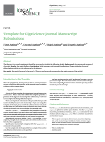

*GigaScience*, 2025, 1–12

doi: xx.xxxx/xxxx
**Manuscript in Preparation**
**Research**

RESEARCH

# Ultra-deep long-read metagenomics captures diverse taxonomic and biosynthetic potential of soil microbes

Caner Bağcı[1,2,3], Timo Negri[1,2,3], Elena Buena Atienza[4,5], Caspar Gross[4,5], Stephan Ossowski[4,5] and Nadine Ziemert[1,2,3,*]

[1]Translational Genome Mining for Natural Products, Interfaculty Institute of Microbiology and Infection Medicine Tübingen (IMIT), University of Tübingen, Auf der Morgenstelle 24, 72076, Tübingen, Baden Württermberg, Germany and [2]Interfaculty Institute for Biomedical Informatics (IBMI), University of Tübingen, Auf der Morgenstelle 24, 72076, Tübingen, Baden Württermberg, Germany and [3]German Center for Infection Research (DZIF), Partner Site Tübingen, Auf der Morgenstelle 24, 72076, Tübingen, Baden Württermberg, Germany and [4]Institute of Medical Genetics and Applied Genomics, University of Tübingen, Calwerstrasse 7, 72076, Tübingen, Baden Württermberg, Germany and [5]NGS Competence Center, University of Tübingen, Calwerstrasse 7, 72076, Tübingen, Baden Württermberg, Germany

[*]nadine.ziemert@uni-tuebingen.de

## Abstract

**Background:** Soil ecosystems have long been recognised as hotspots of microbial diversity, but most estimates of their microbial and functional complexity remain speculative despite decades of study, in part because conventional sequencing campaigns lack the depth and contiguity required to recover low-abundance and repetitive genomes. Here, we revisit this question using one of the deepest metagenomic sequencing efforts to date, applying 148 billion base pairs of Nanopore long-read and 122 billion base pairs of Illumina short-read data to a single forest soil sample.
**Results:** Our hybrid assembly reconstructed 837 metagenome-assembled genomes, including 466 that meet high- and medium-quality standards, nearly all lacking close relatives among cultivated taxa. Rarefaction and k-mer analyses reveal that, even at this depth, we capture only a fraction of the extant diversity: non-parametric models project that more than ten trillion base pairs of sequencing data would be required to approach saturation. These findings offer a quantitative, technology-enabled update to long-standing diversity estimates and demonstrate that conventional metagenomic sequencing efforts likely miss the majority of microbial and biosynthetic potential in soil. We further identify more than 11 000 biosynthetic gene clusters, over 99% of which have no match in current databases, underscoring the breadth of unexplored metabolic capacity.
**Conclusions:** Taken together, our results emphasise both the power and the present limitations of metagenomics in resolving natural microbial complexity, and they provide a new baseline for evaluating future advances in microbial genome recovery, taxonomic classification, and natural product discovery.

**Key words**: microbiome; soil; metagenomics; nanopore; ultra-deep; long-read sequencing; diversity; natural products; (meta)genome mining

## Data Description

In order to quantify how much taxonomic and biosynthetic novelty present-day sequencing can recover from soil, we extracted high-molecular-weight DNA from a single Cambisol forest soil sample (Schönbuch, Germany) and generated an ultra-deep 270 Gbp dataset — 148 Gbp of Oxford Nanopore PromethION long reads (read-length N50 = 12.2 kb) plus 122 Gbp of Illumina NovaSeq

**Compiled on:** July 3, 2025.
Draft manuscript prepared by the author.

**Key Points**

- Ultra-deep hybrid sequencing (148 Gbp Nanopore + 122 Gbp Illumina) of a single forest soil sample yielded 837 metagenome-assembled genomes, all lacking cultured counterparts.
- Despite this unprecedented 270 Gbp depth, rarefaction and coverage modelling indicate that more than 10 Tbp of data would still be needed to reach saturation in soil.
- Only 0.7% and 16.7% of all assembled contigs can be assigned to a species and genus, respectively, with at least one cultured representative, highlighting an unprecedented level of novelty in soil.
- The assembly uncovers 11 381 biosynthetic gene clusters forming over 10 000 mostly novel families, spotlighting an immense, untapped reservoir of microbial natural-product potential.

reads. The hybrid metagenome assembly we conducted produced a 10.5 Gbp assembly, from which multi-tool binning/refinement recovered 837 MAGs, and antiSMASH/BiG-SCAPE annotation revealed >11 000 largely novel biosynthetic gene clusters, creating a resource for benchmarking assembly or binning pipelines, modelling diversity-coverage relationships and mining natural products; all raw reads are archived under ENA BioProject PRJEB89893 and the polished assembly, MAG set and BGC catalogue are available via Zenodo (https://zenodo.org/records/15533781; doi.org/10.5281/zenodo.15529477) for unrestricted reuse.

## Introduction

Soil is one of the most biologically diverse ecosystems on Earth, hosting a massive and largely uncharacterised diversity of microbial life. These microbial communities play critical roles in biogeochemical cycling, soil formation, nutrient turnover, plant health, and climate regulation [1]. Despite their ecological and biotechnological importance, the full extent and functional potential of soil microbiomes remain poorly understood.

A single gram of soil can contain up to $10^9$ microbial cells and hundreds of thousands of species, spanning all domains of life [2, 3]. These communities are not only taxonomically complex but also exhibit highly uneven abundance distributions, where rare taxa – collectively termed the “rare biosphere” – may be functionally consequential despite their low abundance [4].

Although the vast diversity of soil microbes has been widely acknowledged for decades, most estimates of their richness are based on indirect approaches, including 16S rRNA gene surveys, limited shotgun datasets, or predictive modelling. As a result, foundational claims about the extent of soil microbial diversity are frequently cited but rarely re-examined with contemporary data. Much of the soil microbiome remains part of the so-called microbial dark matter: lineages with no cultured representatives and no reference genomes [5, 6, 7].

Metagenomics has enabled the cultivation-independent study of microbial communities, allowing for genome-resolved insights into uncultured organisms [8, 9, 10]. However, most metagenomic studies rely on short-read sequencing, which presents challenges in assembling highly complex, strain-rich communities like soil. These limitations hinder the recovery of low-abundance taxa, reduce the completeness of biosynthetic gene clusters, and often lead to fragmented or ambiguous assemblies [9]. Recent advances in long-read sequencing, particularly with Oxford Nanopore Technologies (ONT), have opened new opportunities for soil microbiome research. Long reads can span repeat-rich regions and operons, improving genome recovery and assembly contiguity [11, 12, 13]. Hybrid approaches combining long and short reads can further mitigate the high error rates of long reads while leveraging their structural advantages [14, 15]. Despite these improvements, it remains unclear how far even the most advanced sequencing technologies can go in capturing the full taxonomic and functional diversity of soil.

To establish an empirical reference point for assessing microbial and biosynthetic diversity in soil, we performed one of the deepest metagenomic sequencing efforts to date on a single soil sample, combining 148 billion base pairs (Gbp) of ONT long-read and 122 Gbp of Illumina short-read data. We use this dataset to empirically assess the power and current limitations of metagenomics in resolving complex microbial communities. Specifically, we ask: How much taxonomic and biosynthetic diversity can be recovered from a single soil sample using state-of-the-art sequencing? What proportion of this diversity is represented in existing databases? Can ultra-deep sequencing approach saturation, or are we still only scratching the surface? By addressing these questions, we aim to provide a data-driven reassessment of microbial diversity in soil and to establish a benchmark for future metagenomic studies of complex ecosystems.

## Methods

### Sample collection, DNA extraction, and metagenomic sequencing

The A horizon of the soil type Cambisol was sampled from the Schönbuch Forest, near Tübingen, Germany (the same site previously reported by [16]), on 31 May 2022. High molecular weight metagenomic DNA was isolated using a protocol described in detail in our previous studies [16, 17]. Genomic integrity was assessed using pulse-field capillary electrophoresis with the Genomic DNA 165 kb Analysis Kit on a FemtoPulse (Agilent) instrument. Quantitation of DNA was assessed using the dsDNA High Sensitivity Assay on a Qubit 3 fluorometer (Thermo Fisher), and purity was assessed by Nanodrop. A total of 2.4 µg of genomic DNA was used as input for the library preparation with the 1D Ligation Kit SQK-LSK109-XL Sequencing Kit (ONT). Four PromethION R9 flow cells were utilised for long-read Nanopore sequencing, each loaded with 600 ng (50 fmol) of genomic DNA. The raw signal data from the PromethION runs were basecalled with guppy (v 5.0.7) in high-accuracy mode. For complementary short-read sequencing, three NovaSeq 6000 flowcells were loaded with the same isolated DNA; two of them ran with 200 cycles, and one with 300 cycles.

A total of 148 Gbp of Nanopore sequencing data was generated, with a read length N50 of 12.2 kb. The three Illumina sequencing runs yielded a total of 122 Gbp raw data, with a mean Q-score of 35. The raw Illumina reads were adapter and quality trimmed using fastp [18] (v 0.23.4) with default settings.

### Metagenomic assembly

Nanopore reads from all four runs were pooled together and assembled using metaFlye (v2.9.5-b1801) [19] with the `-meta` option optimised for metagenomic data, and the `-nano-raw` option for the error-prone Nanopore reads. The resulting draft assem-

bly was polished with one round of medaka [20] (v 2.0.1) using the `r941_prom_hac_g507` model and the `-bacteria` flag. The medaka polished assembly was further corrected using the trimmed Illumina reads in a single round of NextPolish [21] (v 1.4.1), with parameters `-max_depth 100` for short-read mapping with bwa [22] (v 0.7.18), and `-min_read_len 1k -max_depth 100 -x map-ont` for long-read mapping minimap2 [23] (v 2.28-r1209).

In parallel, an Illumina-only assembly was performed using MEGAHIT [24] (v1.2.9) with default metagenomic assembly parameters. All subsequent analyses were based on the hybrid Nanopore-Illumina assembly, which exhibited superior contiguity (contig N50 = 77.8 kb) and total assembled length (10.5 Gbp).

## Taxonomic analysis

Raw reads were taxonomically classified using Metabuli [25] (v 1.0.8), against the precomputed database provided by the authors, which includes GTDB release 214.1 [26] and the human T2T genome [27]. Classification was performed with parameters `-seq-mode 2` for Illumina reads, and `-seq-mode 3` for Nanopore reads. Contigs from the final hybrid assembly were taxonomically classified using MMSeqs2 [28] (v 16.747c6) in `easy-taxonomy` mode, with GTDB release 214.1 [26].

## Reconstruction of metagenome-assembled genomes

Metagenome-assembled genomes(MAGs) were recovered using a combination of complementary binning strategies to maximise genome quality. Binning was first performed independently on the final NextPolish-corrected assembly using COMEBin [29], MetaDecoder [30], SemiBin2 [31], VAMB [32], executed in a CUDA-enabled environment when possible.

COMEBin (v 1.0.4), together with CheckM (v 1.1.3) [33], was run on mapping files generated by minimap2 [23], with the following parameters: `NUM_VIEWS=6`, `TEMPERATURE=0.07`, `EMBEDDING_SIZE=2048`, `COVERAGE_EMBEDDING_SIZE=2048`, and `BATCH_SIZE=1024`. MetaDecoder (v 1.1.0) was executed with default parameters according to the authors' recommendations. SemiBin2 (v 2.1.0) was run in both `-self-supervised` and `-semi-supervised` modes, specifying soil as the environment type and `long_reads` as the sequencing type. Gene prediction was performed using Prodigal (v 2.6.3) [34], and the GTDB v95 database [26] was used alongside the previously described minimap2 mappings of the long reads.

For VAMB (v 4.1.4) [32], six binning results were generated by combining default and taxonomy-aware binning modes with three input configurations: long reads, short reads, and both. Abundance profiles were computed using Strobealign (v 0.15.0) [35]. For TaxVAMB [36], contig-level taxonomic annotations were derived using Metabuli [25] against the GTDB reference database [26].

In order to produce a final, unified metagenome-assembled-genome binning, the binning results from the above-mentioned ten runs were collected, and refined using MAGScoT [37] (v 1.1). MAGScot was run on gene-calling results from prodigal [34] (v 2.6.3), and hmmsearch results from the HMMER package (v 2.6.3) [38], using domain profiles from PFAM [39] and TIGRFAMs [40] databases.

The resulting MAGs were evaluated for their completeness and contamination with CheckM2 [41] (v 1.0.2). Pairwise genomic distances were computed using Mash [42] (v 2.3) with default parameters. Taxonomic assignments were performed with GTDB-Tk [43] (v 2.4.0) using the GTDB r220 release [26] in classify mode. A phylogenetic tree of high- and medium-quality MAGs was constructed with GTDB-Tk in *de novo* mode and visualised with iTOL [44].

## Rarefaction analysis

Microbial diversity and sequencing coverage were assessed using multiple complementary approaches. K-mer frequency analysis was conducted with ntCard [45] using 15-mers and 17-mers for both Nanopore and Illumina datasets. Nonpareil [46] (v3.3.3) was used to estimate sequence coverage and to project additional sequencing efforts required for complete community recovery. Full-length 16S rRNA gene sequences were extracted from Nanopore reads using Barrnap [47] (v0.9), and clustered into operational taxonomic units (OTU) at various identity thresholds (100%, 97%, 95%, etc.) using VSEARCH [48] (v2.15.2). Rarefaction curves were constructed by subsampling reads and calculating cumulative OTU counts. In addition, conserved single-copy marker genes from the bac120 set [10] were identified in Nanopore reads using LAST aligner (v 1615) in frameshify mode (`-F 15`) against the GTDB bac120 reference set, and quantified to independently assess the taxonomic diversity.

## Annotation, detection, and clustering of biosynthetic gene clusters

The final assembly and refined MAGs were annotated using Bakta [49] (v 1.11.0, database version 5.1). Biosynthetic gene clusters (BGCs) were identified using antiSMASH [50] (v7) with the options `-fullhmmer -clusterhmmer -tigrfam -asf -cc-mibig -cb-general -cb-subclusters -cb-knownclusters -pfam2go -rre -smcog-trees -tfb`. Identified BGCs were subsequently clustered into gene cluster families (GCFs) using BiG-SCAPE [51] (v2 beta5), based on domain architecture and sequence similarity. The novelty of BGCs was assessed by comparisons against the MIBiG 4.0 [52] and BGC Atlas [53] databases.

# Results

## Ultra-deep hybrid sequencing of a temperate forest sample

To evaluate the limits of current sequencing technologies in capturing soil microbial diversity, we generated an ultra-deep metagenomic dataset from a single sample collected in the Schönbuch forest, a temperate mixed forest in southwestern Germany. The sample was taken from the A horizon of a Cambisol soil, a type of soil known for its rich microbial diversity and favourable characteristics for DNA extraction. This particular site and soil type were previously shown to harbour high biosynthetic potential and taxonomic richness [16], and were therefore selected as a representative model system for deep metagenomic exploration.

We extracted total environmental DNA from this soil and sequenced it using a hybrid strategy that combined 148 Gbp of ONT long-read and 122 Gbp of Illumina short-read sequencing. Nanopore reads had a read length N50 of 12.2 kb, while Illumina reads achieved a mean Q-score of 35. Together, this 270 Gbp dataset represents one of the deepest single-sample soil metagenomes reported to date, and provides a unique opportunity to empirically assess taxonomic and functional complexity.

## Hybrid assembly recovers hundreds of genomes with high taxonomic and biosynthetic novelty

We employed a hybrid assembly strategy to combine the complementary strengths of long and short reads. Long-read Nanopore data were assembled and subsequently polished using Illumina data, resulting in 10.5 Gbp of assembled sequence with a contig N50 of 77.8 kb. For comparison, an Illumina-only assembly produced a more fragmented assembly with an N50 of only 865 bp and a total length of 9.2 Gbp, confirming the advantages of long-read

inclusion.

From the hybrid assembly, we recovered 837 metagenome-assembled genomes (MAGs), of which 466 met the MIMAG high- or medium-quality criteria [54]. All MAGs recovered represented unique species-level units, defined by > 5% pairwise Mash distance. These genomes spanned a wide range of bacterial phyla typical of soils, including Acidobacteriota, Pseudomonadota, Verrucomicrobiota, Actinomycetota, and Myxococcota. Remarkably, only four of the 837 MAGs had species-level matches (>= 95% ANI) in the GTDB database, and none had cultured representatives.

Thirteen MAGs were assigned to phyla with no cultured members, such as Desulfobacterota, and candidate phyla radiation (CPR) groups FCPU426 and JAJYCY01, harbouring between 2 and 19 BGCs (Figure 1). At other taxonomic levels, 9, 45, 155, and 611 MAGs belong to novel lineages that correspond to class, order, family, and genus levels, respectively.

High- and medium-quality MAGs ranged in size from 1 Mbp (Patescibacteria) to 15 Mbp (Planctomycetota) (Figure 1 and Supplementary Table S1).

Many of the recovered MAGs are assigned to taxonomic groups that do not correspond to any known families or even classes in current reference databases (Figure 1). This points to a substantial fraction of microbial diversity in soil that remains undescribed, even at higher taxonomic ranks. In addition to their taxonomic novelty, many of these genomes contain a large number of BGCs, in some cases reaching 30 distinct clusters within a single genome. This suggests that the potential for secondary metabolite production is not restricted to well-characterised soil taxa (such as Actinomycetota), but is also widespread among uncharacterised lineages.

## Taxonomic analysis reveals unprecedented levels of novelty

Taxonomic classification of both the raw reads and the assembled contigs revealed a highly diverse and complex microbial community, comprising members from 69 different phyla. As expected for soil environments, the community was dominated by taxa such as Pseudomonodata and Acidobacteriota (Figure 2A), along with representatives from many other phyla. A small fraction (approximately 1%) of the reads originated from eukaryotes, while 8% remained unclassified, indicating the presence of deeply novel sequences absent from current databases.

Taxonomic analysis of the assembled contigs (Figure 2B) further demonstrated that a substantial portion of the dataset originates from uncultivated organisms across all taxonomic ranks. While soil has long been recognised as a reservoir of uncultivated diversity, with historical estimates suggesting 90–99% of taxa remain uncultured, our data now provide quantitative, genome-resolved confirmation of this. At the species level, more than 99% of the assembled contigs could not be assigned to any taxon with a cultured representative. Only 0.7% of contigs could be linked to species with at least one cultured strain, and 16.7% to a genus with cultured members.

Even at broader taxonomic levels, substantial novelty is evident: 9% of contigs could not be assigned to any known class, and 6.2% could not be linked to any known phylum (Figure 2B). Moreover, for those contigs that were classified, the majority were affiliated with taxa composed exclusively of uncultivated representatives, often known only from metagenomic or single-cell sequencing studies. These patterns are in contrast to metagenomic datasets from more tractable environments, such as the human gut or ocean microbiomes, where a larger fraction of genomes can be linked to cultivated taxa [55, 56].

Despite the expansion of public genome collections such as GTDB [26] and the GEM catalog [9], fewer than five of the 837 MAGs recovered in this study share at least 95% ANI with any known species. This confirms that soil remains one of the most genomically undercharacterised microbial ecosystems.

## Soil diversity remains undersampled

To evaluate whether ultra-deep sequencing could fully capture the microbial diversity present in the sample, we applied a combination of k-mer analysis, rarefaction based on marker genes and full-length 16S rRNA genes. All approaches consistently indicated that, despite the unprecedented depth of sequencing, the dataset remains far from saturating the underlying biological diversity.

K-mer analysis, which examines the frequency distribution of short subsequences within the sequencing reads, revealed a strong left-skewed distribution (Figure 3A). The majority of k-mers occurred only once or a few times, indicating a high prevalence of unique or rare sequences. This pattern is characteristic of highly diverse communities and reflects the presence of many low-abundance taxa whose genomes were either incompletely covered or missed entirely in assembly. A long right-hand tail, representing high-frequency k-mers, likely originates from abundant taxa or repetitive genomic elements (such as 16S rRNA genes). These patterns were observed both in Nanopore and Illumina datasets, and are consistent with extreme sequence heterogeneity of the sample (Figure 3A, and Supplementary Figures 1 and 2).

Rarefaction analysis using Nonpareil further supported this conclusion. By estimating the redundancy of reads, the model projected that approximately 10 terabases of sequencing data would be necessary to achieve near-complete coverage of the microbial diversity in this single soil sample — a nearly 50-fold increase over the current deep-sequencing effort (Figure 3B). This analysis confirms that even the most comprehensive sequencing efforts to date fall short of fully resolving the taxonomic complexity of soil microbiomes.

In parallel, we extracted full-length 16S rRNA gene sequences from Nanopore reads and clustered them into operational taxonomic units (OTUs) at various sequence identity thresholds. At 100% and 97% identity, the number of observed OTUs continued to increase linearly with sequencing depth, showing no indication of saturation (Figure 4A). While higher-order groupings (e.g., family or phylum) began to plateau, even genus-level rarefaction curves remained unsaturated. A complementary analysis based on 120 conserved single-copy bacterial marker genes showed similar trends. For each marker gene, we detected between 9000 and 59 000 unique copies across the dataset (Supplementary Figure 3).

Taken together, these results provide robust and multi-dimensional evidence that the soil microbial community remains vastly undersampled, even with sequencing efforts far beyond typical practice. The failure to reach saturation was especially apparent at the species level, where both OTU-based and marker gene rarefaction curves continued to rise linearly across all subsampling depths. This indicates an exceptional degree of fine-scale taxonomic resolution, with many closely related but genomically distinct lineages present at low abundance.

Such strain- and species-level diversity is particularly important given that many ecologically and functionally relevant traits – such as niche specialisation, symbiosis, and secondary metabolite biosynthesis – can differ even between closely related strains. In contrast to more constrained environments such as the human gut, where species-level diversity tends to plateau with a few hundred genomes [57], or marine planktonic communities, which show relatively stable core microbiomes [58], the soil microbiome appears to exhibit near-limitless diversity at the genomic level.

These findings not only demonstrate the limitations of current sequencing depths but also call into question the accuracy of historical species richness estimates derived from much shallower data. They suggest that achieving anything approaching species-level saturation in soil will likely require terabase- to petabase-scale sequencing efforts, combined with refined computational methods to resolve low-abundance and highly diverse taxa. They demon-

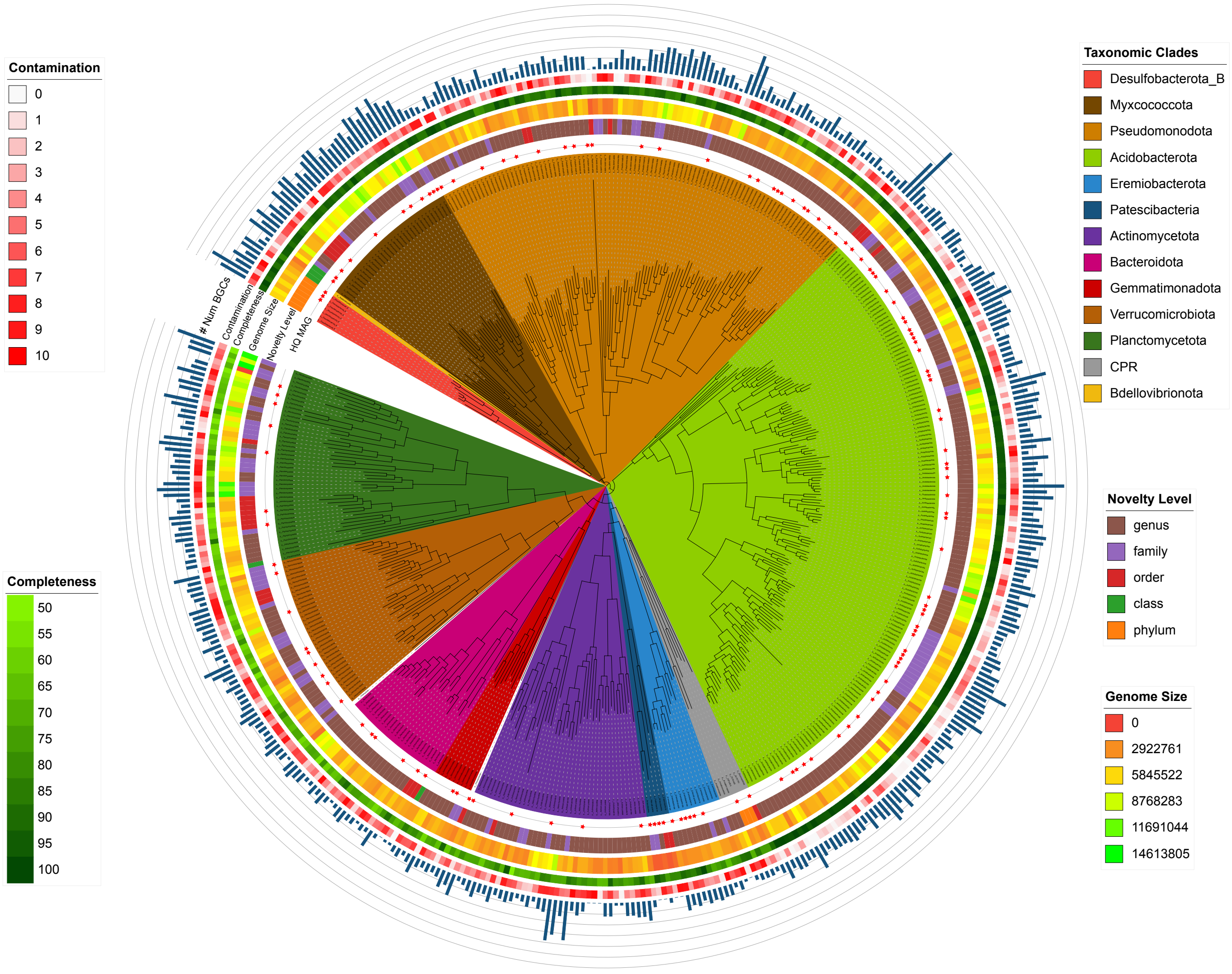


**Figure 1.** Genome quality statistics and taxonomic classification of newly reconstructed high-quality and medium-quality metagenome-assembled genomes (MAGs). Each leaf represents a MAG, coloured by its assigned phylum. Concentric rings around the leaves indicate, from innermost to outermost: (i) HQ MAGs, (ii) novelty level (genus to phylum), (iii) genome size (bp), (iv) completeness (%), (v) contamination (%), and (vi) the number of BGCs encoded by the MAG (range: 0-30).

strate that many of the diversity estimates historically used in soil microbiome research, often based on much shallower sequencing, likely underestimate the true scale of taxonomic complexity.

## Long-read metagenomics recovers thousands of novel and genome-linked BGCs

From the hybrid metagenomic assembly, we identified a total of 11 381 BGCs, including 5652 classified as complete, reflecting the improved contiguity made possible through long-read sequencing. This proportion of complete clusters (~50%) represents a substantial improvement over previous metagenomic surveys, where fewer than 10% of BGCs are typically annotated as complete [53].

Clustering the identified BGCs with BiG-SCAPE resulted in 10 215 gene cluster families, indicating that most BGCs in our dataset are non-redundant and functionally distinct. The distribution of GCF sizes (Figure 5A) was strongly skewed: most families consisted of singletons or doubletons, with only a handful of larger families, further illustrating the functional uniqueness and fine-grained metabolic specialisation within the community. The largest GCF contains 115 ribosomally synthesised and post-transcriptionally modified peptide (RiPP) BGCs. The cumulative ordering of the GCFs by size shows that the largest eight families account for 3.1% of all BGCs, whereas each of the remaining over ten thousand families contributes only very little to the total number (Figure 5B).

Taxonomic classification of the BGC-containing contigs revealed that biosynthetic potential is broadly distributed across the phylogenetic spectrum. While well-known producers such as Actinomycetota and Pseudomonadata were represented, we also detected numerous BGCs in less-studied phyla such as Verrucomicrobiota and Acidobacteriota (Figure 5C). These groups have historically been overlooked due to cultivation barriers but appear to harbour substantial secondary metabolic capacity.

Only 108 BGCs (~ 1%) had significant matches to known GCFs in the BGC Atlas database, which compiles nearly two million BGCs from metagenomic sources. This confirms earlier observations that soil metagenomes are rich in biosynthetic novelty [12, 51], but also demonstrates that even with massive reference expansions, a typical soil still contains an overwhelming majority of uncharacterised BGCs. Notably, our dataset contains more novel BGCs than reported in most large-scale studies of marine or human microbiomes, where the rate of novel BGC discovery is often constrained by lower microbial diversity and shorter contigs [51, 53].

Of all BGCs identified, 6279 could be linked to metagenome-assembled genomes, enabling genome-resolved exploration of natural product potential. This represents one of the largest genome-resolved inventories of BGCs from a single environmental sample to date.

Together, these results point to a biosynthetic landscape in soil that is both expansive and largely underexplored. The ability to

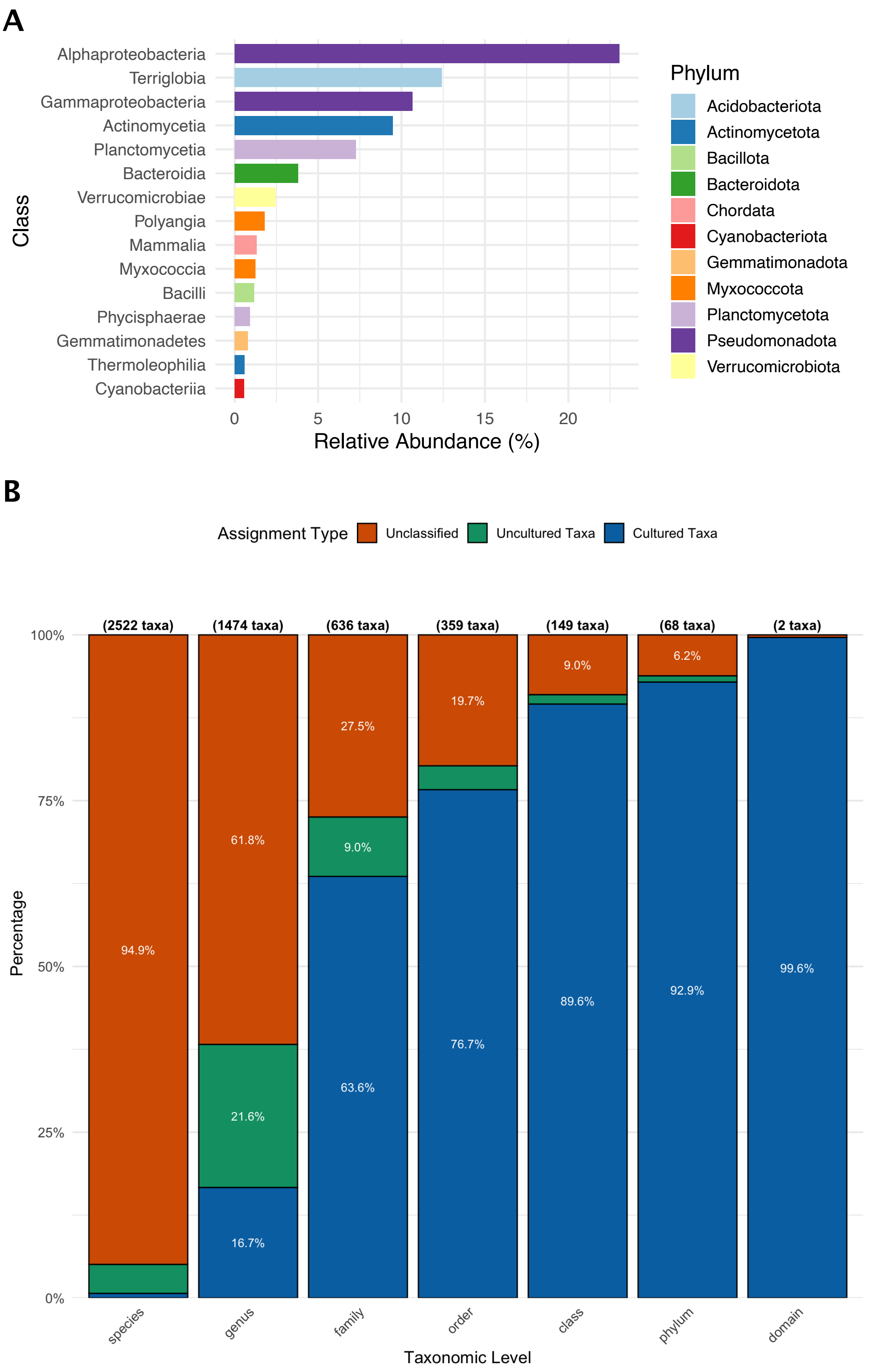


**Figure 2.** Taxonomic summary of the assembled contigs. **(A)** Class-level relative abundances of the assembled contigs, coloured by the phyla they belong to. **(B)** Taxonomic novelty levels of the assembled contigs across different ranks. Bars represent the proportion of contigs assigned to cultured taxa (blue), uncultured taxa (only observed in other metagenome studies) (green), or remaining unclassified (completely novel) (orange) at each taxonomic level, from species to domain. The number of taxa identified at each level is shown above each bar. Most contigs could only be classified at higher taxonomic ranks, while species- and genus-level assignments were predominantly unclassified.

recover thousands of complete and genome-linked BGCs from a single soil metagenome demonstrates the power of ultra-deep long-read sequencing in capturing microbial metabolic potential. In addition, the high novelty rate and wide taxonomic distribution of these clusters emphasise a vast and largely untapped chemical repertoire – one that is unlikely to be captured through shallow

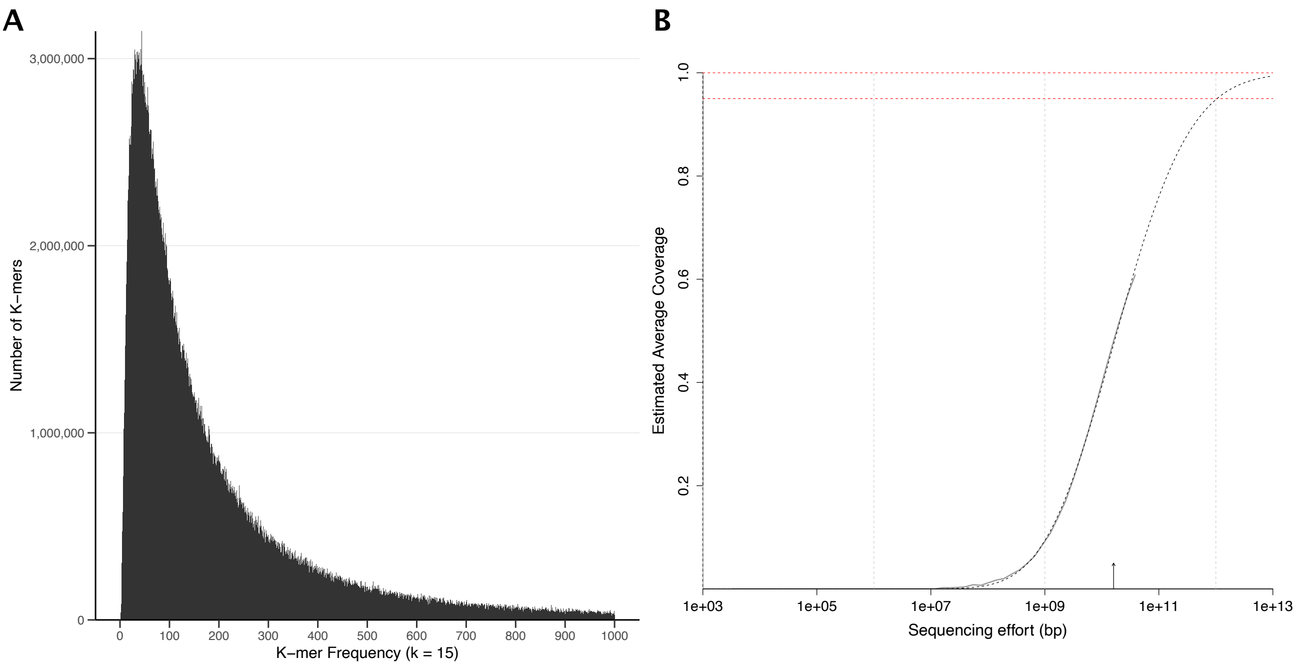


**Figure 3.** Sequencing depth diagnostics show that ultra-deep sequencing still undersamples the total soil diversity. **(A)** 15-mer spectrum of Nanopore reads from the soil metagenome. Bars show the number of distinct 15-mers observed at each copy number. The left-skewed peak at low frequencies reflects a very large pool of unique or low-coverage k-mers, showing extreme sequence diversity. The long right-hand tail originates from higher coverage k-mers, possibly from abundant taxa and repetitive genomic regions. **(B)** Nonpareil curve estimating the sequencing coverage of the combined Illumina dataset. The curve shows the relationship between sequencing effort and estimated coverage, with the solid line representing the observed dataset and the dashed extrapolation indicating expected gains with additional sequencing. The curve suggests that substantially more sequencing effort would be required to cover the whole estimated diversity at least once.

sequencing or cultivation-based surveys alone.

# Discussion

## Soil diversity still outruns ultra-deep sequencing

Our study presents one of the most comprehensive metagenomic investigations of a single soil microbiome to date, combining ultra-deep Oxford Nanopore and Illumina sequencing to yield nearly 300 Gbp of data. This effort enabled high-quality hybrid assemblies and genome-resolved analysis of taxonomic and functional potential in a temperate forest soil sample, providing an empirical benchmark for the capabilities and current limitations of metagenomic sequencing in one of the most complex microbial habitats on Earth.

Despite the ultra-deep sequencing depth, our analyses demonstrate that the microbial diversity of the sample remains far from saturated. Rarefaction curves, k-mer profiles, and 16S rRNA gene and marker gene analyses consistently indicate that an estimated 10 Tbp of sequencing would be required to approach near-complete community recovery. By comparison, MAG recovery in human-gut communities reach saturation with 5-10 Gbp per sample, and 25 Gbp is estimated to be enough even to classify taxa of very low abundances (< 1e-06) [59]. Similarly, Ni et al. [60] estimate this number to be around 7 Gbp to detect gene content of organisms with relative abundances over 1% in human faecal samples.

Ultra-deep campaigns such as Tara Oceans have shown that planktonic marine communities also benefit from multi-terabase sequencing – yielding thousands of novel genes and genomes and greatly refining oceanic reference catalogues. Yet, the incremental discovery of truly new sequence space in the ocean has begun to flatten at these depths [58, 61, 62]. In soils, by contrast, every additional sequencing effort still brings a steeper growth of previously unseen sequence diversity, and MAGs, with no hint of saturation.

## Uncharted novelty of soil microbes and their functional capabilities

Taxonomic profiling of assembled contigs revealed the presence of a diverse range of phyla in the soil, including the expected dominance of well-known soil phyla, such as Acidobacteriota, Actinomycetota, and Pseudomonadota. Yet, over 95% of contigs could not be assigned to a species present currently in databases, and less than 0.7% could be linked to a species with at least one cultured representative. We recovered MAGs that represent candidate novel phyla, highlighting the ability of genome-resolved metagenomics to reveal deeply divergent lineages missed by conventional approaches. Only four MAGs recovered in this study share >= 95% ANI with entries in GTDB, and none correspond to cultured isolates, reaffirming how incompletely current databases represent soil ecosystems. These lineages not only expand the bacterial tree of life but, in some cases, also harbour unique biosynthetic gene clusters, suggesting functional capacities that remain entirely uncharted.

The scale of biosynthetic diversity uncovered is similarly striking. We identified over 11 000 BGCs, more than 5600 of which are complete and many of which could be linked to MAGs. Fewer than 1% of these BGCs matched known families in public databases, suggesting that soils remain a massive and underexplored reservoir for natural product discovery. In other words, the chemistry encoded in a single forest soil sample still sits almost entirely outside the reach of current reference catalogues. The heavy-tailed GCF distribution we observe, where more than 96% of families are singletons, suggests that every additional increment of sequencing continues to reveal functionally distinct BGCs, rather than finding copies of known ones.

These BGCs come from virtually every major bacterial phylum detected in the sample, from well-studied Actinomycetota and Pseudomonadata to under-explored Acidobacteriota, Verrucomicrobiota, and candidate phyla radiation groups. This phylogenetic breadth and the underestimated functional diversity of soil microbes indicate that the soil's metabolic repertoire is not restricted

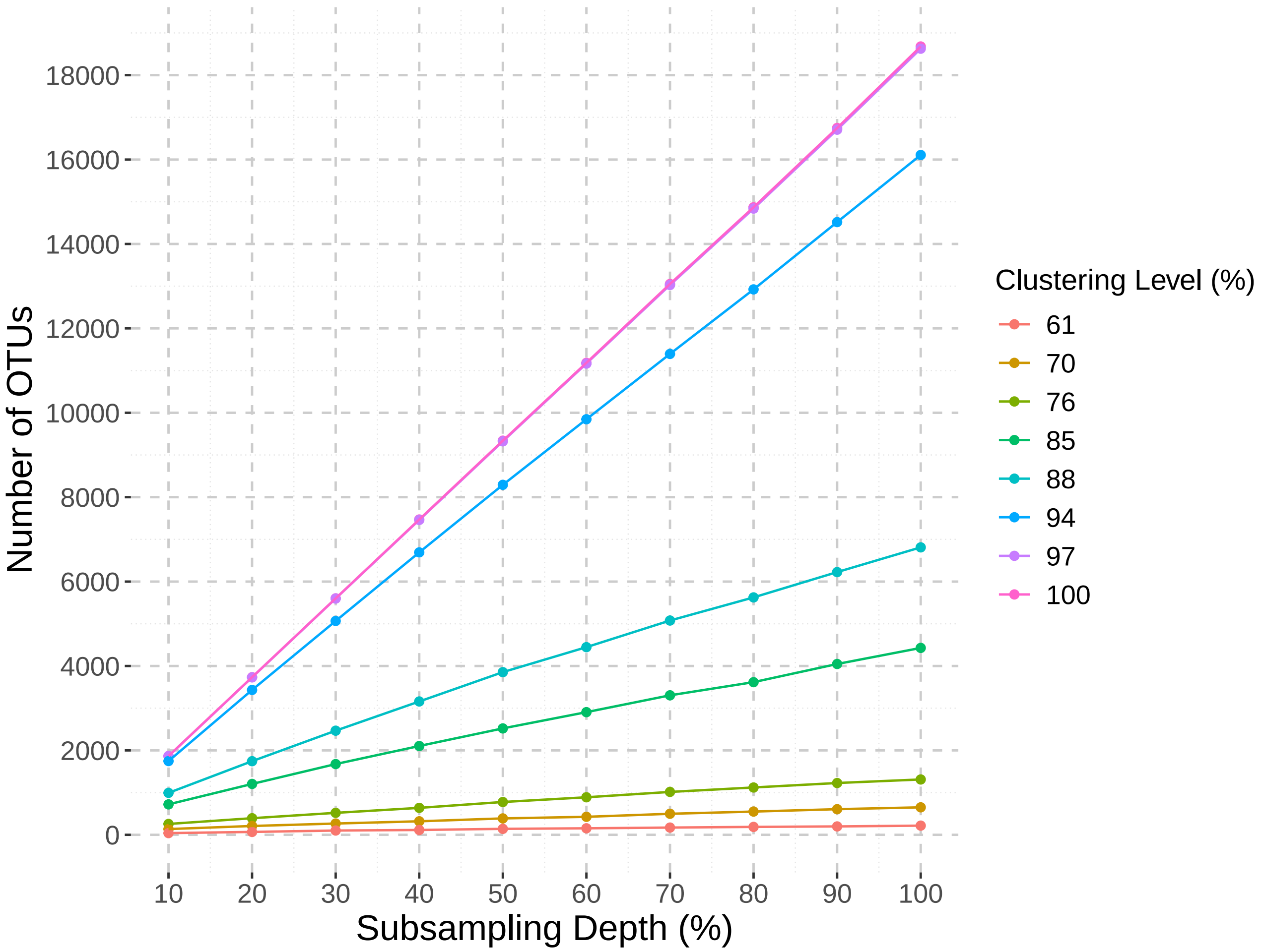


**Figure 4.** Rarefaction curves of 16S rRNA gene sequences at different clustering thresholds. The number of observed operational taxonomic units (OTUs) is plotted against subsampling depth for clustering thresholds ranging from 61% to 100% sequence identity. Higher clustering thresholds (e.g., 97% and 100%) result in a greater number of OTUs detected, gradually increasing by the subsampling depth, while the increase is less steep at lower clustering thresholds. This highlights that the number of observed species is not saturated despite the deep sequencing efforts.

to a few prolific lineages but is dispersed across the microbial tree of life, with implications for microbial interactions, nutrient cycling, defensive traits, and overall ecosystem stability.

Furthermore, our dataset shows that long-read sequencing can address common limitations of metagenomics approaches, including incomplete recovery of BGCs and fragmented assemblies, thereby enabling genome-resolved assessments of secondary metabolism. In contrast to earlier studies depending exclusively on short-read data [53], our approach yields a high proportion of complete BGCs and facilitates the association of biosynthetic capacity with uncultured taxa.

## Conclusions

Together, these results redefine the scale of the unknown in soil microbiomes. They demonstrate that even the deepest sequencing efforts to date barely scratch the surface of microbial and metabolic diversity, particularly in complex environments. Moving forward, terabase- to petabase-scale sequencing will likely be necessary to achieve species-level saturation in soil. However, deeper sequencing alone will not be sufficient. Advances in bioinformatics for assembly, binning, and functional annotation, combined with complementary data from metatranscriptomics, metabolomics, and single-cell approaches, will be essential for translating sequence data into ecological and biochemical insight.

In conclusion, this study sets a new reference point for what is currently achievable in soil metagenomics. By combining ultra-deep sequencing with advanced computational methods, we provide an updated, data-driven view of soil microbial and biosynthetic diversity. The dataset offers an empirical baseline for evaluating sequencing depth, genome recovery, and biosynthetic novelty in complex environments. As such, it can serve as a valuable resource for guiding future studies, benchmarking new tools, and refining diversity estimates. Our findings highlight both the power of current technologies and the need for continued investment in sequencing and analysis to uncover the vast, largely uncharacterised microbial life in soil ecosystems.

## Potential implications

Beyond its soil-ecological focus, this ultra-deep, genome-resolved dataset can (i) act as a benchmark for soil diversity in future studies. (ii) It supplies hundreds of genomes from previously unseen lineages to extend genome databases and taxonomies such as MGnify [63, 64] and GTDB [26], and refine the bacterial tree of life. (iii) It contains >11 000 largely novel and complete BGCs, many linked to host genomes, which can extend the secondary metabolite databases such as BGC Atlas [53] and the SMC [65]; (iv) and this phylogenetically diverse and novel collection of genomes and BGCs can be used as a training set for machine-learning models that predict compound functions [66] and structures [67], and the ecological traits of microbes [68].

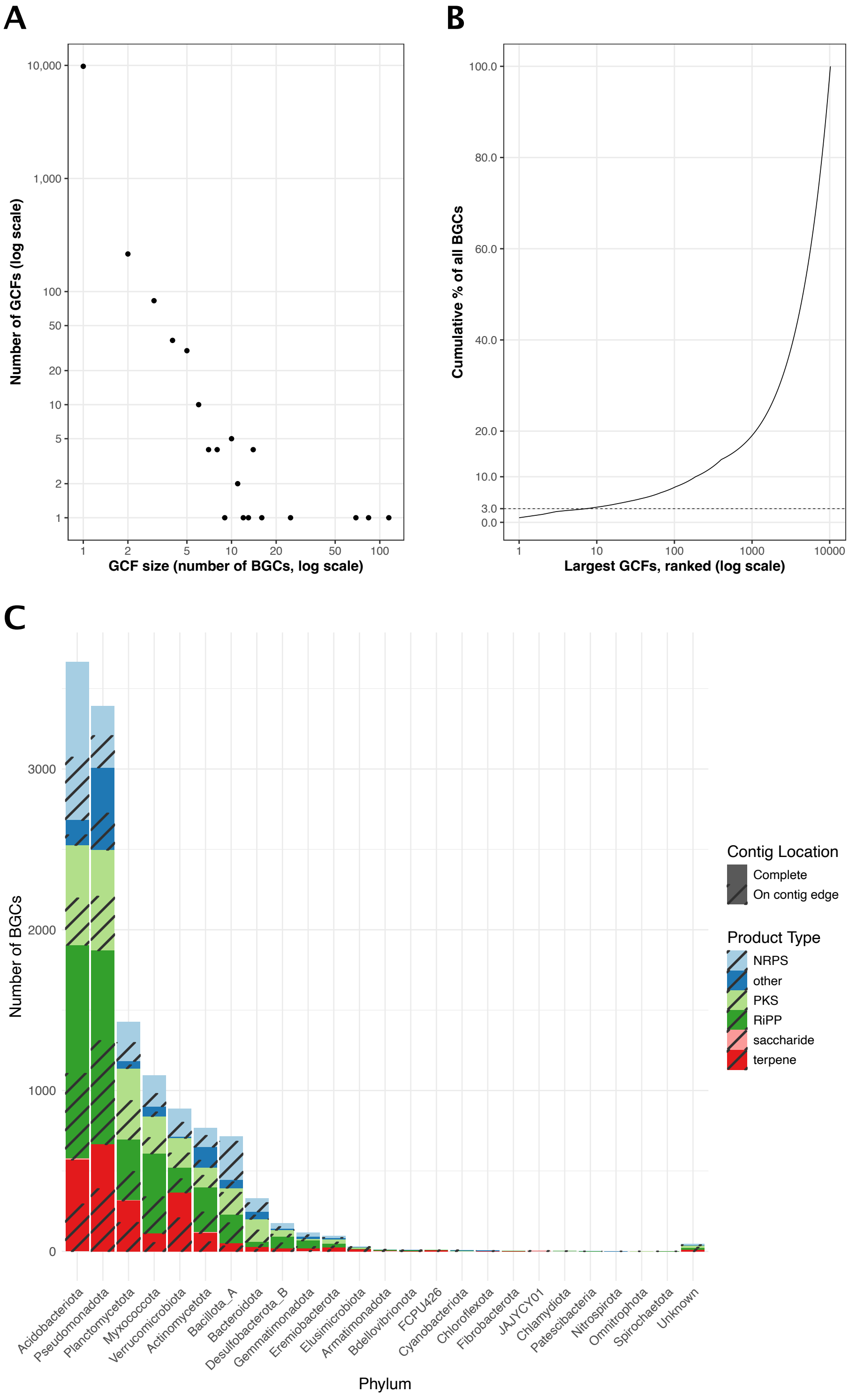


**Figure 5.** The diversity and taxonomic composition of the biosynthetic gene clusters. **(A)** The size distribution of gene cluster families (GCFs). Each dot represents one distinct GCF size in the dataset (11 381 biosynthetic gene clusters grouped into 10 215 GCFs). The x-axis shows the size of the family (number of BGCs), the y-axis shows the number of families that have that exact size; both axes are log10 scaled. The plot reveals a heavy tail: 9813 families (96.1 %) are singletons, only 20 families contain >= 10 BGCs, and the largest family comprises 115 BGCs. **(B)** Cumulative contribution of the largest GCFs to the total BGC dataset. Families are ordered from largest to smallest along the log10-scaled x-axis; the y-axis tracks the running percentage of all 11 381 BGCs accounted for as each additional family is added. The first eight GCFs explain 3.1% of all BGCs, whereas 7939 families are needed to reach 80%. The rapid rise followed by a very shallow tail show that a small handful of families contribute to about 3% of BGCs in the dataset, while thousands of minor families are unique. **(C)** Distribution of biosynthetic gene cluster (BGC) product types across bacterial phyla. Each bar represents the total number of BGCs assigned to a given phylum, coloured by predicted product type. The striped lines indicate the proportion of BGCs that were classified as "on contig edge" by antiSMASH. Phyla are ordered by total BGC count, with "Unknown" indicating contigs for which no confident phylum-level classification could be assigned.

## Data availability

The datasets generated during and/or analysed during the current study are available in the ENA repository, `https://www.ebi.ac.uk/ena/browser/view/PRJEB89893`. The extracted MAGs and identified biosynthetic gene clusters (BGCs) are available at Zenodo (DOI: 10.5281/zenodo.15533781 for the total metagenome assembly and DOI: 10.5281/zenodo.15529477 for MAGs) and in the BGC Atlas repos-

itory at https://bgc-atlas.cs.uni-tuebingen.de/ultra-deep-soil/.

## Additional Files

*Supplementary Fig. S1.* 17-mer spectrum of Illumina reads.

*Supplementary Fig. S2.* 17-mer spectrum of Nanopore reads

*Supplementary Fig. S3.* The number of occurences of each marker gene from bac120 set in raw Nanopore reads.

*Supplementary Table S1.* The details of the extracted MAGs, including their completeness and contamination values, taxonomic classification, assembly statistics, genome annotations statistics, and the number of BGCs identified.

## Declarations

### List of abbreviations

ANI: average nucleotide identity; BGC: biosynthetic gene cluster; bp: base pairs; CPR: Candidate phyla radiation; DNA: deoxyribonucleic acid; ENA: European Nucleotide Archive; Gbp: giga (billion) base pairs; GCF: gene cluster family; GTDB: Genome Taxonomy Database; HQ: high quality; MAG: metagenome-assembled genome; MIBIG: Minimum Information about a Biosynthetic Gene Cluster; MIMAG: Minimum Information about a Metagenome-Assembled Genome; MQ: medium quality; NGS: next-generation sequencing; NRPS: non-ribosomal peptide synthetase; kbp: kilo (thousand) base pairs; Mbp: mega (million) base pairs; ONT: Oxford Nanopore Technologies; OTU: operational taxonomic unit, PKS: polyketide synthase; Q-score: quality score (Phred); RiPP: ribosomally synthesised and post-transcriptionally modified peptide

### Ethical Approval

Not applicable

### Consent for publication

Not applicable

### Competing Interests

The authors declare that they have no competing interests'.

### Funding

This work was supported by Bundesministerium für Bildung und Forschung (BMBF) [161L0284C] to N.Z., Deutsches Zentrum für Infektionsforschung (DZIF) [TTU09.716] to N.Z., and Deutsche Forschungsgemeinschaft (DFG) [INST 37/1049-1] to S.O..

### Author's Contributions

C.B. and N.Z. wrote the main manuscript. C.B. performed the data analysis and prepared the figures. T.N. conducted the soil sampling and DNA isolation. E.A.B. carried out the Nanopore sequencing. C.G. performed basecalling and preliminary quality control of the sequencing data. C.B., S.O., and N.Z. conceptualised the study. All authors reviewed the manuscript.

## Acknowledgements

The authors acknowledge the support by the High Performance and Cloud Computing Group at the Zentrum für Datenverarbeitung of the University of Tübingen and the Federal Ministry of Education and Research (BMBF) through grant no. 031 A535A. We thank the Interfaculty Institute for Biomedical Informatics (IBMI) at the University of Tübingen for providing the computational resources essential for this study. NGS sequencing methods were performed with the support of the DFG-funded NGS Competence Center Tübingen (INST 37/1049-1). Additionally, we thank the Deutsche Forschungsgemeinschaft (DFG, German Research Foundation) under Germany's Excellence Strategy—EXC 2124—390838134 for structural support.

## References

1. Fierer N. Embracing the unknown: disentangling the complexities of the soil microbiome. Nature Reviews Microbiology 2017;15(10):579–590.
2. Roesch LF, Fulthorpe RR, Riva A, Casella G, Hadwin AK, Kent AD, et al. Pyrosequencing enumerates and contrasts soil microbial diversity. The ISME journal 2007;1(4):283–290.
3. Torsvik V, Øvreås L. Microbial diversity and function in soil: from genes to ecosystems. Current opinion in microbiology 2002;5(3):240–245.
4. Jousset A, Bienhold C, Chatzinotas A, Gallien L, Gobet A, Kurm V, et al. Where less may be more: how the rare biosphere pulls ecosystems strings. The ISME journal 2017;11(4):853–862.
5. Lok C. Mining the microbial dark matter. Nature 2015;522(7556):270.
6. Hug LA, Baker BJ, Anantharaman K, Brown CT, Probst AJ, Castelle CJ, et al. A new view of the tree of life. Nature microbiology 2016;1(5):1–6.
7. Lloyd KG, Steen AD, Ladau J, Yin J, Crosby L. Phylogenetically novel uncultured microbial cells dominate earth microbiomes. MSystems 2018;3(5):10–1128.
8. Nascimento Lemos L, Manoharan L, William Mendes L, Monteiro Venturini A, Satler Pylro V, Tsai SM. Metagenome assembled-genomes reveal similar functional profiles of CPR/Patescibacteria phyla in soils. Environmental microbiology reports 2020;12(6):651–655.
9. Nayfach S, Roux S, Seshadri R, Udwary D, Varghese N, Schulz F, et al. A genomic catalog of Earth's microbiomes. Nature biotechnology 2021;39(4):499–509.
10. Parks DH, Rinke C, Chuvochina M, Chaumeil PA, Woodcroft BJ, Evans PN, et al. Recovery of nearly 8,000 metagenome-assembled genomes substantially expands the tree of life. Nature microbiology 2017;2(11):1533–1542.
11. Bertrand D, Shaw J, Kalathiyappan M, Ng AHQ, Kumar MS, Li C, et al. Hybrid metagenomic assembly enables high-resolution analysis of resistance determinants and mobile elements in human microbiomes. Nature biotechnology 2019;37(8):937–944.
12. Van Goethem MW, Osborn AR, Bowen BP, Andeer PF, Swenson TL, Clum A, et al. Long-read metagenomics of soil communities reveals phylum-specific secondary metabolite dynamics. Communications biology 2021;4(1):1302.
13. Agustinho DP, Fu Y, Menon VK, Metcalf GA, Treangen TJ, Sedlazeck FJ. Unveiling microbial diversity: harnessing long-read sequencing technology. Nature methods 2024;21(6):954–966.
14. Wick RR, Judd LM, Holt KE. Assembling the perfect bacterial genome using Oxford Nanopore and Illumina sequencing. PLOS Computational Biology 2023;19(3):e1010905.
15. Zhang T, Li H, Jiang M, Hou H, Gao Y, Li Y, et al. Nanopore sequencing: Flourishing in its teenage years. Journal of Genetics and Genomics 2024;.

16. Mantri SS, Negri T, Sales-Ortells H, Angelov A, Peter S, Neidhardt H, et al. Metagenomic sequencing of multiple soil horizons and sites in close vicinity revealed novel secondary metabolite diversity. Msystems 2021;6(5):10–1128.
17. Negri T, Mantri S, Angelov A, Peter S, Muth G, Eustáquio AS, et al. A rapid and efficient strategy to identify and recover biosynthetic gene clusters from soil metagenomes. Applied Microbiology and Biotechnology 2022;106(8):3293–3306.
18. Chen S. Ultrafast one-pass FASTQ data preprocessing, quality control, and deduplication using fastp. Imeta 2023;2(2):e107.
19. Kolmogorov M, Bickhart DM, Behsaz B, Gurevich A, Rayko M, Shin SB, et al. metaFlye: scalable long-read metagenome assembly using repeat graphs. Nature methods 2020;17(11):1103–1110.
20. Oxford Nanopore Technologies, Medaka: sequence correction provided by ONT Research; 2023. Accessed: 2025-05-26. https://github.com/nanoporetech/medaka.
21. Hu J, Fan J, Sun Z, Liu S. NextPolish: a fast and efficient genome polishing tool for long-read assembly. Bioinformatics 2020;36(7):2253–2255.
22. Li H, Durbin R. Fast and accurate short read alignment with Burrows–Wheeler transform. bioinformatics 2009;25(14):1754–1760.
23. Li H. Minimap2: pairwise alignment for nucleotide sequences. Bioinformatics 2018;34(18):3094–3100.
24. Li D, Liu CM, Luo R, Sadakane K, Lam TW. MEGAHIT: an ultra-fast single-node solution for large and complex metagenomics assembly via succinct de Bruijn graph. Bioinformatics 2015;31(10):1674–1676.
25. Kim J, Steinegger M. Metabuli: sensitive and specific metagenomic classification via joint analysis of amino acid and DNA. Nature Methods 2024;21(6):971–973.
26. Parks DH, Chuvochina M, Rinke C, Mussig AJ, Chaumeil PA, Hugenholtz P. GTDB: an ongoing census of bacterial and archaeal diversity through a phylogenetically consistent, rank normalized and complete genome-based taxonomy. Nucleic acids research 2022;50(D1):D785–D794.
27. Rhie A, Nurk S, Cechova M, Hoyt SJ, Taylor DJ, Altemose N, et al. The complete sequence of a human Y chromosome. Nature 2023;621(7978):344–354.
28. Steinegger M, Söding J. MMseqs2 enables sensitive protein sequence searching for the analysis of massive data sets. Nature biotechnology 2017;35(11):1026–1028.
29. Wang Z, You R, Han H, Liu W, Sun F, Zhu S. Effective binning of metagenomic contigs using contrastive multi-view representation learning. Nature Communications 2024;15(1):585.
30. Liu CC, Dong SS, Chen JB, Wang C, Ning P, Guo Y, et al. MetaDecoder: a novel method for clustering metagenomic contigs. Microbiome 2022;10(1):46.
31. Pan S, Zhao XM, Coelho LP. SemiBin2: self-supervised contrastive learning leads to better MAGs for short-and long-read sequencing. Bioinformatics 2023;39(Supplement_1):i21–i29.
32. Nissen JN, Johansen J, Allesøe RL, Sønderby CK, Armenteros JJA, Grønbech CH, et al. Improved metagenome binning and assembly using deep variational autoencoders. Nature biotechnology 2021;39(5):555–560.
33. Parks DH, Imelfort M, Skennerton CT, Hugenholtz P, Tyson GW. CheckM: assessing the quality of microbial genomes recovered from isolates, single cells, and metagenomes. Genome research 2015;25(7):1043–1055.
34. Hyatt D, Chen GL, LoCascio PF, Land ML, Larimer FW, Hauser LJ. Prodigal: prokaryotic gene recognition and translation initiation site identification. BMC bioinformatics 2010;11:1–11.
35. Sahlin K. Strobealign: flexible seed size enables ultra-fast and accurate read alignment. Genome Biology 2022;23(1):260.
36. Kutuzova S, Piera P, Nor Nielsen K, Olsen NS, Riber L, Gobbi A, et al. Binning meets taxonomy: TaxVAMB improves metagenome binning using bi-modal variational autoencoder. bioRxiv 2024;p. 2024–10.
37. Rühlemann MC, Wacker EM, Ellinghaus D, Franke A. MAGScoT: a fast, lightweight and accurate bin-refinement tool. Bioinformatics 2022;38(24):5430–5433.
38. Eddy SR. Accelerated profile HMM searches. PLoS computational biology 2011;7(10):e1002195.
39. Mistry J, Chuguransky S, Williams L, Qureshi M, Salazar GA, Sonnhammer EL, et al. Pfam: The protein families database in 2021. Nucleic acids research 2021;49(D1):D412–D419.
40. Haft DH, Selengut JD, Richter RA, Harkins D, Basu MK, Beck E. TIGRFAMs and genome properties in 2013. Nucleic acids research 2012;41(D1):D387–D395.
41. Chklovski A, Parks DH, Woodcroft BJ, Tyson GW. CheckM2: a rapid, scalable and accurate tool for assessing microbial genome quality using machine learning. Nature methods 2023;20(8):1203–1212.
42. Ondov BD, Treangen TJ, Melsted P, Mallonee AB, Bergman NH, Koren S, et al. Mash: fast genome and metagenome distance estimation using MinHash. Genome biology 2016;17:1–14.
43. Chaumeil PA, Mussig AJ, Hugenholtz P, Parks DH. GTDB-Tk v2: memory friendly classification with the genome taxonomy database. Bioinformatics 2022;38(23):5315–5316.
44. Letunic I, Bork P. Interactive Tree of Life (iTOL) v6: recent updates to the phylogenetic tree display and annotation tool. Nucleic acids research 2024;52(W1):W78–W82.
45. Mohamadi H, Khan H, Birol I. ntCard: a streaming algorithm for cardinality estimation in genomics data. Bioinformatics 2017;33(9):1324–1330.
46. Rodriguez-R LM, Gunturu S, Tiedje JM, Cole JR, Konstantinidis KT. Nonpareil 3: fast estimation of metagenomic coverage and sequence diversity. MSystems 2018;3(3):10–1128.
47. Seeman T, Barrnap 0.9: rapid ribosomal RNA prediction; 2013. https://github.com/tseemann/barrnap.
48. Rognes T, Flouri T, Nichols B, Quince C, Mahé F. VSEARCH: a versatile open source tool for metagenomics. PeerJ 2016;4:e2584.
49. Schwengers O, Jelonek L, Dieckmann MA, Beyvers S, Blom J, Goesmann A. Bakta: rapid and standardized annotation of bacterial genomes via alignment-free sequence identification. Microbial genomics 2021;7(11):000685.
50. Blin K, Shaw S, Augustijn HE, Reitz ZL, Biermann F, Alanjary M, et al. antiSMASH 7.0: new and improved predictions for detection, regulation, chemical structures and visualisation. Nucleic acids research 2023;51(W1):W46–W50.
51. Navarro-Muñoz JC, Selem-Mojica N, Mullowney MW, Kautsar SA, Tryon JH, Parkinson EI, et al. A computational framework to explore large-scale biosynthetic diversity. Nature chemical biology 2020;16(1):60–68.
52. Zdouc MM, Blin K, Louwen NL, Navarro J, Loureiro C, Bader CD, et al. MIBiG 4.0: advancing biosynthetic gene cluster curation through global collaboration. Nucleic acids research 2025;53(D1):D678–D690.
53. Bağcı C, Nuhamunada M, Goyat H, Ladanyi C, Sehnal L, Blin K, et al. BGC Atlas: a web resource for exploring the global chemical diversity encoded in bacterial genomes. Nucleic acids research 2025;53(D1):D618–D624.
54. Bowers RM, Kyrpides NC, Stepanauskas R, Harmon-Smith M, Doud D, Reddy T, et al. Minimum information about a single amplified genome (MISAG) and a metagenome-assembled genome (MIMAG) of bacteria and archaea. Nature biotechnology 2017;35(8):725–731.
55. Blanco-Míguez A, Beghini F, Cumbo F, McIver LJ, Thompson KN, Zolfo M, et al. Extending and improving metagenomic taxonomic profiling with uncharacterized species using MetaPhlAn 4. Nature Biotechnology 2023;41(11):1633–1644.
56. Chen J, Jia Y, Sun Y, Liu K, Zhou C, Liu C, et al. Global marine microbial diversity and its potential in bioprospecting. Nature 2024;633(8029):371–379.

57. Pasolli E, Asnicar F, Manara S, Zolfo M, Karcher N, Armanini F, et al. Extensive unexplored human microbiome diversity revealed by over 150,000 genomes from metagenomes spanning age, geography, and lifestyle. Cell 2019;176(3):649–662.
58. Sunagawa S, Coelho LP, Chaffron S, Kultima JR, Labadie K, Salazar G, et al. Structure and function of the global ocean microbiome. Science 2015;348(6237):1261359.
59. Kim CY, Lee M, Yang S, Kim K, Yong D, Kim HR, et al. Human reference gut microbiome catalog including newly assembled genomes from under-represented Asian metagenomes. Genome Medicine 2021;13:1–20.
60. Ni J, Yan Q, Yu Y. How much metagenomic sequencing is enough to achieve a given goal? Scientific reports 2013;3(1):1968.
61. Duarte CM, Ngugi DK, Alam I, Pearman J, Kamau A, Eguiluz VM, et al. Sequencing effort dictates gene discovery in marine microbial metagenomes. Environmental Microbiology 2020;22(11):4589–4603.
62. Nishimura Y, Yoshizawa S. The OceanDNA MAG catalog contains over 50,000 prokaryotic genomes originated from various marine environments. Scientific Data 2022;9(1):305.
63. Richardson L, Allen B, Baldi G, Beracochea M, Bileschi ML, Burdett T, et al. MGnify: the microbiome sequence data analysis resource in 2023. Nucleic acids research 2023;51(D1):D753–D759.
64. Gurbich TA, Almeida A, Beracochea M, Burdett T, Burgin J, Cochrane G, et al. MGnify genomes: a resource for biome-specific microbial genome catalogues. Journal of Molecular Biology 2023;435(14):168016.
65. Udwary DW, Doering DT, Foster B, Smirnova T, Kautsar SA, Mouncey NJ. The secondary metabolism collaboratory: a database and web discussion portal for secondary metabolite biosynthetic gene clusters. Nucleic Acids Research 2025;53(D1):D717–D723.
66. Larralde M, Zeller G. Machine learning inference of natural product chemistry across biosynthetic gene cluster types. bioRxiv 2025;p. 2025–03.
67. Skinnider MA, Johnston CW, Gunabalasingam M, Merwin NJ, Kieliszek AM, MacLellan RJ, et al. Comprehensive prediction of secondary metabolite structure and biological activity from microbial genome sequences. Nature communications 2020;11(1):6058.
68. Li Z, Riley WJ, Marschmann GL, Karaoz U, Shirley IA, Wu Q, et al. A framework for integrating genomics, microbial traits, and ecosystem biogeochemistry. Nature Communications 2025;16(1):2186.

Click here to access/download
**Supplementary Material**
Supplementary_Figures.pdf

Click here to access/download
**Supplementary Material**
Supplementary Table S1.xlsx

Personal Cover
Click here to access/download;Personal Cover;CoverLetter.docx

EBERHARD KARLS
UNIVERSITÄT
TÜBINGEN

**Angewandte Naturstoffgenomik**

**Universität Tübingen ·** Prof. Dr. Nadine Ziemert ·
Interfakultäres Institut für Mikrobiologie und Infektionsmedizin Tübingen (IMIT)
Angewandte Naturstoffgenomik · Auf der Morgenstelle 24 · 72076 Tübingen

**Prof. Dr. Nadine Ziemert**

Auf der Morgenstelle 24
72076 Tübingen
Telefon +49 7071 29-78841
Telefax +49 7071 29-5979
Nadine.ziemert @uni-tuebingen.de

**Scott Edmunds, PhD**
**Editor-in-Chief**
***GigaScience***

3. July 2025

Dear Editors,

We are pleased to submit our manuscript entitled "Ultra-deep long-read metagenomics captures diverse taxonomic and biosynthetic potential of soil microbes" for consideration in GigaScience.

This study presents one of the deepest metagenomic sequencing efforts to date on a single soil sample, combining 148 Gbp of Oxford Nanopore long-read data with 122 Gbp of Illumina short reads. Our hybrid assembly strategy enabled the recovery of 837 metagenome-assembled genomes (MAGs), including 466 high- and medium-quality genomes, nearly all of which represent previously uncharacterized taxa.

Using a combination of k-mer, 16S rRNA, and single-copy marker gene analysis, our rarefaction analysis demonstrates that even at this exceptional sequencing depth, soil microbial diversity remains unsaturated. These findings provide a critical empirical benchmark for future studies aiming to quantify microbial complexity in environmental samples. In addition, we identified over 11,000 biosynthetic gene clusters (BGCs), the vast majority of which have no close matches in current reference databases, underscoring the largely untapped biosynthetic potential of soil microbiomes.

We believe this work is well aligned with GigaScience's mission to publish data-rich studies and promote transparency and reproducibility in life science research. The preprint version of this manuscript (https://doi.org/10.1101/2025.05.28.656579) has already attracted significant attention in the community, reflecting strong interest in efforts to quantify microbial and biosynthetic diversity in environmental samples at ultra-deep resolution.

The raw sequencing data and assembled genomes have already been deposited and made publicly available in the European Nucleotide Archive (ENA). If the manuscript is considered, we will make all associated analysis results, including biosynthetic gene cluster annotations and detailed analysis of the MAGs, publicly available via GigaDB.

All authors have approved the manuscript and declare no competing interests. This work is not under consideration elsewhere.

Thank you for considering our submission. We look forward to your feedback.

Sincerely,

**Interfakultäres Institut für Mikrobiologie und Infektionsmedizin (IMIT)**
Translational Genome Mining · Auf der Morgenstelle 28 · 72076 Tübingen
Telefon +49 7071 29-78840 · Telefax +49 7071 29-5979
Nadine.ziemert@uni-tuebingen.de

Prof. Dr. Nadine Ziemert
