# Supplementary material for: Ultra-deep long-read metagenomics captures diverse taxonomic and biosynthetic potential of soil microbes
Source: Gigascience. 2025 Oct 24;14:giaf135. doi: 10.1093/gigascience/giaf135 (PMC12690461; doi:10.1093/gigascience/giaf135)
Supplement: giaf135_GIGA-D-25-00264_Revision_1 [file giaf135_giga-d-25-00264_revision_1.pdf]

GigaScience

Ultra-deep long-read metagenomics captures diverse taxonomic and biosynthetic potential of soil microbes

--Manuscript Draft--

**Manuscript Number:**	GIGA-D-25-00264R1
**Full Title:**	Ultra-deep long-read metagenomics captures diverse taxonomic and biosynthetic potential of soil microbes
**Article Type:**	Research
**Funding Information:**	Bundesministerium für Bildung und Forschung (161L0284C) — Dr. Nadine Ziemert<br>Deutsches Zentrum für Infektionsforschung (TTU09.716) — Dr. Nadine Ziemert<br>Deutsche Forschungsgemeinschaft (INST 37/1049-1) — Dr. Stephan Ossowski
**Abstract:**	Background: Soil ecosystems have long been recognised as hotspots of microbial diversity, but most estimates of their microbial and functional complexity remain speculative despite decades of study, in part because conventional sequencing campaigns lack the depth and contiguity required to recover low-abundance and repetitive genomes. Here, we revisit this question using one of the deepest metagenomic sequencing efforts to date, applying 148 billion base pairs of Nanopore long-read and 122 billion base pairs of Illumina short-read data to a single forest soil sample.<br>Results: Our hybrid assembly reconstructed 837 metagenome-assembled genomes, including 466 that meet high- and medium-quality standards, nearly all lacking close relatives among cultivated taxa. Rarefaction and k-mer analyses reveal that, even at this depth, we capture only a fraction of the extant diversity: non-parametric models project that more than ten trillion base pairs of sequencing data would be required to approach saturation. These findings offer a quantitative, technology-enabled update to long-standing diversity estimates and demonstrate that conventional metagenomic sequencing efforts likely miss the majority of microbial and biosynthetic potential in soil. We further identify more than 11,000 biosynthetic gene clusters, over 99% of which have no match in current databases, underscoring the breadth of unexplored metabolic capacity.<br>Conclusions: Taken together, our results emphasise both the power and the present limitations of metagenomics in resolving natural microbial complexity, and they provide a new baseline for evaluating future advances in microbial genome recovery, taxonomic classification, and natural product discovery.
**Corresponding Author:**	Nadine Ziemert<br>Universitätsklinikum Tübingen: Universitatsklinikum Tubingen<br>Tuebingen, GERMANY
**Corresponding Author Secondary Information:**	
**Corresponding Author's Institution:**	Universitätsklinikum Tübingen: Universitatsklinikum Tubingen
**Corresponding Author's Secondary Institution:**	
**First Author:**	Caner Bağcı, Dr.
**First Author Secondary Information:**	
**Order of Authors:**	Caner Bağcı, Dr.<br>Timo Negri, Dr.<br>Elena Buena Atienza, Dr.<br>Caspar Gross, MSc.

Powered by Editorial Manager® and ProduXion Manager® from Aries Systems Corporation

	Stephan Ossowski, Prof. Dr.
	Nadine Ziemert, Prof. Dr.
**Order of Authors Secondary Information:**	
**Response to Reviewers:**	Dear Editor,<br><br>We thank you and the reviewers for the constructive and insightful feedback, which has substantially improved our manuscript. Below we provide a point-by-point response to each comment. Reviewers' comments are shown in italics, followed by our responses. We would like note that we have also addressed the editorial comment and made our computational workflow available on GitHub and deposited it on workflowhub.eu.<br><br>Reviewer #1: Using the deepest soil metagenomic sequencing to date, this study reconstructed 837 MAGs and over 11,000 BGCs. The results show that even with such deep sequencing, only a fraction of the soil microbial diversity was captured, highlighting the contribution of ultra-deep metagenomics to microbial studies and vast unknown microbial diversity and functional potentials. Although the authors have provided insights for future research on soil microbes using high-depth sequencing and a novel hybrid assembly strategy, there are several major concerns I should be point out:<br><br>1. A primary concern with the current analysis is the use of all recovered MAGs, regardless of quality. Although 466 of the 837 MAGs met established high- or medium-quality standards (MIMAG criteria), the inclusion of the remaining low-quality MAGs is problematic. Such genomes are highly susceptible to errors, including fragmentation and contamination, which can lead to spurious findings in taxonomic assignments and the assessment of biosynthetic potential. To enhance the rigor and validity of the study, it is recommended to remove the low-quality MAGs before proceeding with further analysis.<br><br>All analyses in our original submission were already based on either the full-assembly (taxonomic novelty of the contigs, for example) or restricted to MAGs that met high- or medium-quality MIMAG standards (466 of 837 bins). We have now made this filtering step more explicit in the Methods and Results to avoid any possible confusion. Figure 5 (previously Figure 1) and all associated analyses continue to reflect only these high- and medium-quality MAGs.<br><br>2. The study yielded 10.5 GB of co-assembled contigs with an N50 of 77.8 kb. However, the analysis appears to have only predicted BGCs from recovered MAGs. This approach overlooks the vast majority of the high-quality assembly data and likely underestimates the total biosynthetic potential. We recommend a more comprehensive analysis by predicting BGCs directly from the entire co-assembled contigs. This would likely yield a greater and more diverse results, providing a more complete picture of the community's metabolic capabilities.<br><br>We apologise for the lack of clarity. We clarify in the revised manuscript that BGC prediction was performed on the entire hybrid co-assembly, not only on MAGs. We have restructured the Results section to make this explicit and avoid confusion.<br><br>3. Normally, 16S community analysis is considered the gold standard for studying microbial diversity. Here, the metagenomic results from different sequencing depths could be compared with the 16S diversity analysis results to see what proportion of the diversity can actually be recovered by metagenomic sequencing depth.<br><br>We respectfully disagree that amplicon sequencing would be appropriate in this context. 16S rRNA amplicon surveys have well-documented limitations, including primer bias, inability to resolve strain-level diversity, and systematic underestimation of richness in highly complex environments such as soil. Our goal was to use metagenomic data to provide a genome-resolved, quantitative benchmark of diversity; adding a parallel amplicon study would not improve this resolution and might even mislead by suggesting lower diversity than truly exists.<br><br>Some minor comments:

Powered by Editorial Manager® and ProduXion Manager® from Aries Systems Corporation

4. At least three versions of GTDB database were used?

We now clarify in the Methods which GTDB versions were used by which tools: v214.1 for taxonomic assignments (MMseqs2, Metabuli), v95 for SemiBin2 (pretrained model), and r220 for GTDB-Tk classification. At the time of analysis these were the most recent versions compatible with each tool.

5. In "Rarefaction analysis" part, how many 16S rRNA sequences were extracted, and how many OTUs were clustered?

We now provide these numbers on Figure 3 and provide a supplementary Figure on the size distribution of the extracted 16S rRNA seqeunces. We have also removed the term “OTU” to avoid implying a taxonomic rank, and we frame these as “clustered 16S rRNA gene sequences.”

6. The method and criteria to assess novel BGCs comparing against the MIBiG 4.0 and BGC Atlas databases

We added a detailed description of novelty assessment in the Methods, including the BiG-SCAPE and BiG-SLiCE distance thresholds used and cross-checking against MIBiG 4.0 and BGC Atlas.

7. First part of the results section seems to overlap with the methods section

We retained a brief description of key steps in the Results to aid readers who jump directly to this section. This summary is concise and limited to the minimum needed for readability.

8. Figure 1A and 2B can be arranged in one line.

The figure numbering has changed: the content that was previously Figure 1 is now part of Figure 5 (MAG novelty). If Figure 2A and 2B are meant, the relative abundances for taxonomic assignments and novelty are more interpretable when presented separately. We believe that combining them would compromise readability but are happy to follow the journal’s typesetting team’s recommendation if they prefer to merge.

9. In figure4, the rarefaction lines for 100 and 97 cluster level are totally coincident. It is quite intriguing. This makes me wonder if the Nanopore data was not corrected during the 16S sequence prediction. Nanopore data has a high error rate, and using the raw data directly without correction would lead to significant errors.

We appreciate this comment and have clarified our approach in the revised manuscript. The 97% and 100% clustering curves are indeed very close but not identical. The slight convergence likely reflects a combination of true strain-level diversity and the inherent error rate of Nanopore reads. Error correction is not feasible at this depth because most 16S sequences originate from low-abundance taxa with limited coverage. Nevertheless, we validated the extracted sequences by aligning them to the Illumina-only assembly. There were only 176 full-length 16S rRNA genes detected in the short-read assembly, due to its fragmented nature. Of these 176, 12 match to the Nanopore reads with >99.9% accuracy, 16 with > 99.0% accuracy, and 79 with > 97% accuracy (Data not shown). Considering some might be missing in full length in the Nanopore sequencing, more than half of the 16S rRNAs predicted from Nanopore reads should still have above 97% accuracy.

Reviewer #2: By applying ultra-deep hybrid metagenomic sequencing (270 Gbp total; 148 Gbp Nanopore + 122 Gbp Illumina) to a single Cambisol forest soil sample, the authors achieve near-unprecedented recovery of soil microbial diversity and biosynthetic potential. The work demonstrates the vast, untapped microbial and genetic resource content in soils—particularly in terms of biosynthetic gene clusters (BGCs) and uncultivated microbial lineages. Furthermore, the study underscores the necessity of employing longer and deeper sequencing strategies in order to access the full extent of the soil microbial "dark matter." However, the following points are raised

for the authors' consideration:

1. The study is based on a single soil sample, which limits ecological generalizability and the interpretability of the dataset. A brief discussion of how representative this sample might be would help contextualize the findings.

We agree and have added a statement to the Discussion clarifying that this study represents a depth benchmark for a representative Cambisol soil rather than a global census. We note that the heavy-tailed diversity distribution is expected to hold for other complex soils, but taxonomic composition may vary with soil type, season, and land use.

2. The authors state that "Full-length 16S rRNA gene sequences were extracted from Nanopore reads using Barrnap [47] (v0.9)." However, it is unclear how the authors define or validate that the extracted 16S rRNA sequences are indeed "full-length." In typical metagenomic datasets—especially from complex environments like soil—16S rRNA gene sequences are often fragmented due to challenges in assembly, repetitive regions, and read truncation. I recommend the authors provide a length distribution summary of the extracted 16S rRNA gene sequences. This information is critical for assessing the completeness and taxonomic resolution of 16S-based analyses.

We now provide the length distribution of extracted 16S rRNA sequences in the Supplementary data and specify that only sequences flagged as “partial” by Barrnap were excluded.

3. In Figure 2B, the authors present the taxonomic novelty of assembled contigs, with bar plots indicating the proportion of sequences assigned to cultured taxa (blue), uncultured taxa (green), and completely unclassified taxa (orange). However, it appears that the proportion of uncultured taxa—those detected in other metagenomic datasets but lacking cultured representatives—is surprisingly low at certain taxonomic levels (e.g., only ~5% at the species level were uncultured). Given the known high proportion of uncultured lineages in soil microbiomes, this result seems unexpected. Could the authors clarify how these proportions were calculated? Specifically:

* What database and criteria were used to distinguish between "uncultured" vs. "unclassified"?
* Were contigs with ambiguous or low-confidence taxonomic assignments excluded or grouped into "unclassified"?
A more detailed explanation—perhaps in the figure legend or Supplementary Methods—would help readers better interpret the novelty metrics presented in this important figure.

We expanded the Methods and figure legends to explain the database sources (GTDB metadata), the criteria for “cultured,” “uncultured,” and “unclassified,” and how ambiguous assignments were handled.

4. The manuscript states that >99% of BGCs are novel. While this is likely true given soil diversity, it would be helpful to describe the criteria used to define "novelty" (e.g., BiG-SCAPE clustering distance to known MIBiG entries). A summary of the major BGC types (NRPS, PKS, RiPPs, terpenes, etc.) and their taxonomic affiliations would also enhance interpretability.

As noted above, we added the BiG-SCAPE and BiG-SLiCE distance thresholds to the Methods. Figure 4C summarises BGC product types by phylum, providing a clearer view of the taxonomic distribution of biosynthetic capacity.

5. The manuscript's analytical structure raises some concerns. The flow of results appears somewhat fragmented: the authors begin by analyzing MAGs, then shift to contig-based taxonomic novelty, followed by sequencing depth diagnostics (e.g., rarefaction of 16S sequences), and finally return to MAG-based BGC analysis. This sequence may confuse readers and obscure the relationship between the different analytical layers. A clearer explanation of the rationale for this order, or a reorganization to reflect the analytical logic (e.g., assembly → gene catalog → MAGs → functional potential), could improve the manuscript's coherence.

Powered by Editorial Manager® and ProduXion Manager® from Aries Systems Corporation

	We agree that the flow of the presentation of results in the initial submission was difficult to follow, and have reorganized the Results to follow a more intuitive flow: assembly → taxonomic classification → diversity analyses → functional potential (BGCs) → MAGs. We believe this greatly improves readability.<br><br>6. The manuscript would benefit from a more integrated functional perspective. While the BGC analysis is comprehensive, there is little discussion of non-BGC functional annotation—either at the contig level (gene catalog) or the MAG level. Functional profiling using KEGG, eggNOG, or CAZy annotations could provide broader insights into the ecological roles of the recovered genomes and improve the interpretability of the massive sequencing effort.<br><br>We performed this analysis and made the data available on zenodo for the total assembly. However, we think discussing this in more detail is beyond the scope of the manuscript. However, this gave us the idea of using coding sequences as another proxy to evaluate the (functional) diversity of the sample. We performed a rarefaction of the functional space and added it to the main manuscript as well.
**Additional Information:**	
**Question**	**Response**
Are you submitting this manuscript to a special series or article collection?	No
**Experimental design and statistics**<br><br>Full details of the experimental design and statistical methods used should be given in the Methods section, as detailed in our Minimum Standards Reporting Checklist. Information essential to interpreting the data presented should be made available in the figure legends.<br><br>Have you included all the information requested in your manuscript?	Yes
**Resources**<br><br>A description of all resources used, including antibodies, cell lines, animals and software tools, with enough information to allow them to be uniquely identified, should be included in the Methods section. Authors are strongly encouraged to cite Research Resource Identifiers (RRIDs) for antibodies, model organisms and tools, where possible.<br><br>Have you included the information requested as detailed in our Minimum	Yes

Powered by Editorial Manager® and ProduXion Manager® from Aries Systems Corporation

Standards Reporting Checklist?	
**Availability of data and materials**<br><br>All datasets and code on which the conclusions of the paper rely must be either included in your submission or deposited in publicly available repositories (where available and ethically appropriate), referencing such data using a unique identifier in the references and in the "Availability of Data and Materials" section of your manuscript.<br><br>Have you have met the above requirement as detailed in our Minimum Standards Reporting Checklist?	Yes
GigaScience has policies and guidelines in place for the use of generative AI-writing tools such as ChatGPT. If you have used such writing tools to assist with writing the manuscript this must be declared and cited in the text. Authors should not list AI-writing tools and other AI-assisted technologies as an author or co-author and should acknowledge that they are fully responsible for text generated or refined by AI-writing tools.<p><br><br>A summary of use (particularly in the introduction or among methods) needs to be included at the end of the paper, and the outputs should also be included as a supplementary file hosted in GigaDB or other open repositories. Please read our guidelines for more information. <p><br><br>By submitting to GigaScience, you are aware of the journal's AI-writing tools policy, and if you have declared use of such tools below, you have acknowledged this where appropriate in your manuscript and have made a summary of use and outputs available. <p><br><b>AI-assisted writing tools have been used in the preparation of this manuscript?	No

LaTeX - Main Document (TeX file) Click here to access/download;LaTeX - Main Document (TeX file);main.tex

Placeholder for OUP logo oup.pdf

Placeholder for journal logo gigascience-logo.pdf

GigaScience, 2025, 1–14

doi: xx.xxxx/xxxx
Manuscript in Preparation
Research

RESEARCH

Ultra-deep long-read metagenomics captures diverse taxonomic and biosynthetic potential of soil microbes

Caner Bağcı [0000-0003-4428-9515][1,2,3], Timo Negri[1,2,3], Elena Buena Atienza [0000-0002-9890-1960][4,5], Caspar Gross [0000-0002-9009-5458][4,5], Stephan Ossowski [0000-0002-7416-9568][4,5] and Nadine Ziemert [0000-0002-7264-1857][1,2,3,*]

[1]Translational Genome Mining for Natural Products, Interfaculty Institute of Microbiology and Infection Medicine Tübingen (IMIT), University of Tübingen, Auf der Morgenstelle 24, 72076, Tübingen, Baden-Württemberg, Germany and [2]Interfaculty Institute for Biomedical Informatics (IBMI), University of Tübingen, Auf der Morgenstelle 24, 72076, Tübingen, Baden-Württemberg, Germany and [3]German Center for Infection Research (DZIF), Partner Site Tübingen, Auf der Morgenstelle 24, 72076, Tübingen, Baden-Württemberg, Germany and [4]Institute of Medical Genetics and Applied Genomics, University of Tübingen, Calwerstrasse 7, 72076, Tübingen, Baden-Württemberg, Germany and [5]NGS Competence Center, University of Tübingen, Calwerstrasse 7, 72076, Tübingen, Baden-Württemberg, Germany

[*]nadine.ziemert@uni-tuebingen.de

Abstract

Background: Soil ecosystems have long been recognised as hotspots of microbial diversity, but most estimates of their microbial and functional complexity remain speculative despite decades of study, in part because conventional sequencing campaigns lack the depth and contiguity required to recover low-abundance and repetitive genomes. Here, we revisit this question using one of the deepest metagenomic sequencing efforts to date, applying 148 billion base pairs of Nanopore long-read and 122 billion base pairs of Illumina short-read data to a single forest soil sample.
Results: Our hybrid assembly reconstructed 837 metagenome-assembled genomes, including 466 that meet high- and medium-quality standards, nearly all lacking close relatives among cultivated taxa. Rarefaction and k-mer analyses reveal that, even at this depth, we capture only a fraction of the extant diversity: non-parametric models project that more than ten trillion base pairs of sequencing data would be required to approach saturation. These findings offer a quantitative, technology-enabled update to long-standing diversity estimates and demonstrate that conventional metagenomic sequencing efforts likely miss the majority of microbial and biosynthetic potential in soil. We further identify more than 11 000 biosynthetic gene clusters, over 99% of which have no match in current databases, underscoring the breadth of unexplored metabolic capacity.
Conclusions: Taken together, our results emphasise both the power and the present limitations of metagenomics in resolving natural microbial complexity, and they provide a new baseline for evaluating future advances in microbial genome recovery, taxonomic classification, and natural product discovery.

Key words: microbiome; soil; metagenomics; nanopore; ultra-deep; long-read sequencing; diversity; natural products; (meta)genome mining

Compiled on: October 22, 2025.
Draft manuscript prepared by the author.

Key Points

- Ultra-deep hybrid sequencing (148 Gbp Nanopore + 122 Gbp Illumina) of a single forest soil sample yielded 837 metagenome-assembled genomes, all lacking cultured counterparts.
- Despite this unprecedented 270 Gbp depth, rarefaction and coverage modelling indicate that more than 10 Tbp of data would still be needed to reach saturation in soil.
- Only 0.7% and 16.7% of all assembled contigs can be assigned to a species and genus, respectively, with at least one cultured representative, highlighting an unprecedented level of novelty in soil.
- The assembly uncovers 11 381 biosynthetic gene clusters forming over 10 000 mostly novel families, spotlighting an immense, untapped reservoir of microbial natural-product potential.

Data Description

In order to quantify how much taxonomic and biosynthetic novelty present-day sequencing can recover from soil, we extracted high-molecular-weight DNA from a single Cambisol forest soil sample (Schönbuch, Germany) and generated an ultra-deep 270 Gbp dataset - 148 Gbp of Oxford Nanopore PromethION long reads (read-length N50 = 12.2 kb) plus 122 Gbp of Illumina NovaSeq reads. The hybrid metagenome assembly we conducted produced a 10.5 Gbp assembly, from which multi-tool binning and subsequent refinement recovered 837 MAGs, and antiSMASH/BiG-SCAPE annotation revealed more than 11 000 largely novel biosynthetic gene clusters, creating a resource for benchmarking assembly or binning pipelines, modelling diversity-coverage relationships and mining natural products; all raw reads are archived under ENA BioProject PRJEB89893 [1], and the polished assembly, MAG set and BGC catalogue are available via Zenodo for unrestricted reuse [2, 3].

Introduction

Soil is one of the most biologically diverse ecosystems on Earth, hosting a massive and largely uncharacterised diversity of microbial life. These microbial communities play critical roles in biogeochemical cycling, soil formation, nutrient turnover, plant health, and climate regulation [4]. Despite their ecological and biotechnological importance, the full extent and functional potential of soil microbiomes remain poorly understood.

A single gram of soil can contain up to 10^9 microbial cells and hundreds of thousands of species, spanning all domains of life [5, 6]. These communities are not only taxonomically complex but also exhibit highly uneven abundance distributions, where rare taxa – collectively termed the "rare biosphere" – may be functionally consequential despite their low abundance [7].

Although the vast diversity of soil microbes has been widely acknowledged for decades, most estimates of their richness are based on indirect approaches, including 16S rRNA gene surveys, limited shotgun datasets, or predictive modelling. As a result, foundational claims about the extent of soil microbial diversity are frequently cited but rarely re-examined with contemporary data. Much of the soil microbiome remains part of the so-called microbial dark matter: lineages with no cultured representatives and no reference genomes [8, 9, 10].

Metagenomics has enabled the cultivation-independent study of microbial communities, allowing for genome-resolved insights into uncultured organisms [11, 12, 13]. However, most metagenomic studies rely on short-read sequencing, which presents challenges in assembling highly complex, strain-rich communities like soil. These limitations hinder the recovery of low-abundance taxa, reduce the completeness of biosynthetic gene clusters, and often lead to fragmented or ambiguous assemblies [12]. Recent advances in long-read sequencing, particularly with Oxford Nanopore Technologies (ONT), have opened new opportunities for soil microbiome research. Long reads can span repeat-rich regions and operons, improving genome recovery and assembly contiguity [14, 15, 16]. Hybrid approaches combining long and short reads can further mitigate the high error rates of long reads while leveraging their structural advantages [17, 18]. Despite these improvements, it remains unclear how far even the most advanced sequencing technologies can go in capturing the full taxonomic and functional diversity of soil.

To establish an empirical reference point for assessing microbial and biosynthetic diversity in soil, we performed one of the deepest metagenomic sequencing efforts to date on a single soil sample, combining 148 billion base pairs (Gbp) of ONT long-read and 122 Gbp of Illumina short-read data. We use this dataset to empirically assess the power and current limitations of metagenomics in resolving complex microbial communities. Specifically, we ask: How much taxonomic and biosynthetic diversity can be recovered from a single soil sample using state-of-the-art sequencing? What proportion of this diversity is represented in existing databases? Can the ultra-deep sequencing approach saturation, or are we still only scratching the surface? By addressing these questions, we aim to provide a data-driven reassessment of microbial diversity in soil and to establish a benchmark for future metagenomic studies of complex ecosystems.

Methods

Sample collection, DNA extraction, and metagenomic sequencing

The A horizon of the soil type Cambisol was sampled from the Schönbuch Forest, near Tübingen, Germany (the same site previously reported by [19]), on 31 May 2022. High molecular weight metagenomic DNA was isolated using a protocol described in detail in our previous studies [19, 20]. Genomic integrity was assessed using pulse-field capillary electrophoresis with the Genomic DNA 165 kb Analysis Kit on a FemtoPulse (Agilent) instrument. Quantitation of DNA was assessed using the dsDNA High Sensitivity Assay on a Qubit 3 fluorometer (Thermo Fisher), and purity was assessed by Nanodrop. A total of 2.4 µg of genomic DNA was used as input for the library preparation with the 1D Ligation Kit SQK-LSK109-XL Sequencing Kit (ONT). Four PromethION R9 flow cells were utilised for long-read Nanopore sequencing, each loaded with 600 ng (50 fmol) of genomic DNA. The raw signal data from the PromethION runs were basecalled with guppy (v 5.0.7) in high-accuracy mode. For complementary short-read sequencing, three NovaSeq 6000 flow cells were loaded with the same isolated DNA; two of them ran with 200 cycles, and one with 300 cycles.

A total of 148 Gbp of Nanopore sequencing data was generated, with a read length N50 of 12.2 kbp. The three Illumina sequencing runs yielded a total of 122 Gbp raw data, with a mean Q-score of 35. The raw Illumina reads were adapter and quality trimmed using fastp [21] (v 0.23.4) with default settings.

Metagenomic assembly

Nanopore reads from all four runs were pooled together and assembled using metaFlye (v2.9.5-b1801) [22] with the `--meta` option optimised for metagenomic data, and the `--nano-raw` option for the error-prone Nanopore reads. The resulting draft assembly was polished with one round of medaka [23] (v 2.0.1) using the `r941_prom_hac_g507` model and the `--bacteria` flag. The medaka polished assembly was further corrected using the trimmed Illumina reads in a single round of NextPolish [24] (v 1.4.1) polishing, with parameters `-max_depth 100` for short-read mapping with bwa [25] (v 0.7.18), and `-min_read_len 1k -max_depth 100 -x map-ont` for long-read mapping with minimap2 [26] (v 2.28-r1209).

In parallel, an Illumina-only assembly was performed using MEGAHIT [27] (v1.2.9) with default metagenomic assembly parameters. All subsequent analyses were based on the hybrid Nanopore-Illumina assembly, which exhibited superior contiguity (contig N50 = 77.8 kbp) and total assembled length (10.5 Gbp).

Taxonomic analysis

Raw reads were taxonomically classified using Metabuli [28] (v 1.0.8), against the precomputed database provided by the authors, which includes GTDB release 214.1 [29] and the human T2T genome [30]. Classification was performed with parameters `--seq-mode 2` for Illumina reads, and `--seq-mode 3` for Nanopore reads. Contigs from the final hybrid assembly were taxonomically classified using MMSeqs2 [31] (v 16.747c6) using the `easy-taxonomy` module, with GTDB release 214.1 [29]. A contig was classified as "cultured" at rank *R*, if its assignment at *R* belongs to a taxon that has at least one genome in GTDB originating from an isolate sequencing; as "uncultured" if an assignment exists at rank *R*, but all representatives of the taxon come from MAG/SAG sequencing efforts; and as "unclassified" if there is no assignment at rank *R* due to insufficient similarity or conflicting similarities. The information on assigned genomes belonging to a cultured or uncultured representative was retrieved from GTDB metadata files.

Rarefaction analysis

Microbial diversity and sequencing coverage were assessed using multiple complementary approaches. K-mer frequency analysis was conducted with ntCard [32] using 15-mers and 17-mers for both Nanopore and Illumina datasets. Nonpareil [33] (v3.3.3) was used to estimate sequence coverage and to project additional sequencing efforts required for complete community recovery using the pooled set of Illumina reads. Full-length 16S rRNA gene sequences were extracted from Nanopore reads using Barrnap [34] (v0.9) (sequences flagged as "partial" were excluded). We performed rarefaction by subsampling them at 10% increments (10-100%), with three independent replicates per depth (distinct random seeds). For each subset, we clustered the subsampled sequences with VSEARCH [35] (v2.15.2), reporting both exact uniques (100% dereplication) and the number of representatives at fixed identity thresholds (e.g. 97%, 94%, 88%, 85%, 70%, 61%). VSEARCH was run with `--strand both --iddef 1 --qmask none` and `--cluster_size` options for maximum sensitivity. In addition, conserved single-copy marker genes from the bac120 set [13] were identified in Nanopore reads using the LAST aligner (v 1615) in frameshift mode (`-F 15`) against the GTDB bac120 reference set, and quantified to independently assess the taxonomic diversity.

Functional annotation, clustering, and biosynthetic gene cluster discovery

The final polished assembly was annotated using Bakta [36] (v 1.11.0, database version 5.1). The identified coding sequences (CDS) were further annotated for their COG categories [37], eggNOG orthologous groups [38], KEGG ontologies, pathways, modules, reactions [39], Gene Ontologies [40], CAZy enzymes [41], and Pfam domains [42], using eggNOG-mapper [43] (v2.1.13) (Supplementary Figure S1 and the Zenodo repository).

We also clustered these predicted CDS with MMseqs2 linclust [44] (v18.8cc5c) across identity thresholds of 90%, 80%, 70%, 60%, and 50%. Clustering used an 80% bidirectional coverage requirement (`cov-mode 1, -c 0.8`), low-complexity masking, and the fast greedy set-cover algorithm. For each threshold, MMseqs2 produced representative-member assignments; we then performed bootstrap rarefaction on the member-representative mapping by repeatedly subsampling CDSs to increasing depths and counting the number of unique clusters observed (100 steps, and 250 bootstraps). Replicates were aggregated as mean ± confidence intervals to generate rarefaction curves of unique protein clusters against the number of sampled CDSs.

Biosynthetic gene clusters (BGCs) were identified using antiSMASH [45] (v7) on the entire hybrid assembly, with the options `--fullhmmer --clusterhmmer --tigrfam --asf --cc-mibig --cb-general --cb-subclusters --cb-knownclusters --pfam2go --rre --smcog-trees --tfb`. Identified BGCs from the hybrid assembly were subsequently clustered into gene cluster families (GCFs) using BiG-SCAPE [46] (v2 beta5), based on domain architecture and sequence similarity, at a category-level distance threshold of 0.2. The novelty of BGCs was assessed by comparisons against the MIBiG 4.0 [47] database by the BiG-SCAPE search; and independently against the BGC Atlas [48] database by using its built-in BiG-SLiCE [49] search functionality at the pre-defined distance threshold of 0.4. The BGCs were classified as "novel" when they did not cluster with any MIBiG reference BGC or BGC Atlas reference GCF.

Reconstruction of metagenome-assembled genomes

Metagenome-assembled genomes (MAGs) were recovered using a combination of complementary binning strategies to maximise genome quality. Binning was first performed independently on the final NextPolish-corrected assembly using COMEBin [50], MetaDecoder [51], SemiBin2 [52], and VAMB [53], executed in a CUDA-enabled environment when possible.

COMEBin (v 1.0.4), together with CheckM (v 1.1.3) [54], was run on alignments generated by minimap2 [26] (v2.28-r1209), with the following parameters: `NUM_VIEWS=6`, `TEMPERATURE=0.07`, `EMBEDDING_SIZE=2048`, `COVERAGE_EMBEDDING_SIZE=2048`, and `BATCH_SIZE=1024`. MetaDecoder (v 1.1.0) was executed with default parameters according to the authors' recommendations. SemiBin2 (v 2.1.0) was run in both `--self-supervised` and `--semi-supervised` modes, specifying soil as the environment type and `long_reads` as the sequencing type. Gene prediction was performed using Prodigal (v 2.6.3) [55], and the GTDB v95 database [29] was used alongside the previously described minimap2 mappings of the long reads.

For VAMB (v 4.1.4) [53], six binning results were generated by combining default and taxonomy-aware binning modes with three input configurations: long reads, short reads, and both. Abundance profiles were computed using Strobealign [56] (v 0.15.0). For TaxVAMB [57], contig-level taxonomic annotations were derived using Metabuli [28] against the GTDB reference database [29].

In order to produce a final, unified metagenome-assembled-genome binning, the binning results from the above-mentioned ten runs were collected, and refined using MAGScoT [58] (v 1.1). MAGScot was run on gene-calling results from prodigal [55] (v

2.6.3), and hmmsearch results from the HMMER package [59] (v 3.4), using Pfam [42] and TIGRFAMs [60] HMM profile databases.

The resulting MAGs were evaluated for their completeness and contamination with CheckM2 [61] (v 1.0.2). Pairwise genomic distances were computed using Mash [62] (v 2.3) with default parameters. Taxonomic assignments were performed with GTDB-Tk [63] (v 2.4.0) using the GTDB r220 release [29] in classify mode. A phylogenetic tree of high- and medium-quality MAGs was constructed with GTDB-Tk in *de novo* mode and visualised with iTOL [64].

These MAGs were also annotated using Bakta [36] and antiSMASH [45] as described above, both to establish links between BGCs and MAGs and to provide a reference annotation set for future studies (available in the Zenodo repository). Our hybrid (ONT+Illumina) analysis was executed via a Nextflow DSL2 pipeline released on WorkflowHub [65].

Results

Ultra-deep hybrid sequencing and assembly of a temperate forest sample

To evaluate the limits of current sequencing technologies in capturing soil microbial diversity, we generated an ultra-deep metagenomic dataset from a single sample collected in the Schönbuch forest, a temperate mixed forest in southwestern Germany. The sample was taken from the A horizon of a Cambisol soil, a type of soil known for its rich microbial diversity and favourable characteristics for DNA extraction. This particular site and soil type were previously shown to harbour high biosynthetic potential and taxonomic richness [19], and were therefore selected as a representative model system for deep metagenomic exploration.

We extracted total environmental DNA from this soil and sequenced it using a hybrid strategy that combined 148 Gbp of ONT long-read and 122 Gbp of Illumina short-read sequencing. Nanopore reads had a read length N50 of 12.2 kbp, while Illumina reads achieved a mean Q-score of 35. Together, this 270 Gbp dataset represents one of the deepest single-sample soil metagenomes reported to date, and provides a unique opportunity to empirically assess taxonomic and functional complexity.

We employed a hybrid assembly strategy to combine the complementary strengths of long and short reads. Long-read Nanopore data were assembled and subsequently polished using Illumina data, resulting in 10.5 Gbp of assembled sequence with a contig N50 of 77.8 kbp. For comparison, an Illumina-only assembly produced a more fragmented assembly with an N50 of only 865 bp and a total length of 9.2 Gbp, confirming the advantages of long-read inclusion.

Taxonomic analysis reveals unprecedented levels of novelty

Taxonomic classification of both the raw reads and the assembled contigs revealed a highly diverse and complex microbial community, comprising members from 69 different phyla. As expected for soil environments, the community was dominated by taxa such as Pseudomonadota and Acidobacteriota (Figure 1A), along with representatives from many other phyla. A small fraction (approximately 1%) of the reads originated from eukaryotes, while 8% remained unclassified, indicating the presence of deeply novel sequences absent from current databases.

Taxonomic analysis of the assembled contigs (Figure 1B) further demonstrated that a substantial portion of the dataset originates from uncultivated organisms across all taxonomic ranks. While soil has long been recognised as a reservoir of uncultivated diversity, with historical estimates suggesting 90–99% of taxa remain uncultured, our data now provide a quantitative confirmation of this. At the species level, more than 99% of the assembled contigs could not be assigned to any taxon with a cultured representative. Only 0.7% of contigs could be linked to species with at least one cultured strain, and 16.7% to a genus with cultured members. At the species level 4.5%, and at the genus level 21.6% of the contigs were linked to a previously sequenced MAG/SAG; however, they did not have a cultured representative. 94.4% and 61.8% of the assembled data could not be assigned to any genome present in the databases at species and genus levels, respectively.

Even at broader taxonomic levels, substantial novelty is evident: 9% of contigs could not be assigned to any known class (cultured or uncultured), and 6.2% could not be linked to any known phylum (Figure 1B). Among the contigs that were classified, a substantial fraction was affiliated with taxa represented exclusively by uncultivated lineages, known only from metagenomic or single-cell sequencing studies. These patterns are in contrast to metagenomic datasets from more tractable environments, such as the human gut or ocean microbiomes, where a larger fraction of sequenced data can be linked to cultivated taxa [66, 67].

Soil diversity remains undersampled

To evaluate whether ultra-deep sequencing could fully capture the microbial diversity present in the sample, we applied a combination of k-mer analysis, rarefaction based on marker genes, full-length 16S rRNA genes, and predicted CDSs. All approaches consistently indicated that, despite the unprecedented depth of sequencing, the dataset remains far from saturating the underlying biological diversity.

K-mer analysis, which examines the frequency distribution of short subsequences within the sequencing reads, showed a distribution heavily weighted toward low-frequency k-mers (Figure 2A). Most k-mers occurred only once or a few times, reflecting the high prevalence of unique or rare sequences. This pattern is characteristic of highly diverse communities and indicates the presence of many low-abundance taxa whose genomes were either incompletely covered or missed entirely in assembly. A long right-hand tail, corresponding to high-frequency k-mers, likely originated from abundant taxa or repetitive genomic elements (such as rRNA genes). These patterns were consistent across Nanopore and Illumina datasets and highlight the extreme sequence heterogeneity of the sample (Figure 2A, and Supplementary Figures S2 and S3).

Rarefaction analysis with Nonpareil further supported this conclusion. Based on read redundancy, the model projected that approximately 10 terabases of sequencing data would be required to achieve near-complete coverage of the microbial diversity in this single soil sample; almost 50 times more than our current sequencing effort (Figure 2B). This analysis confirms that even the most comprehensive sequencing efforts to date fall short of fully resolving the taxonomic complexity of soil microbiomes.

In parallel, we extracted a total of 19 738 full-length 16S rRNA gene sequences from Nanopore reads (Supplementary Figure S4) and clustered them at various sequence identity thresholds. At 100% and 97% identity, the number of observed 16S rRNA gene clusters continued to increase linearly with sequencing depth, showing no indication of saturation (Figure 3A). A complementary analysis based on 120 conserved single-copy bacterial marker genes showed similar trends, with between 9000 and 59 000 unique copies detected per gene across the dataset (Supplementary Figure S5). While at lower clustering thresholds, the number of representative 16S rRNA gene sequences began to plateau; at higher identity thresholds, the curves remained unsaturated. Similarly, the rarefaction of clustered CDSs showed that observed richness continued to increase even after sampling 10 million CDSs, irrespective of the clustering threshold applied. These steep, non-saturating curves indicate not only unresolved taxonomic diversity but also a remarkably deep functional repertoire present in the soil community.

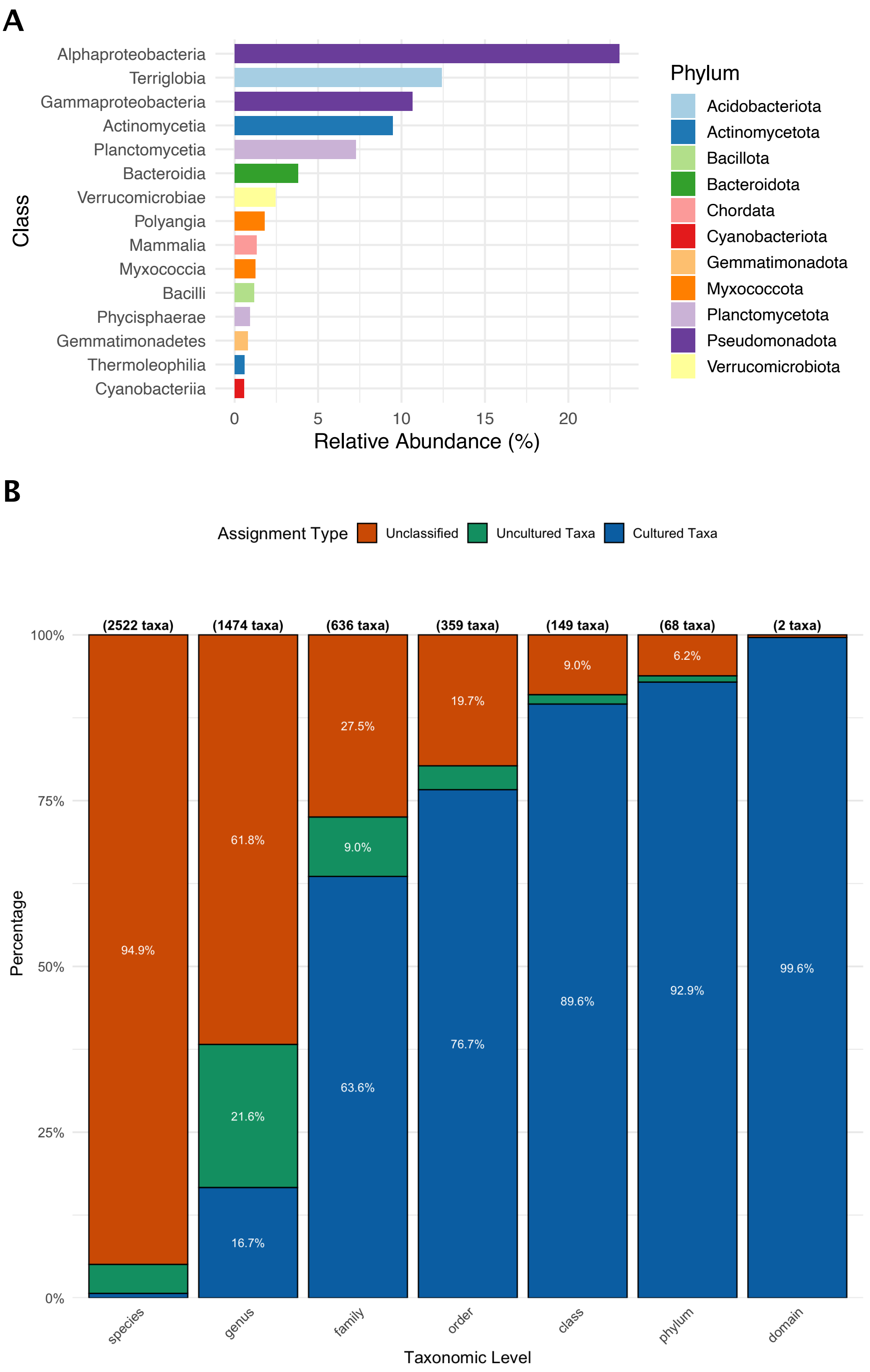

Figure 1. Taxonomic summary of the assembled contigs. **(A)** Class-level relative abundances of the assembled contigs, coloured by the phyla they belong to. **(B)** Taxonomic novelty levels of the assembled contigs across different ranks. Bars show the proportion of total assembled bases assigned to: cultured taxa (blue), where at least one GTDB representative is an isolate genome; uncultured taxa (green), where all GTDB representatives are MAGs/SAGs only; or unclassified (orange), where no assignment was returned at rank *R* (e.g. insufficient similarity or conflicting hits). The number of taxa identified at each level is shown above each bar. Most sequences could only be classified at higher taxonomic ranks, while species- and genus-level assignments were predominantly unclassified.

Taken together, these results provide robust and multi-dimensional evidence that the soil microbial community remains vastly undersampled, even with sequencing efforts far beyond typical practice. The failure to reach saturation was especially apparent

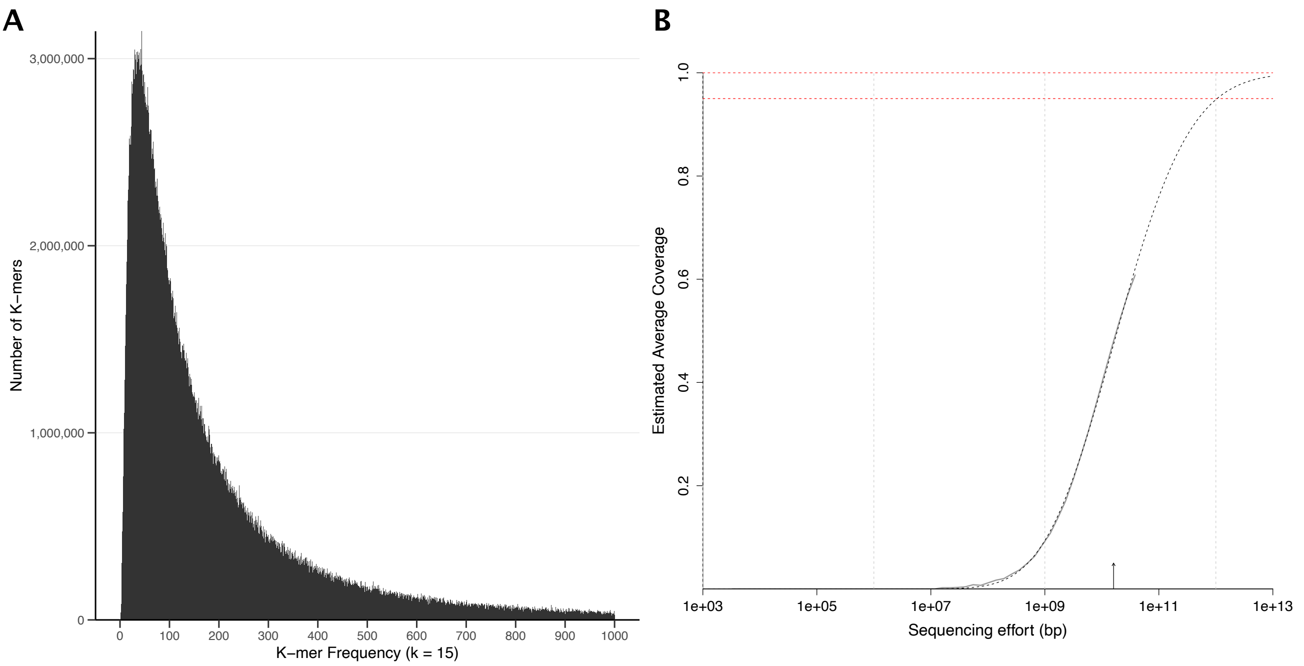

Figure 2. Sequencing depth diagnostics show that ultra-deep sequencing still undersamples the total soil diversity. **(A)** 15-mer spectrum of Nanopore reads from the soil metagenome. Bars show the number of distinct 15-mers observed at each copy number. The pronounced peak at low frequencies reflects a very large pool of unique or low-coverage k-mers, indicating extreme sequence diversity. The long right-hand tail originates from higher coverage k-mers, likely derived from abundant taxa and repetitive genomic regions. **(B)** Nonpareil analysis estimating the sequencing coverage of the pooled Illumina dataset. The curve shows the relationship between sequencing effort and estimated coverage, with the solid line representing the observed dataset and the dashed extrapolation indicating expected gains with additional sequencing. The analysis suggests that substantially more sequencing effort would be required to achieve near-complete coverage of the community.

at the species level, where the rarefaction curves continued to rise linearly across all subsampling depths. This indicates an exceptional degree of fine-scale taxonomic resolution, with many closely related but genomically distinct lineages present at low abundance. Similarly, functional clustering of coding sequences revealed a parallel pattern: at high identity thresholds, the number of distinct CDS clusters continued to grow steeply with sequencing depth, while at lower thresholds, sequences clustered more readily, reflecting both the breadth of novel gene diversity and underlying functional redundancy across lineages.

Such strain- and species-level diversity is particularly important given that many ecologically and functionally relevant traits, such as niche specialisation, symbiosis, and secondary metabolite biosynthesis, can differ even between closely related strains. In more constrained environments, such as the human gut, species-level diversity tends to plateau after a few hundred genomes [68], and marine planktonic communities exhibit relatively stable core microbiomes [69]. By contrast, the soil microbiome appears to exhibit near-limitless diversity at the genomic level.

These findings not only demonstrate the limitations of current sequencing depths but also call into question the accuracy of historical species richness estimates derived from much shallower data. They suggest that achieving anything approaching species-level saturation in soil will likely require terabase- to petabase-scale sequencing efforts, combined with refined computational methods to resolve low-abundance and highly diverse taxa. They demonstrate that many of the diversity estimates historically used in soil microbiome research, often based on much shallower sequencing, likely underestimate the true scale of taxonomic complexity.

Long-read metagenomics recovers thousands of novel and genome-linked BGCs

From the hybrid metagenomic assembly, we identified a total of 11 381 BGCs, including 5652 classified as complete, reflecting the improved contiguity made possible through long-read sequencing. This proportion of complete clusters (~50%) represents a substantial improvement over previous metagenomic surveys, where fewer than 10% of BGCs are typically annotated as complete [48].

Clustering the identified BGCs with BiG-SCAPE resulted in 10 215 gene cluster families, indicating that most BGCs in our dataset are non-redundant and functionally distinct. The size distribution of GCFs (Figure 4A) was highly skewed: most families consisted of singletons or doubletons, with only a handful of larger families, further illustrating the functional uniqueness and fine-grained metabolic specialisation within the community. The largest GCF contains 115 ribosomally synthesised and post-transcriptionally modified peptide (RiPP) BGCs. The cumulative ordering of the GCFs by size shows that the largest eight families account for 3.1% of all BGCs, whereas each of the remaining over ten thousand families contributes only very little to the total number (Figure 4B).

Taxonomic classification of the BGC-containing contigs revealed that biosynthetic potential is broadly distributed across the phylogenetic spectrum. While well-known producers such as Actinomycetota and Pseudomonadota were represented, we also detected numerous BGCs in less-studied phyla such as Verrucomicrobiota and Acidobacteriota (Figure 4C). These groups have historically been overlooked due to cultivation barriers but appear to harbour substantial secondary metabolic capacity.

Only 108 BGCs (~ 1%) had significant matches to known GCFs in the BGC Atlas database, which compiles nearly two million BGCs from metagenomic sources, and none clustered together with reference BGCs from MIBiG. This confirms earlier observations that soil metagenomes are rich in biosynthetic novelty [15, 70], but also demonstrates that even with massive reference expansions, a typical soil still contains an overwhelming majority of uncharacterised BGCs. Notably, our dataset contains more novel BGCs than reported in most large-scale studies of marine or human microbiomes, where the rate of novel BGC discovery is often constrained by lower microbial diversity and shorter contigs [70, 48].

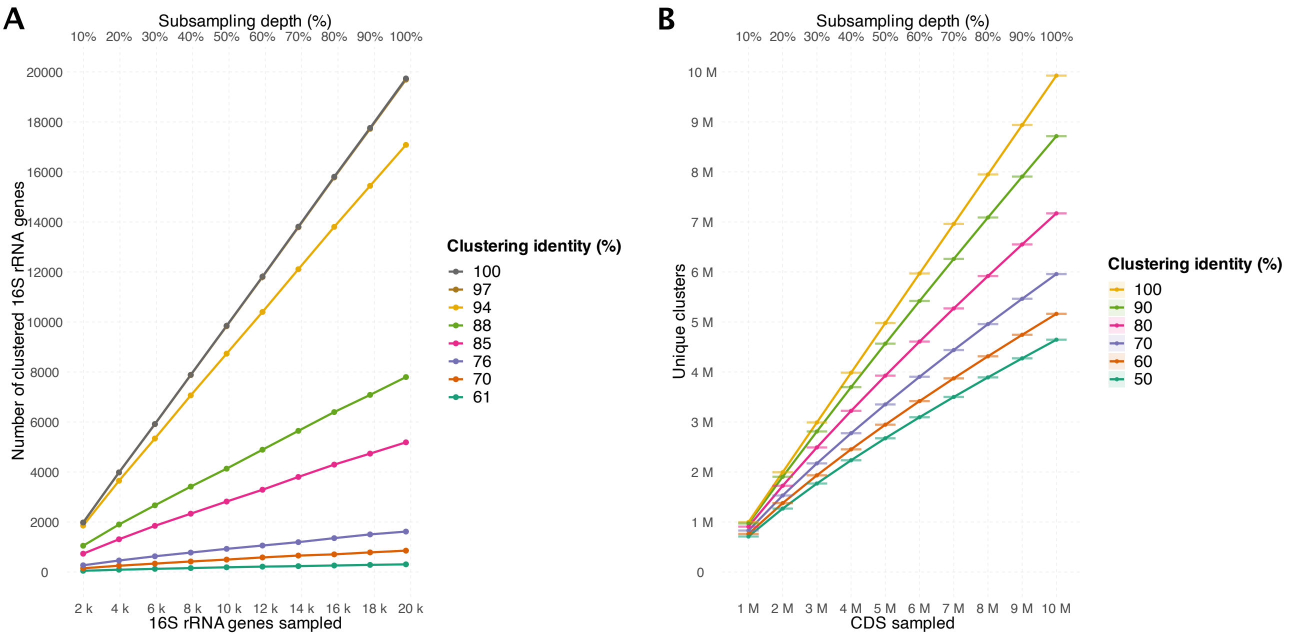

Figure 3. Rarefaction curves of full-length 16S rRNA gene sequences extracted from Nanopore reads (A) and CDSs (B) at different clustering thresholds. **(A)** The number of observed full-length 16S rRNA gene sequences is plotted against the number of sampled genes at clustering thresholds ranging from 61% to 100% sequence identity. The curves at high-identity thresholds, such as 97% and 100%, increase in a near-linear fashion, indicating that the vast majority of taxonomic diversity remains undiscovered, while the increase is less steep at lower clustering thresholds. **(B)** Rarefaction curves of predicted coding sequences (CDSs) from the final assembly, clustered at identity thresholds from 50% to 100%, with a bi-directional coverage requirement of 80%. The number of unique protein clusters continues to rise steeply even after sampling 10 million CDSs, demonstrating that the functional gene repertoire of the soil community is also far from saturated.

Hybrid assembly recovers hundreds of genomes with high taxonomic and biosynthetic novelty

From the hybrid assembly, we recovered 837 metagenome-assembled genomes (MAGs), of which 466 met the MIMAG standards for high- or medium-quality [71]. High- and medium-quality MAGs ranged in size from 1 Mbp (Patescibacteria) to 15 Mbp (Planctomycetota) (Figure 5 and Supplementary Table S1). All MAGs recovered represented unique species-level units, defined by > 0.05 pairwise Mash distance ($\geq$ 95% ANI). These genomes spanned a wide range of bacterial phyla typical of soils, including Acidobacteriota, Pseudomonadota, Verrucomicrobiota, Actinomycetota, and Myxococcota. Remarkably, only four of the 837 MAGs had species-level matches ($\geq$95% ANI) in the GTDB database, and none had cultured representatives.

Thirteen MAGs were assigned to phyla with no cultured members, such as Desulfobacterota, and candidate phyla radiation (CPR) groups FCPU426 and JAJYCY01, harbouring between 2 and 19 BGCs (Figure 5). At other taxonomic levels, 9, 45, 155, and 611 MAGs belong to novel lineages that correspond to class, order, family, and genus levels, respectively.

Many of the recovered MAGs are assigned to taxonomic groups that do not correspond to any known families or even classes in current reference databases (Figure 5). This points to a substantial fraction of microbial diversity in soil that remains undescribed, even at higher taxonomic ranks. In addition to their taxonomic novelty, many of these genomes contain a large number of BGCs, in some cases reaching 30 distinct clusters within a single genome. This suggests that the potential for secondary metabolite production is not restricted to well-characterised soil taxa (such as Actinomycetota), but is also widespread among poorly characterised lineages.

Of all BGCs identified, 6279 could be linked to metagenome-assembled genomes, enabling genome-resolved exploration of natural product potential. This represents one of the largest genome-resolved inventories of BGCs from a single environmental sample to date.

The ability to recover thousands of complete and genome-linked BGCs from a single soil metagenome demonstrates the power of ultra-deep long-read sequencing in capturing microbial metabolic potential. In addition, the high novelty rate and wide taxonomic distribution of these clusters emphasise a vast and largely untapped chemical repertoire unlikely to be captured through shallow sequencing or cultivation-based surveys alone.

Discussion

Soil diversity still outruns ultra-deep sequencing

Our study presents one of the most comprehensive metagenomic investigations of a single soil microbiome to date, combining ultra-deep Oxford Nanopore and Illumina sequencing to yield nearly 300 Gbp of data. This effort enabled high-quality hybrid assemblies and genome-resolved analysis of taxonomic and functional potential in a temperate forest soil sample, providing an empirical benchmark for the capabilities and current limitations of metagenomic sequencing in one of the most complex microbial habitats on Earth.

Despite the ultra-deep sequencing depth, our analyses demonstrate that the microbial diversity of the sample remains far from saturated. Rarefaction curves, k-mer profiles, 16S rRNA gene and marker gene analyses, and functional clustering of CDSs consistently indicate that substantially more sequencing would be required to approach near-complete coverage of the community diversity, with a non-parametric model estimate on the order of $\sim$ 10 Tbp of raw sequencing data to reach high-coverage targets (Figure 2). By comparison, MAG recovery in human-gut communities reaches saturation with $\sim$ 5–10 Gbp per sample, and 25 Gbp is estimated to be enough even to classify taxa of very low abundances (< 1e-06) [72]. Similarly, Ni et al. [73] estimate this number to be around 7

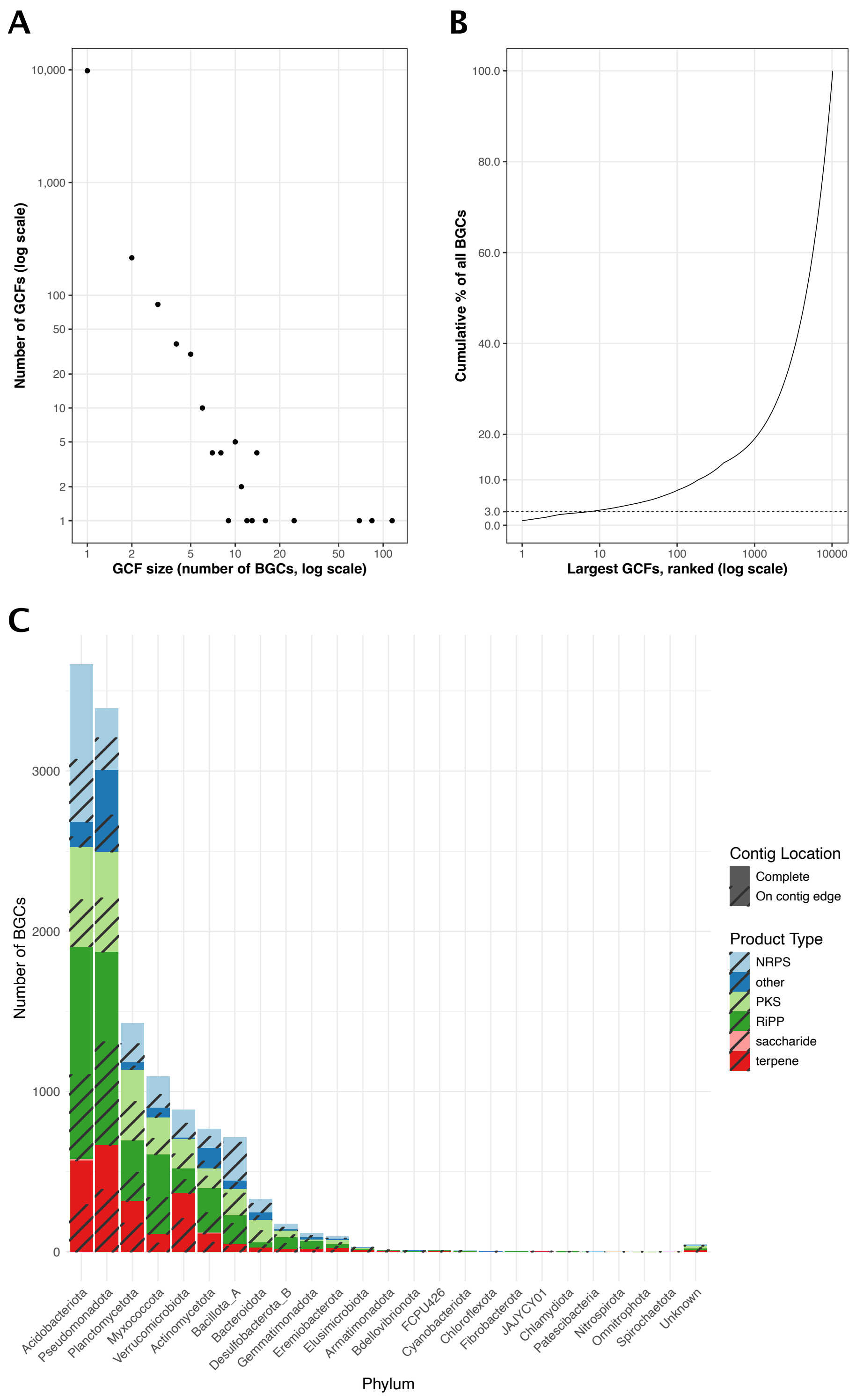

Figure 4. The diversity and taxonomic composition of the biosynthetic gene clusters. **(A)** The size distribution of gene cluster families (GCFs). Each dot represents one distinct GCF size in the dataset (11 381 biosynthetic gene clusters grouped into 10 215 GCFs). The x-axis shows the family size (number of BGCs), the y-axis shows the number of families of that size; both axes are $\log_{10}$-scaled. The plot reveals a heavy tail: 9813 families (96.1 %) are singletons, only 20 families contain $\geq$10 BGCs, and the largest family comprises 115 BGCs. **(B)** Cumulative contribution of the largest GCFs to the total BGC dataset. Families are ranked from largest to smallest along the $\log_{10}$-scaled x-axis; the y-axis tracks the running percentage of all 11 381 BGCs accounted for as each additional family is added. The eight largest GCFs explain 3.1% of all BGCs, whereas 7939 families are required to reach 80%. The rapid rise followed by a very shallow tail shows that a small handful of families contribute to about 3% of BGCs in the dataset, while thousands of minor families are unique. **(C)** Distribution of biosynthetic gene cluster (BGC) product types across bacterial phyla. Each bar represents the total number of BGCs assigned to a given phylum, coloured by predicted product type. Striped regions indicate the proportion of BGCs classified as "on-contig-edge" by antiSMASH. Phyla are ordered by total BGC count, with "Unknown" denoting contigs for which no confident phylum-level classification could be assigned.

Gbp to detect gene content of organisms with relative abundances over 1% in human faecal samples.

Ultra-deep campaigns such as Tara Oceans have shown that planktonic marine communities also benefit from multi-terabase sequencing, yielding thousands of novel genes and genomes and greatly refining oceanic reference catalogues. Yet, the incremental discovery of truly new sequence space in the ocean has begun to flatten at these depths [69, 74, 75]. In soils, by contrast, every addi-

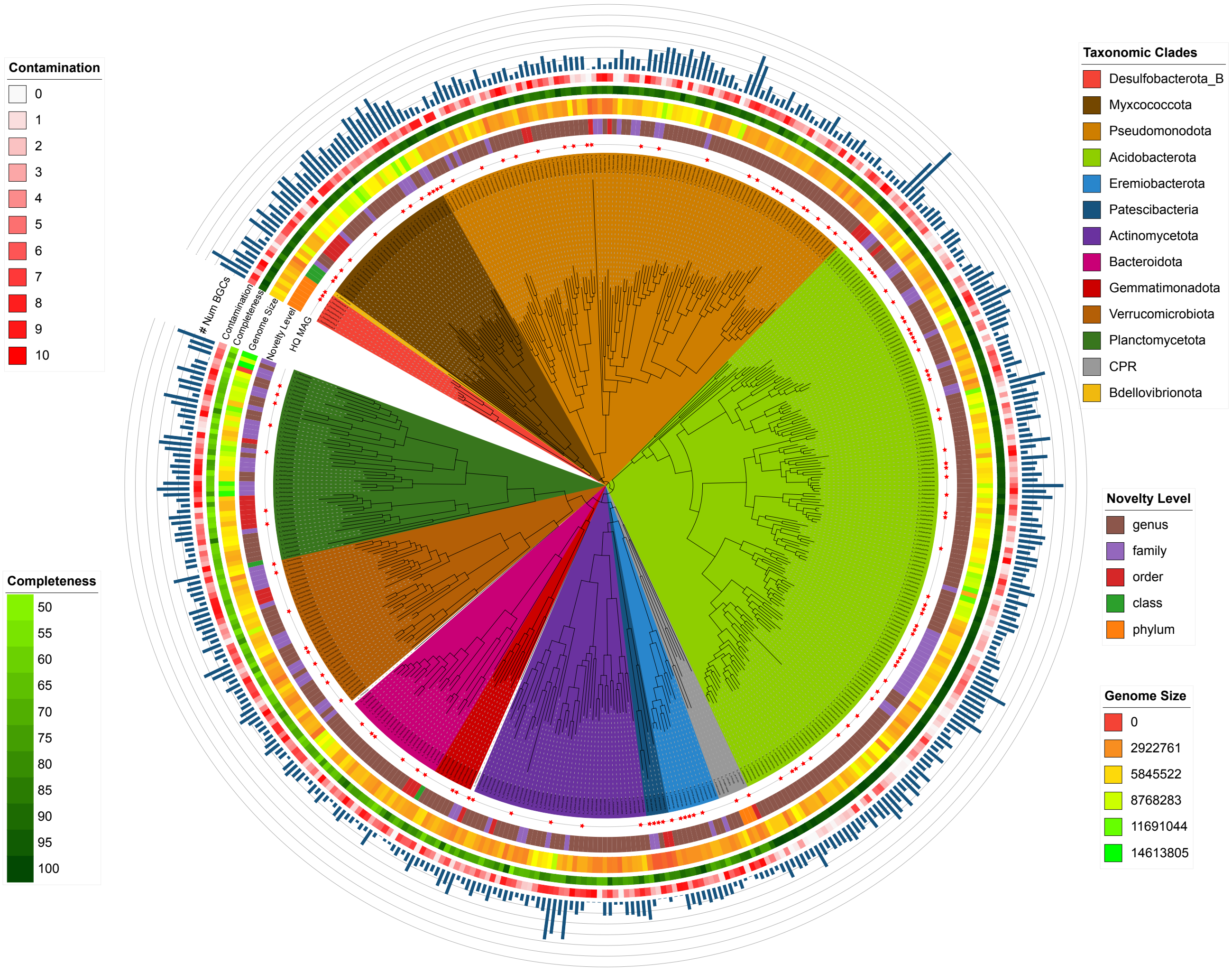

Figure 5. Genome quality statistics and taxonomic classification of newly reconstructed high-quality and medium-quality metagenome-assembled genomes (MAGs). Each leaf represents a MAG, coloured by its assigned phylum. Concentric rings around the leaves indicate, from innermost to outermost: (i) HQ MAGs, (ii) novelty level (genus to phylum), (iii) genome size (bp), (iv) completeness (%), (v) contamination (%), and (vi) the number of BGCs encoded by the MAG (range: 0-30).

tional sequencing effort still brings a steeper growth of previously unseen sequence diversity, and MAGs, with no hint of saturation.

Because our analysis derives from a single temperate Cambisol soil sample, these results should be read as a depth benchmark rather than a census of soils at a global scale. We expect the heavy-tailed diversity and lack of saturation to generalise to other complex soils; however, the taxonomic composition and biosynthetic potential may vary with soil type, season, agricultural use, and other factors.

During the revision of this manuscript, three independent studies using broader sampling and alternative designs reached similar conclusions, placing our single-site depth benchmark in a broader context. A multi-habitat long-read resource sequenced 154 Danish soils with an emphasis on expanding reference space across habitats, and recovered more than 15 000 previously undescribed species and thousands of complete BGCs, demonstrating how long reads broadly improve genome recovery and downstream annotation across terrestrial microbiomes [76]. They also demonstrated a near-linear rarefaction relationship between the number of MAGs that are recovered from the soil samples with the number of species they correspond to. Complementarily, another analysis of publicly available short-read sequencing efforts of soil samples estimated that multi-terabase sequencing efforts per sample may be needed to approach near-complete saturation, consistent with our observation that soil diversity remains unsaturated at current depths [77]. They also demonstrate that large co-assembly efforts of short-read soil metagenomics improve the coverage, yet still fall short of capturing it completely. Additionally, Burian et. al. [78] introduced the first terabase-scale long-read sequencing effort of a single soil sample, focusing on the recovery of nonribosomal peptide (NRPS) BGCs. They generated 2.5 Tbp of raw long-read data from one forest soil, where the NRPS discovery scaled linearly up to the 2.5 of the generated data, not capturing the full diversity of NRPS BGCs in the single soil sample.

Uncharted novelty of soil microbes and their functional capabilities

Taxonomic profiling of assembled contigs revealed the presence of a diverse range of phyla in the soil, including the expected dominance of well-known soil phyla, such as Acidobacteriota, Actinomycetota, and Pseudomonadota, alongside under-studied lineages. Yet, over 95% of contigs could not be assigned to a species currently present in databases, and less than 0.7% could be linked to a species with at least one cultured representative. We recovered MAGs that represent candidate novel phyla, highlighting the ability of genome-resolved metagenomics to reveal deeply divergent lineages missed by conventional approaches. Only four MAGs recovered in this study share $\geq$ 95% ANI with genomes in GTDB, and none correspond to cultured isolates, reaffirming how incompletely current databases represent soil ecosystems. These lineages

not only expand the bacterial tree of life but, in many cases, also harbour unique biosynthetic gene clusters, suggesting functional capacities that remain entirely uncharted.

The scale of biosynthetic diversity uncovered is similarly striking. We identified over 11 000 BGCs, more than 5600 of which are complete and many of which could be linked to MAGs. Fewer than 1% of these BGCs matched known families in public databases, suggesting that soils remain a massive and underexplored reservoir for natural product discovery. In other words, the chemistry encoded in a single forest soil sample still sits almost entirely outside the reach of current reference catalogues. The heavy-tailed GCF distribution we observe, where more than 96% of families are singletons, suggests that every additional increment of sequencing continues to reveal functionally distinct BGCs, rather than finding copies of known ones.

These BGCs come from virtually every major bacterial phylum detected in the sample, from well-studied Actinomycetota and Pseudomonadota to under-explored Acidobacteriota, Verrucomicrobiota, and candidate phyla radiation groups. This phylogenetic breadth and the underestimated functional diversity of soil microbes indicate that the soil's metabolic repertoire is not restricted to a few prolific lineages but is dispersed across the microbial tree of life, with implications for microbial interactions, nutrient cycling, defensive traits, and overall ecosystem stability.

Furthermore, our dataset shows that long-read sequencing can address common limitations of metagenomics approaches, including incomplete recovery of BGCs and fragmented assemblies, thereby enabling genome-resolved assessments of secondary metabolism. In contrast to earlier studies depending exclusively on short-read data [48], our approach yields a high proportion of complete BGCs and facilitates the association of biosynthetic capacity with uncultured taxa.

Conclusions

Together, these results redefine the scale of the unknown in soil microbiomes. They demonstrate that even the deepest sequencing efforts to date barely scratch the surface of microbial and metabolic diversity, particularly in complex environments. Moving forward, terabase- to petabase-scale sequencing will likely be necessary to achieve species-level saturation in soil. However, deeper sequencing alone will not be sufficient. Advances in bioinformatics for assembly, binning, and functional annotation, combined with complementary data from metatranscriptomics, metabolomics, and single-cell approaches, will be essential for translating sequence data into ecological and biochemical insight.

In conclusion, this study sets a new reference point for what is currently achievable in soil metagenomics. By combining ultra-deep sequencing with advanced computational methods, we provide an updated, data-driven view of soil microbial and biosynthetic diversity. The dataset offers an empirical baseline for evaluating sequencing depth, genome recovery, and biosynthetic novelty in complex environments. As such, it can serve as a valuable resource for guiding future studies, benchmarking new tools, and refining diversity estimates. Our findings highlight both the power of current technologies and the need for continued investment in sequencing and analysis to uncover the vast, largely uncharacterised microbial life in soil ecosystems.

Potential implications

Beyond its soil-ecological focus, this ultra-deep, genome-resolved dataset can (i) act as a benchmark for soil diversity in future studies. (ii) It supplies hundreds of genomes from previously unseen lineages to extend genome databases and taxonomies such as MGnify [79, 80] and GTDB [29], and refine the bacterial tree of life. (iii) It contains >11 000 largely novel and complete BGCs, many linked to host genomes, which can extend the secondary metabolite databases such as BGC Atlas [48] and the Secondary Metabolism Collaboratory (SMC) [81]; (iv) and this phylogenetically diverse and novel collection of genomes and BGCs can be used as a training set for machine-learning models that predict compound functions [82] and structures [83], and the ecological traits of microbes [84].

Availability of source code and requirements

- Project name: Soil Metagenome Pipeline
- Project home page: https://github.com/ZiemertLab/soil-metagenome-pipeline
- WorkflowHub SEEK ID: https://workflowhub.eu/workflows/1960?version=1
- License: GNU General Public License v3.0

System requirements

- Operating system(s): Linux
- Programming language: Nextflow DSL2, Python, bash
- Other requirements: conda, SLURM
- Hardware requirements: >64 threads, >128GB RAM

Data availability

The raw sequencing data generated during the current study are available in the ENA repository [1]. The identified biosynthetic gene clusters (BGCs), and extracted MAGs are available via Zenodo for unrestricted reuse [2, 3]. The computational analysis pipeline has been made available on GitHub [85] with the GNU General Public License v3.0 and deposited on workflowhub.eu [65] as a NextFlow pipeline.

Additional Files

Supplementary Fig. S1. Distribution of predicted proteins into COG categories.

Supplementary Fig. S2. 17-mer spectrum of Illumina reads.

Supplementary Fig. S3. 17-mer spectrum of Nanopore reads

Supplementary Fig. S4. Length distribution of the 16S rRNA gene sequences extracted from raw Nanopore reads.

Supplementary Fig. S5. The number of occurrences of each marker gene from bac120 set in raw Nanopore reads.

Supplementary Table S1. The details of the extracted MAGs, including their completeness and contamination values, taxonomic classification, assembly statistics, genome annotations statistics, and the number of BGCs identified.

Declarations

List of abbreviations

ANI: average nucleotide identity; BGC: biosynthetic gene cluster; bp: base pairs; CDS: coding sequence; CPR: Candidate phyla radiation; DNA: deoxyribonucleic acid; ENA: European Nucleotide Archive; Gbp: giga (billion) base pairs; GCF: gene cluster family; GTDB: Genome Taxonomy Database; HQ: high quality; MAG: metagenome-assembled genome; MIBiG: Minimum Information about a Biosynthetic Gene Cluster; MIMAG: Minimum Information

about a Metagenome-Assembled Genome; MQ: medium quality; NGS: next-generation sequencing; NRPS: non-ribosomal peptide synthetase; kbp: kilo (thousand) base pairs; Mbp: mega (million) base pairs; ONT: Oxford Nanopore Technologies; PKS: polyketide synthase; Q-score: quality score (Phred); RiPP: ribosomally synthesised and post-transcriptionally modified peptide

Ethical Approval

Not applicable

Consent for publication

Not applicable

Competing Interests

The authors declare that they have no competing interests.

Funding

This work was supported by Bundesministerium für Bildung und Forschung (BMBF), MicroMatrix Project, 161L0284C to N.Z., and Deutsches Zentrum für Infektionsforschung (DZIF), Applied Natural Products for Genome Mining, TTU09.716 to N.Z.

Authors' Contributions

C.B. and N.Z. wrote the main manuscript. C.B. performed the data analysis and prepared the figures. T.N. conducted the soil sampling and DNA isolation. E.A.B. carried out the Nanopore sequencing. C.G. performed basecalling and preliminary quality control of the sequencing data. C.B., S.O., and N.Z. conceptualised the study. All authors reviewed the manuscript.

Acknowledgements

The authors acknowledge the support by the High Performance and Cloud Computing Group at the Zentrum für Datenverarbeitung of the University of Tübingen and the Federal Ministry of Education and Research (BMBF) through grant no. 031 A535A. We thank the Interfaculty Institute for Biomedical Informatics (IBMI) at the University of Tübingen for providing the computational resources essential for this study. NGS sequencing methods were performed with the support of the DFG-funded NGS Competence Center Tübingen (INST 37/1049-1). Additionally, we thank the Deutsche Forschungsgemeinschaft (DFG, German Research Foundation) under Germany's Excellence Strategy—EXC 2124—390838134 for structural support.

References

1. Bağcı C, Negri T, Buena-Atienza E, Gross C, Ossowski S, Ziemert N, BioProject PRJEB89893 [Dataset]. ENA; 2025. https://www.ebi.ac.uk/ena/browser/view/PRJEB89893.
2. Bağcı C, Negri T, Buena-Atienza E, Gross C, Ossowski S, Ziemert N, MAGs and their antiSMASH annotations from an ultra-deep sequencing effort of a forest sample in Schönbuch, Germany [Dataset]. Zenodo; 2025. https://doi.org/10.5281/zenodo.15866786.
3. Bağcı C, Negri T, Buena-Atienza E, Gross C, Ossowski S, Ziemert N, antiSMASH and eggNOG mapper annotations from an ultra-deep sequencing effort of a forest sample in Schönbuch, Germany [Dataset]. Zenodo; 2025. https://doi.org/10.5281/zenodo.17162541.
4. Fierer N. Embracing the unknown: disentangling the complexities of the soil microbiome. Nature Reviews Microbiology 2017;15(10):579–590. https://doi.org/10.1038/nrmicro.2017.87.
5. Roesch LF, Fulthorpe RR, Riva A, Casella G, Hadwin AK, Kent AD, et al. Pyrosequencing enumerates and contrasts soil microbial diversity. The ISME Journal 2007;1(4):283–290. https://doi.org/10.1038/ismej.2007.53.
6. Torsvik V, Øvreås L. Microbial diversity and function in soil: from genes to ecosystems. Current Opinion in Microbiology 2002;5(3):240–245. https://doi.org/10.1016/S1369-5274(02)00324-7.
7. Jousset A, Bienhold C, Chatzinotas A, Gallien L, Gobet A, Kurm V, et al. Where less may be more: how the rare biosphere pulls ecosystems strings. The ISME Journal 2017;11(4):853–862. https://doi.org/10.1038/ismej.2016.174.
8. Lok C. Mining the microbial dark matter. Nature 2015;522(7556):270. https://doi.org/10.1038/522270a.
9. Hug LA, Baker BJ, Anantharaman K, Brown CT, Probst AJ, Castelle CJ, et al. A new view of the tree of life. Nature Microbiology 2016;1(5):1–6. https://doi.org/10.1038/nmicrobiol.2016.48.
10. Lloyd KG, Steen AD, Ladau J, Yin J, Crosby L. Phylogenetically novel uncultured microbial cells dominate earth microbiomes. mSystems 2018;3(5):e00055–18. https://doi.org/10.1128/mSystems.00055-18.
11. Nascimento Lemos L, Manoharan L, William Mendes L, Monteiro Venturini A, Satler Pylro V, Tsai SM. Metagenome assembled-genomes reveal similar functional profiles of CPR/Patescibacteria phyla in soils. Environmental Microbiology Reports 2020;12(6):651–655. https://doi.org/10.1111/1758-2229.12880.
12. Nayfach S, Roux S, Seshadri R, Udwary D, Varghese N, Schulz F, et al. A genomic catalog of Earth's microbiomes. Nature Biotechnology 2021;39(4):499–509. https://doi.org/10.1038/s41587-020-0718-6.
13. Parks DH, Rinke C, Chuvochina M, Chaumeil PA, Woodcroft BJ, Evans PN, et al. Recovery of nearly 8,000 metagenome-assembled genomes substantially expands the tree of life. Nature Microbiology 2017;2(11):1533–1542. https://doi.org/10.1038/s41564-017-0012-7.
14. Bertrand D, Shaw J, Kalathiyappan M, Ng AHQ, Kumar MS, Li C, et al. Hybrid metagenomic assembly enables high-resolution analysis of resistance determinants and mobile elements in human microbiomes. Nature Biotechnology 2019;37(8):937–944. https://doi.org/10.1038/s41587-019-0191-2.
15. Van Goethem MW, Osborn AR, Bowen BP, Andeer PF, Swenson TL, Clum A, et al. Long-read metagenomics of soil communities reveals phylum-specific secondary metabolite dynamics. Communications Biology 2021;4(1):1302. https://doi.org/10.1038/s42003-021-02809-4.
16. Agustinho DP, Fu Y, Menon VK, Metcalf GA, Treangen TJ, Sedlazeck FJ. Unveiling microbial diversity: harnessing long-read sequencing technology. Nature Methods 2024;21(6):954–966. https://doi.org/10.1038/s41592-024-02262-1.
17. Wick RR, Judd LM, Holt KE. Assembling the perfect bacterial genome using Oxford Nanopore and Illumina sequencing. PLOS Computational Biology 2023;19(3):e1010905. https://doi.org/10.1371/journal.pcbi.1010905.
18. Zhang T, Li H, Jiang M, Hou H, Gao Y, Li Y, et al. Nanopore sequencing: flourishing in its teenage years. Journal of Genetics and Genomics 2024;https://doi.org/10.1016/j.jgg.2024.09.007.
19. Mantri SS, Negri T, Sales-Ortells H, Angelov A, Peter S, Neidhardt H, et al. Metagenomic sequencing of multiple soil horizons and sites in close vicinity revealed novel secondary

metabolite diversity. mSystems 2021;6(5):e01018–21. https://doi.org/10.1128/msystems.01018-21.
20. Negri T, Mantri S, Angelov A, Peter S, Muth G, Eustáquio AS, et al. A rapid and efficient strategy to identify and recover biosynthetic gene clusters from soil metagenomes. Applied Microbiology and Biotechnology 2022;106(8):3293–3306. https://doi.org/10.1007/s00253-022-11917-y.
21. Chen S. Ultrafast one-pass FASTQ data preprocessing, quality control, and deduplication using fastp. iMeta 2023;2(2):e107. https://doi.org/10.1002/imt2.107.
22. Kolmogorov M, Bickhart DM, Behsaz B, Gurevich A, Rayko M, Shin SB, et al. metaFlye: scalable long-read metagenome assembly using repeat graphs. Nature Methods 2020;17(11):1103–1110. https://doi.org/10.1038/s41592-020-00971-x.
23. Oxford Nanopore Technologies, GitHub: medaka (Version 2.0.1). GitHub; 2025. https://github.com/nanoporetech/medaka/releases/tag/v2.0.1.
24. Hu J, Fan J, Sun Z, Liu S. NextPolish: a fast and efficient genome polishing tool for long-read assembly. Bioinformatics 2020;36(7):2253–2255. https://doi.org/10.1093/bioinformatics/btz891.
25. Li H, Durbin R. Fast and accurate short read alignment with Burrows–Wheeler transform. Bioinformatics 2009;25(14):1754–1760. https://doi.org/10.1093/bioinformatics/btp324.
26. Li H. Minimap2: pairwise alignment for nucleotide sequences. Bioinformatics 2018;34(18):3094–3100. https://doi.org/10.1093/bioinformatics/bty191.
27. Li D, Liu CM, Luo R, Sadakane K, Lam TW. MEGAHIT: an ultra-fast single-node solution for large and complex metagenomics assembly via succinct de Bruijn graph. Bioinformatics 2015;31(10):1674–1676. https://doi.org/10.1093/bioinformatics/btv033.
28. Kim J, Steinegger M. Metabuli: sensitive and specific metagenomic classification via joint analysis of amino acid and DNA. Nature Methods 2024;21(6):971–973. https://doi.org/10.1038/s41592-024-02273-y.
29. Parks DH, Chuvochina M, Rinke C, Mussig AJ, Chaumeil PA, Hugenholtz P. GTDB: an ongoing census of bacterial and archaeal diversity through a phylogenetically consistent, rank normalized and complete genome-based taxonomy. Nucleic Acids Research 2022;50(D1):D785–D794. https://doi.org/10.1093/nar/gkab776.
30. Rhie A, Nurk S, Cechova M, Hoyt SJ, Taylor DJ, Altemose N, et al. The complete sequence of a human Y chromosome. Nature 2023;621(7978):344–354. https://doi.org/10.1038/s41586-023-06457-y.
31. Steinegger M, Söding J. MMseqs2 enables sensitive protein sequence searching for the analysis of massive data sets. Nature Biotechnology 2017;35(11):1026–1028. https://doi.org/10.1038/nbt.3988.
32. Mohamadi H, Khan H, Birol I. ntCard: a streaming algorithm for cardinality estimation in genomics data. Bioinformatics 2017;33(9):1324–1330. https://doi.org/10.1093/bioinformatics/btw832.
33. Rodriguez-R LM, Gunturu S, Tiedje JM, Cole JR, Konstantinidis KT. Nonpareil 3: fast estimation of metagenomic coverage and sequence diversity. mSystems 2018;3(3):e00039–18. https://doi.org/10.1128/msystems.00039-18.
34. Seemann T, GitHub: Barrnap (Version 0.9). GitHub; 2025. https://github.com/tseemann/barrnap/releases/tag/0.9.
35. Rognes T, Flouri T, Nichols B, Quince C, Mahé F. VSEARCH: a versatile open source tool for metagenomics. PeerJ 2016;4:e2584. https://doi.org/10.7717/peerj.2584.
36. Schwengers O, Jelonek L, Dieckmann MA, Beyvers S, Blom J, Goesmann A. Bakta: rapid and standardized annotation of bacterial genomes via alignment-free sequence identification. Microbial Genomics 2021;7(11):000685. https://doi.org/10.1099/mgen.0.000685.
37. Galperin MY, Vera Alvarez R, Karamycheva S, Makarova KS, Wolf Y, Landsman D, et al. COG database update 2024. Nucleic Acids Research 2024 11;53(D1):D356–D363. https://doi.org/10.1093/nar/gkae983.
38. Hernández-Plaza A, Szklarczyk D, Botas J, Cantalapiedra CP, Giner-Lamia J, Mende DR, et al. eggNOG 6.0: enabling comparative genomics across 12 535 organisms. Nucleic Acids Research 2023;51(D1):D389–D394. https://doi.org/10.1093/nar/gkac1022.
39. Kanehisa M, Furumichi M, Tanabe M, Sato Y, Morishima K. KEGG: new perspectives on genomes, pathways, diseases and drugs. Nucleic Acids Research 2017;45(D1):D353–D361. https://doi.org/10.1093/nar/gkw1092.
40. The Gene Ontology Consortium SA Aleksander, Balhoff J, Carbon S, Cherry JM, Drabkin HJ, Ebert D, et al. The Gene Ontology knowledgebase in 2023. Genetics 2023;224(1):iyad031. https://doi.org/10.1093/genetics/iyad031.
41. Cantarel BL, Coutinho PM, Rancurel C, Bernard T, Lombard V, Henrissat B. The Carbohydrate-Active EnZymes database (CAZy): an expert resource for Glycogenomics. Nucleic Acids Research 2009;37(suppl_1):D233–D238. https://doi.org/10.1093/nar/gkn663.
42. Mistry J, Chuguransky S, Williams L, Qureshi M, Salazar GA, Sonnhammer EL, et al. Pfam: The protein families database in 2021. Nucleic Acids Research 2021;49(D1):D412–D419. https://doi.org/10.1093/nar/gkaa913.
43. Cantalapiedra CP, Hernández-Plaza A, Letunic I, Bork P, Huerta-Cepas J. eggNOG-mapper v2: functional annotation, orthology assignments, and domain prediction at the metagenomic scale. Molecular Biology and Evolution 2021;38(12):5825–5829. https://doi.org/10.1093/molbev/msab293.
44. Steinegger M, Söding J. Clustering huge protein sequence sets in linear time. Nature Communications 2018;9(1):2542. https://doi.org/10.1038/s41467-018-04964-5.
45. Blin K, Shaw S, Augustijn HE, Reitz ZL, Biermann F, Alanjary M, et al. antiSMASH 7.0: new and improved predictions for detection, regulation, chemical structures and visualisation. Nucleic Acids Research 2023;51(W1):W46–W50. https://doi.org/10.1093/nar/gkad344.
46. Draisma A, Loureiro C, Louwen NL, Kautsar SA, Navarro-Muñoz JC, Doering DT, et al. BiG-SCAPE 2.0 and BiG-SLiCE 2.0: scalable, accurate and interactive sequence clustering of metabolic gene clusters. bioRxiv 2025;https://doi.org/10.1101/2025.08.20.671210.
47. Zdouc MM, Blin K, Louwen NL, Navarro J, Loureiro C, Bader CD, et al. MIBiG 4.0: advancing biosynthetic gene cluster curation through global collaboration. Nucleic Acids Research 2025;53(D1):D678–D690. https://doi.org/10.1093/nar/gkae1115.
48. Bağcı C, Nuhamunada M, Goyat H, Ladanyi C, Sehnal L, Blin K, et al. BGC Atlas: a web resource for exploring the global chemical diversity encoded in bacterial genomes. Nucleic Acids Research 2025;53(D1):D618–D624. https://doi.org/10.1093/nar/gkae953.
49. Kautsar SA, van der Hooft JJ, de Ridder D, Medema MH. BiG-SLiCE: A highly scalable tool maps the diversity of 1.2 million biosynthetic gene clusters. GigaScience 2021;10(1):giaa154. https://doi.org/10.1093/gigascience/giaa154.
50. Wang Z, You R, Han H, Liu W, Sun F, Zhu S. Effective binning of metagenomic contigs using contrastive multi-view representation learning. Nature Communications 2024;15(1):585. https://doi.org/10.1038/s41467-023-44290-z.
51. Liu CC, Dong SS, Chen JB, Wang C, Ning P, Guo Y, et al. MetaDecoder: a novel method for clustering metagenomic contigs. Microbiome 2022;10(1):46. https://doi.org/10.1186/s40168-022-01237-8.

52. Pan S, Zhao XM, Coelho LP. SemiBin2: self-supervised contrastive learning leads to better MAGs for short-and long-read sequencing. Bioinformatics 2023;39(Supplement_1):i21–i29. https://doi.org/10.1093/bioinformatics/btad209.
53. Nissen JN, Johansen J, Allesøe RL, Sønderby CK, Armenteros JJA, Grønbech CH, et al. Improved metagenome binning and assembly using deep variational autoencoders. Nature Biotechnology 2021;39(5):555–560. https://doi.org/10.1038/s41587-020-00777-4.
54. Parks DH, Imelfort M, Skennerton CT, Hugenholtz P, Tyson GW. CheckM: assessing the quality of microbial genomes recovered from isolates, single cells, and metagenomes. Genome Research 2015;25(7):1043–1055. https://doi.org/10.1101/gr.186072.114.
55. Hyatt D, Chen GL, LoCascio PF, Land ML, Larimer FW, Hauser LJ. Prodigal: prokaryotic gene recognition and translation initiation site identification. BMC Bioinformatics 2010;11:1–11. https://doi.org/10.1186/1471-2105-11-119.
56. Sahlin K. Strobealign: flexible seed size enables ultra-fast and accurate read alignment. Genome Biology 2022;23(1):260. https://doi.org/10.1186/s13059-022-02831-7.
57. Kutuzova S, Piera P, Nor Nielsen K, Olsen NS, Riber L, Gobbi A, et al. Binning meets taxonomy: TaxVAMB improves metagenome binning using bi-modal variational autoencoder. bioRxiv 2024;https://doi.org/10.1101/2024.10.25.620172.
58. Rühlemann MC, Wacker EM, Ellinghaus D, Franke A. MAGScoT: a fast, lightweight and accurate bin-refinement tool. Bioinformatics 2022;38(24):5430–5433. https://doi.org/10.1093/bioinformatics/btac694.
59. Eddy SR. Accelerated profile HMM searches. PLOS Computational Biology 2011;7(10):e1002195. https://doi.org/10.1371/journal.pcbi.1002195.
60. Haft DH, Selengut JD, Richter RA, Harkins D, Basu MK, Beck E. TIGRFAMs and genome properties in 2013. Nucleic Acids Research 2012;41(D1):D387–D395. https://doi.org/10.1093/nar/gks1234.
61. Chklovski A, Parks DH, Woodcroft BJ, Tyson GW. CheckM2: a rapid, scalable and accurate tool for assessing microbial genome quality using machine learning. Nature Methods 2023;20(8):1203–1212. https://doi.org/10.1038/s41592-023-01940-w.
62. Ondov BD, Treangen TJ, Melsted P, Mallonee AB, Bergman NH, Koren S, et al. Mash: fast genome and metagenome distance estimation using MinHash. Genome Biology 2016;17:1–14. https://doi.org/10.1186/s13059-016-0997-x.
63. Chaumeil PA, Mussig AJ, Hugenholtz P, Parks DH. GTDB-Tk v2: memory friendly classification with the genome taxonomy database. Bioinformatics 2022;38(23):5315–5316. https://doi.org/10.1093/bioinformatics/btac672.
64. Letunic I, Bork P. Interactive Tree of Life (iTOL) v6: recent updates to the phylogenetic tree display and annotation tool. Nucleic Acids Research 2024;52(W1):W78–W82. https://doi.org/10.1093/nar/gkae268.
65. Bağcı C, Soil Metagenome Pipeline. WorkflowHub; 2025. https://workflowhub.eu/workflows/1960. https://doi.org/10.48546/workflowhub.workflow.1960.1.
66. Blanco-Míguez A, Beghini F, Cumbo F, McIver LJ, Thompson KN, Zolfo M, et al. Extending and improving metagenomic taxonomic profiling with uncharacterized species using MetaPhlAn 4. Nature Biotechnology 2023;41(11):1633–1644. https://doi.org/10.1038/s41587-023-01688-w.
67. Chen J, Jia Y, Sun Y, Liu K, Zhou C, Liu C, et al. Global marine microbial diversity and its potential in bioprospecting. Nature 2024;633(8029):371–379. https://doi.org/10.1038/s41586-024-07891-2.
68. Pasolli E, Asnicar F, Manara S, Zolfo M, Karcher N, Armanini F, et al. Extensive unexplored human microbiome diversity revealed by over 150,000 genomes from metagenomes spanning age, geography, and lifestyle. Cell 2019;176(3):649–662. https://doi.org/10.1016/j.cell.2019.01.001.
69. Sunagawa S, Coelho LP, Chaffron S, Kultima JR, Labadie K, Salazar G, et al. Structure and function of the global ocean microbiome. Science 2015;348(6237):1261359. https://doi.org/10.1126/science.1261359.
70. Navarro-Muñoz JC, Selem-Mojica N, Mullowney MW, Kautsar SA, Tryon JH, Parkinson EI, et al. A computational framework to explore large-scale biosynthetic diversity. Nature Chemical Biology 2020;16(1):60–68. https://doi.org/10.1038/s41589-019-0400-9.
71. Bowers RM, Kyrpides NC, Stepanauskas R, Harmon-Smith M, Doud D, Reddy T, et al. Minimum information about a single amplified genome (MISAG) and a metagenome-assembled genome (MIMAG) of bacteria and archaea. Nature Biotechnology 2017;35(8):725–731. https://doi.org/10.1038/nbt.3893.
72. Kim CY, Lee M, Yang S, Kim K, Yong D, Kim HR, et al. Human reference gut microbiome catalog including newly assembled genomes from under-represented Asian metagenomes. Genome Medicine 2021;13:1–20. https://doi.org/10.1186/s13073-021-00950-7.
73. Ni J, Yan Q, Yu Y. How much metagenomic sequencing is enough to achieve a given goal? Scientific Reports 2013;3(1):1968. https://doi.org/10.1038/srep01968.
74. Duarte CM, Ngugi DK, Alam I, Pearman J, Kamau A, Eguiluz VM, et al. Sequencing effort dictates gene discovery in marine microbial metagenomes. Environmental Microbiology 2020;22(11):4589–4603. https://doi.org/10.1111/1462-2920.15182.
75. Nishimura Y, Yoshizawa S. The OceanDNA MAG catalog contains over 50,000 prokaryotic genomes originated from various marine environments. Scientific Data 2022;9(1):305. https://doi.org/10.1038/s41597-022-01392-5.
76. Sereika M, Mussig AJ, Jiang C, Knudsen KS, Jensen TBN, Petriglieri F, et al. Genome-resolved long-read sequencing expands known microbial diversity across terrestrial habitats. Nature Microbiology 2025;p. 1–13. https://doi.org/10.1038/s41564-025-02062-z.
77. Johansen PL, Chatzigiannidou I, Berzina L, Kristiansen K, Brix S. Unveiling soil microbial diversity through ultra-deep short-read metagenomic sequencing and co-assembly. iMeta 2025;p. e70075. https://doi.org/10.1002/imt2.70075.
78. Burian J, Boer RE, Hernandez Y, Morales-Amador A, Jiang L, Bhattacharjee A, et al. Bioactive molecules unearthed by terabase-scale long-read sequencing of a soil metagenome. Nature Biotechnology 2025;p. 1–8. https://doi.org/10.1038/s41587-025-02810-w.
79. Richardson L, Allen B, Baldi G, Beracochea M, Bileschi ML, Burdett T, et al. MGnify: the microbiome sequence data analysis resource in 2023. Nucleic Acids Research 2023;51(D1):D753–D759. https://doi.org/10.1093/nar/gkac1080.
80. Gurbich TA, Almeida A, Beracochea M, Burdett T, Burgin J, Cochrane G, et al. MGnify genomes: a resource for biome-specific microbial genome catalogues. Journal of Molecular Biology 2023;435(14):168016. https://doi.org/10.1016/j.jmb.2023.168016.
81. Udwary DW, Doering DT, Foster B, Smirnova T, Kautsar SA, Mouncey NJ. The secondary metabolism collaboratory: a database and web discussion portal for secondary metabolite biosynthetic gene clusters. Nucleic Acids Research 2025;53(D1):D717–D723. https://doi.org/10.1093/nar/gkae1060.
82. Larralde M, Zeller G. Machine learning inference of natural product chemistry across biosynthetic gene cluster types. bioRxiv 2025;https://doi.org/10.1101/2025.03.13.642868.
83. Skinnider MA, Johnston CW, Gunabalasingam M, Merwin NJ, Kieliszek AM, MacLellan RJ, et al. Comprehensive prediction of secondary metabolite structure and biologi-

cal activity from microbial genome sequences. Nature Communications 2020;11(1):6058. https://doi.org/10.1038/s41467-020-19986-1.

84. Li Z, Riley WJ, Marschmann GL, Karaoz U, Shirley IA, Wu Q, et al. A framework for integrating genomics, microbial traits, and ecosystem biogeochemistry. Nature Communications 2025;16(1):2186. https://doi.org/10.1038/s41467-025-57386-5.

85. Bağcı C, GitHub: Soil Metagenome Pipeline [Source Code]. GitHub; 2025. https://github.com/ZiemertLab/soil-metagenome-pipeline.

Manuscript

Click here to access/download;Manuscript;ultra-deep-soil-revised.pdf

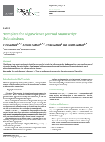

GigaScience, 2025, 1–14

doi: xx.xxxx/xxxx
Manuscript in Preparation
Research

RESEARCH

Ultra-deep long-read metagenomics captures diverse taxonomic and biosynthetic potential of soil microbes

Caner Bağcı [0000-0003-4428-9515][1,2,3], Timo Negri[1,2,3], Elena Buena Atienza [0000-0002-9890-1960][4,5], Caspar Gross [0000-0002-9009-5458][4,5], Stephan Ossowski [0000-0002-7416-9568][4,5] and Nadine Ziemert [0000-0002-7264-1857][1,2,3,*]

[1]Translational Genome Mining for Natural Products, Interfaculty Institute of Microbiology and Infection Medicine Tübingen (IMIT), University of Tübingen, Auf der Morgenstelle 24, 72076, Tübingen, Baden-Württemberg, Germany and [2]Interfaculty Institute for Biomedical Informatics (IBMI), University of Tübingen, Auf der Morgenstelle 24, 72076, Tübingen, Baden-Württemberg, Germany and [3]German Center for Infection Research (DZIF), Partner Site Tübingen, Auf der Morgenstelle 24, 72076, Tübingen, Baden-Württemberg, Germany and [4]Institute of Medical Genetics and Applied Genomics, University of Tübingen, Calwerstrasse 7, 72076, Tübingen, Baden-Württemberg, Germany and [5]NGS Competence Center, University of Tübingen, Calwerstrasse 7, 72076, Tübingen, Baden-Württemberg, Germany

[*]nadine.ziemert@uni-tuebingen.de

Abstract

Background: Soil ecosystems have long been recognised as hotspots of microbial diversity, but most estimates of their microbial and functional complexity remain speculative despite decades of study, in part because conventional sequencing campaigns lack the depth and contiguity required to recover low-abundance and repetitive genomes. Here, we revisit this question using one of the deepest metagenomic sequencing efforts to date, applying 148 billion base pairs of Nanopore long-read and 122 billion base pairs of Illumina short-read data to a single forest soil sample.
Results: Our hybrid assembly reconstructed 837 metagenome-assembled genomes, including 466 that meet high- and medium-quality standards, nearly all lacking close relatives among cultivated taxa. Rarefaction and k-mer analyses reveal that, even at this depth, we capture only a fraction of the extant diversity: non-parametric models project that more than ten trillion base pairs of sequencing data would be required to approach saturation. These findings offer a quantitative, technology-enabled update to long-standing diversity estimates and demonstrate that conventional metagenomic sequencing efforts likely miss the majority of microbial and biosynthetic potential in soil. We further identify more than 11 000 biosynthetic gene clusters, over 99% of which have no match in current databases, underscoring the breadth of unexplored metabolic capacity.
Conclusions: Taken together, our results emphasise both the power and the present limitations of metagenomics in resolving natural microbial complexity, and they provide a new baseline for evaluating future advances in microbial genome recovery, taxonomic classification, and natural product discovery.

Key words: microbiome; soil; metagenomics; nanopore; ultra-deep; long-read sequencing; diversity; natural products; (meta)genome mining

Compiled on: October 22, 2025.
Draft manuscript prepared by the author.

Key Points

- Ultra-deep hybrid sequencing (148 Gbp Nanopore + 122 Gbp Illumina) of a single forest soil sample yielded 837 metagenome-assembled genomes, all lacking cultured counterparts.
- Despite this unprecedented 270 Gbp depth, rarefaction and coverage modelling indicate that more than 10 Tbp of data would still be needed to reach saturation in soil.
- Only 0.7% and 16.7% of all assembled contigs can be assigned to a species and genus, respectively, with at least one cultured representative, highlighting an unprecedented level of novelty in soil.
- The assembly uncovers 11 381 biosynthetic gene clusters forming over 10 000 mostly novel families, spotlighting an immense, untapped reservoir of microbial natural-product potential.

Data Description

In order to quantify how much taxonomic and biosynthetic novelty present-day sequencing can recover from soil, we extracted high-molecular-weight DNA from a single Cambisol forest soil sample (Schönbuch, Germany) and generated an ultra-deep 270 Gbp dataset - 148 Gbp of Oxford Nanopore PromethION long reads (read-length N50 = 12.2 kb) plus 122 Gbp of Illumina NovaSeq reads. The hybrid metagenome assembly we conducted produced a 10.5 Gbp assembly, from which multi-tool binning and subsequent refinement recovered 837 MAGs, and antiSMASH/BiG-SCAPE annotation revealed more than 11 000 largely novel biosynthetic gene clusters, creating a resource for benchmarking assembly or binning pipelines, modelling diversity-coverage relationships and mining natural products; all raw reads are archived under ENA BioProject PRJEB89893 [1], and the polished assembly, MAG set and BGC catalogue are available via Zenodo for unrestricted reuse [2, 3].

Introduction

Soil is one of the most biologically diverse ecosystems on Earth, hosting a massive and largely uncharacterised diversity of microbial life. These microbial communities play critical roles in biogeochemical cycling, soil formation, nutrient turnover, plant health, and climate regulation [4]. Despite their ecological and biotechnological importance, the full extent and functional potential of soil microbiomes remain poorly understood.

A single gram of soil can contain up to 10^9 microbial cells and hundreds of thousands of species, spanning all domains of life [5, 6]. These communities are not only taxonomically complex but also exhibit highly uneven abundance distributions, where rare taxa – collectively termed the "rare biosphere" – may be functionally consequential despite their low abundance [7].

Although the vast diversity of soil microbes has been widely acknowledged for decades, most estimates of their richness are based on indirect approaches, including 16S rRNA gene surveys, limited shotgun datasets, or predictive modelling. As a result, foundational claims about the extent of soil microbial diversity are frequently cited but rarely re-examined with contemporary data. Much of the soil microbiome remains part of the so-called microbial dark matter: lineages with no cultured representatives and no reference genomes [8, 9, 10].

Metagenomics has enabled the cultivation-independent study of microbial communities, allowing for genome-resolved insights into uncultured organisms [11, 12, 13]. However, most metagenomic studies rely on short-read sequencing, which presents challenges in assembling highly complex, strain-rich communities like soil. These limitations hinder the recovery of low-abundance taxa, reduce the completeness of biosynthetic gene clusters, and often lead to fragmented or ambiguous assemblies [12]. Recent advances in long-read sequencing, particularly with Oxford Nanopore Technologies (ONT), have opened new opportunities for soil microbiome research. Long reads can span repeat-rich regions and operons, improving genome recovery and assembly contiguity [14, 15, 16]. Hybrid approaches combining long and short reads can further mitigate the high error rates of long reads while leveraging their structural advantages [17, 18]. Despite these improvements, it remains unclear how far even the most advanced sequencing technologies can go in capturing the full taxonomic and functional diversity of soil.

To establish an empirical reference point for assessing microbial and biosynthetic diversity in soil, we performed one of the deepest metagenomic sequencing efforts to date on a single soil sample, combining 148 billion base pairs (Gbp) of ONT long-read and 122 Gbp of Illumina short-read data. We use this dataset to empirically assess the power and current limitations of metagenomics in resolving complex microbial communities. Specifically, we ask: How much taxonomic and biosynthetic diversity can be recovered from a single soil sample using state-of-the-art sequencing? What proportion of this diversity is represented in existing databases? Can the ultra-deep sequencing approach saturation, or are we still only scratching the surface? By addressing these questions, we aim to provide a data-driven reassessment of microbial diversity in soil and to establish a benchmark for future metagenomic studies of complex ecosystems.

Methods

Sample collection, DNA extraction, and metagenomic sequencing

The A horizon of the soil type Cambisol was sampled from the Schönbuch Forest, near Tübingen, Germany (the same site previously reported by [19]), on 31 May 2022. High molecular weight metagenomic DNA was isolated using a protocol described in detail in our previous studies [19, 20]. Genomic integrity was assessed using pulse-field capillary electrophoresis with the Genomic DNA 165 kb Analysis Kit on a FemtoPulse (Agilent) instrument. Quantitation of DNA was assessed using the dsDNA High Sensitivity Assay on a Qubit 3 fluorometer (Thermo Fisher), and purity was assessed by Nanodrop. A total of 2.4 μg of genomic DNA was used as input for the library preparation with the 1D Ligation Kit SQK-LSK109-XL Sequencing Kit (ONT). Four PromethION R9 flow cells were utilised for long-read Nanopore sequencing, each loaded with 600 ng (50 fmol) of genomic DNA. The raw signal data from the PromethION runs were basecalled with guppy (v 5.0.7) in high-accuracy mode. For complementary short-read sequencing, three NovaSeq 6000 flow cells were loaded with the same isolated DNA; two of them ran with 200 cycles, and one with 300 cycles.

A total of 148 Gbp of Nanopore sequencing data was generated, with a read length N50 of 12.2 kbp. The three Illumina sequencing runs yielded a total of 122 Gbp raw data, with a mean Q-score of 35. The raw Illumina reads were adapter and quality trimmed using fastp [21] (v 0.23.4) with default settings.

Metagenomic assembly

Nanopore reads from all four runs were pooled together and assembled using metaFlye (v2.9.5-b1801) [22] with the `--meta` option optimised for metagenomic data, and the `--nano-raw` option for the error-prone Nanopore reads. The resulting draft assembly was polished with one round of medaka [23] (v 2.0.1) using the `r941_prom_hac_g507` model and the `--bacteria` flag. The medaka polished assembly was further corrected using the trimmed Illumina reads in a single round of NextPolish [24] (v 1.4.1) polishing, with parameters `-max_depth 100` for short-read mapping with bwa [25] (v 0.7.18), and `-min_read_len 1k -max_depth 100 -x map-ont` for long-read mapping with minimap2 [26] (v 2.28-r1209).

In parallel, an Illumina-only assembly was performed using MEGAHIT [27] (v1.2.9) with default metagenomic assembly parameters. All subsequent analyses were based on the hybrid Nanopore-Illumina assembly, which exhibited superior contiguity (contig N50 = 77.8 kbp) and total assembled length (10.5 Gbp).

Taxonomic analysis

Raw reads were taxonomically classified using Metabuli [28] (v 1.0.8), against the precomputed database provided by the authors, which includes GTDB release 214.1 [29] and the human T2T genome [30]. Classification was performed with parameters `--seq-mode 2` for Illumina reads, and `--seq-mode 3` for Nanopore reads. Contigs from the final hybrid assembly were taxonomically classified using MMSeqs2 [31] (v 16.747c6) using the `easy-taxonomy` module, with GTDB release 214.1 [29]. A contig was classified as "cultured" at rank *R*, if its assignment at *R* belongs to a taxon that has at least one genome in GTDB originating from an isolate sequencing; as "uncultured" if an assignment exists at rank *R*, but all representatives of the taxon come from MAG/SAG sequencing efforts; and as "unclassified" if there is no assignment at rank *R* due to insufficient similarity or conflicting similarities. The information on assigned genomes belonging to a cultured or uncultured representative was retrieved from GTDB metadata files.

Rarefaction analysis

Microbial diversity and sequencing coverage were assessed using multiple complementary approaches. K-mer frequency analysis was conducted with ntCard [32] using 15-mers and 17-mers for both Nanopore and Illumina datasets. Nonpareil [33] (v3.3.3) was used to estimate sequence coverage and to project additional sequencing efforts required for complete community recovery using the pooled set of Illumina reads. Full-length 16S rRNA gene sequences were extracted from Nanopore reads using Barrnap [34] (v0.9) (sequences flagged as "partial" were excluded). We performed rarefaction by subsampling them at 10% increments (10-100%), with three independent replicates per depth (distinct random seeds). For each subset, we clustered the subsampled sequences with VSEARCH [35] (v2.15.2), reporting both exact uniques (100% dereplication) and the number of representatives at fixed identity thresholds (e.g. 97%, 94%, 88%, 85%, 70%, 61%). VSEARCH was run with `--strand both --iddef 1 --qmask none` and `--cluster_size` options for maximum sensitivity. In addition, conserved single-copy marker genes from the bac120 set [13] were identified in Nanopore reads using the LAST aligner (v 1615) in frameshift mode (`-F 15`) against the GTDB bac120 reference set, and quantified to independently assess the taxonomic diversity.

Functional annotation, clustering, and biosynthetic gene cluster discovery

The final polished assembly was annotated using Bakta [36] (v 1.11.0, database version 5.1). The identified coding sequences (CDS) were further annotated for their COG categories [37], eggNOG orthologous groups [38], KEGG ontologies, pathways, modules, reactions [39], Gene Ontologies [40], CAZy enzymes [41], and Pfam domains [42], using eggNOG-mapper [43] (v2.1.13) (Supplementary Figure S1 and the Zenodo repository).

We also clustered these predicted CDS with MMseqs2 linclust [44] (v18.8cc5c) across identity thresholds of 90%, 80%, 70%, 60%, and 50%. Clustering used an 80% bidirectional coverage requirement (`cov-mode 1, -c 0.8`), low-complexity masking, and the fast greedy set-cover algorithm. For each threshold, MMseqs2 produced representative-member assignments; we then performed bootstrap rarefaction on the member-representative mapping by repeatedly subsampling CDSs to increasing depths and counting the number of unique clusters observed (100 steps, and 250 bootstraps). Replicates were aggregated as mean $\pm$ confidence intervals to generate rarefaction curves of unique protein clusters against the number of sampled CDSs.

Biosynthetic gene clusters (BGCs) were identified using antiSMASH [45] (v7) on the entire hybrid assembly, with the options `--fullhmmer --clusterhmmer --tigrfam --asf --cc-mibig --cb-general --cb-subclusters --cb-knownclusters --pfam2go --rre --smcog-trees --tfb`. Identified BGCs from the hybrid assembly were subsequently clustered into gene cluster families (GCFs) using BiG-SCAPE [46] (v2 beta5), based on domain architecture and sequence similarity, at a category-level distance threshold of 0.2. The novelty of BGCs was assessed by comparisons against the MIBiG 4.0 [47] database by the BiG-SCAPE search; and independently against the BGC Atlas [48] database by using its built-in BiG-SLiCE [49] search functionality at the pre-defined distance threshold of 0.4. The BGCs were classified as "novel" when they did not cluster with any MIBiG reference BGC or BGC Atlas reference GCF.

Reconstruction of metagenome-assembled genomes

Metagenome-assembled genomes (MAGs) were recovered using a combination of complementary binning strategies to maximise genome quality. Binning was first performed independently on the final NextPolish-corrected assembly using COMEBin [50], MetaDecoder [51], SemiBin2 [52], and VAMB [53], executed in a CUDA-enabled environment when possible.

COMEBin (v 1.0.4), together with CheckM (v 1.1.3) [54], was run on alignments generated by minimap2 [26] (v2.28-r1209), with the following parameters: `NUM_VIEWS=6`, `TEMPERATURE=0.07`, `EMBEDDING_SIZE=2048`, `COVERAGE_EMBEDDING_SIZE=2048`, and `BATCH_SIZE=1024`. MetaDecoder (v 1.1.0) was executed with default parameters according to the authors' recommendations. SemiBin2 (v 2.1.0) was run in both `--self-supervised` and `--semi-supervised` modes, specifying soil as the environment type and `long_reads` as the sequencing type. Gene prediction was performed using Prodigal (v 2.6.3) [55], and the GTDB v95 database [29] was used alongside the previously described minimap2 mappings of the long reads.

For VAMB (v 4.1.4) [53], six binning results were generated by combining default and taxonomy-aware binning modes with three input configurations: long reads, short reads, and both. Abundance profiles were computed using Strobealign [56] (v 0.15.0). For TaxVAMB [57], contig-level taxonomic annotations were derived using Metabuli [28] against the GTDB reference database [29].

In order to produce a final, unified metagenome-assembled-genome binning, the binning results from the above-mentioned ten runs were collected, and refined using MAGScoT [58] (v 1.1). MAGScot was run on gene-calling results from prodigal [55] (v

2.6.3), and hmmsearch results from the HMMER package [59] (v 3.4), using Pfam [42] and TIGRFAMs [60] HMM profile databases.

The resulting MAGs were evaluated for their completeness and contamination with CheckM2 [61] (v 1.0.2). Pairwise genomic distances were computed using Mash [62] (v 2.3) with default parameters. Taxonomic assignments were performed with GTDB-Tk [63] (v 2.4.0) using the GTDB r220 release [29] in classify mode. A phylogenetic tree of high- and medium-quality MAGs was constructed with GTDB-Tk in *de novo* mode and visualised with iTOL [64].

These MAGs were also annotated using Bakta [36] and antiSMASH [45] as described above, both to establish links between BGCs and MAGs and to provide a reference annotation set for future studies (available in the Zenodo repository). Our hybrid (ONT+Illumina) analysis was executed via a Nextflow DSL2 pipeline released on WorkflowHub [65].

Results

Ultra-deep hybrid sequencing and assembly of a temperate forest sample

To evaluate the limits of current sequencing technologies in capturing soil microbial diversity, we generated an ultra-deep metagenomic dataset from a single sample collected in the Schönbuch forest, a temperate mixed forest in southwestern Germany. The sample was taken from the A horizon of a Cambisol soil, a type of soil known for its rich microbial diversity and favourable characteristics for DNA extraction. This particular site and soil type were previously shown to harbour high biosynthetic potential and taxonomic richness [19], and were therefore selected as a representative model system for deep metagenomic exploration.

We extracted total environmental DNA from this soil and sequenced it using a hybrid strategy that combined 148 Gbp of ONT long-read and 122 Gbp of Illumina short-read sequencing. Nanopore reads had a read length N50 of 12.2 kbp, while Illumina reads achieved a mean Q-score of 35. Together, this 270 Gbp dataset represents one of the deepest single-sample soil metagenomes reported to date, and provides a unique opportunity to empirically assess taxonomic and functional complexity.

We employed a hybrid assembly strategy to combine the complementary strengths of long and short reads. Long-read Nanopore data were assembled and subsequently polished using Illumina data, resulting in 10.5 Gbp of assembled sequence with a contig N50 of 77.8 kbp. For comparison, an Illumina-only assembly produced a more fragmented assembly with an N50 of only 865 bp and a total length of 9.2 Gbp, confirming the advantages of long-read inclusion.

Taxonomic analysis reveals unprecedented levels of novelty

Taxonomic classification of both the raw reads and the assembled contigs revealed a highly diverse and complex microbial community, comprising members from 69 different phyla. As expected for soil environments, the community was dominated by taxa such as Pseudomonadota and Acidobacteriota (Figure 1A), along with representatives from many other phyla. A small fraction (approximately 1%) of the reads originated from eukaryotes, while 8% remained unclassified, indicating the presence of deeply novel sequences absent from current databases.

Taxonomic analysis of the assembled contigs (Figure 1B) further demonstrated that a substantial portion of the dataset originates from uncultivated organisms across all taxonomic ranks. While soil has long been recognised as a reservoir of uncultivated diversity, with historical estimates suggesting 90–99% of taxa remain uncultured, our data now provide a quantitative confirmation of this. At the species level, more than 99% of the assembled contigs could not be assigned to any taxon with a cultured representative. Only 0.7% of contigs could be linked to species with at least one cultured strain, and 16.7% to a genus with cultured members. At the species level 4.5%, and at the genus level 21.6% of the contigs were linked to a previously sequenced MAG/SAG; however, they did not have a cultured representative. 94.4% and 61.8% of the assembled data could not be assigned to any genome present in the databases at species and genus levels, respectively.

Even at broader taxonomic levels, substantial novelty is evident: 9% of contigs could not be assigned to any known class (cultured or uncultured), and 6.2% could not be linked to any known phylum (Figure 1B). Among the contigs that were classified, a substantial fraction was affiliated with taxa represented exclusively by uncultivated lineages, known only from metagenomic or single-cell sequencing studies. These patterns are in contrast to metagenomic datasets from more tractable environments, such as the human gut or ocean microbiomes, where a larger fraction of sequenced data can be linked to cultivated taxa [66, 67].

Soil diversity remains undersampled

To evaluate whether ultra-deep sequencing could fully capture the microbial diversity present in the sample, we applied a combination of k-mer analysis, rarefaction based on marker genes, full-length 16S rRNA genes, and predicted CDSs. All approaches consistently indicated that, despite the unprecedented depth of sequencing, the dataset remains far from saturating the underlying biological diversity.

K-mer analysis, which examines the frequency distribution of short subsequences within the sequencing reads, showed a distribution heavily weighted toward low-frequency k-mers (Figure 2A). Most k-mers occurred only once or a few times, reflecting the high prevalence of unique or rare sequences. This pattern is characteristic of highly diverse communities and indicates the presence of many low-abundance taxa whose genomes were either incompletely covered or missed entirely in assembly. A long right-hand tail, corresponding to high-frequency k-mers, likely originated from abundant taxa or repetitive genomic elements (such as rRNA genes). These patterns were consistent across Nanopore and Illumina datasets and highlight the extreme sequence heterogeneity of the sample (Figure 2A, and Supplementary Figures S2 and S3).

Rarefaction analysis with Nonpareil further supported this conclusion. Based on read redundancy, the model projected that approximately 10 terabases of sequencing data would be required to achieve near-complete coverage of the microbial diversity in this single soil sample; almost 50 times more than our current sequencing effort (Figure 2B). This analysis confirms that even the most comprehensive sequencing efforts to date fall short of fully resolving the taxonomic complexity of soil microbiomes.

In parallel, we extracted a total of 19 738 full-length 16S rRNA gene sequences from Nanopore reads (Supplementary Figure S4) and clustered them at various sequence identity thresholds. At 100% and 97% identity, the number of observed 16S rRNA gene clusters continued to increase linearly with sequencing depth, showing no indication of saturation (Figure 3A). A complementary analysis based on 120 conserved single-copy bacterial marker genes showed similar trends, with between 9000 and 59 000 unique copies detected per gene across the dataset (Supplementary Figure S5). While at lower clustering thresholds, the number of representative 16S rRNA gene sequences began to plateau; at higher identity thresholds, the curves remained unsaturated. Similarly, the rarefaction of clustered CDSs showed that observed richness continued to increase even after sampling 10 million CDSs, irrespective of the clustering threshold applied. These steep, non-saturating curves indicate not only unresolved taxonomic diversity but also a remarkably deep functional repertoire present in the soil community.

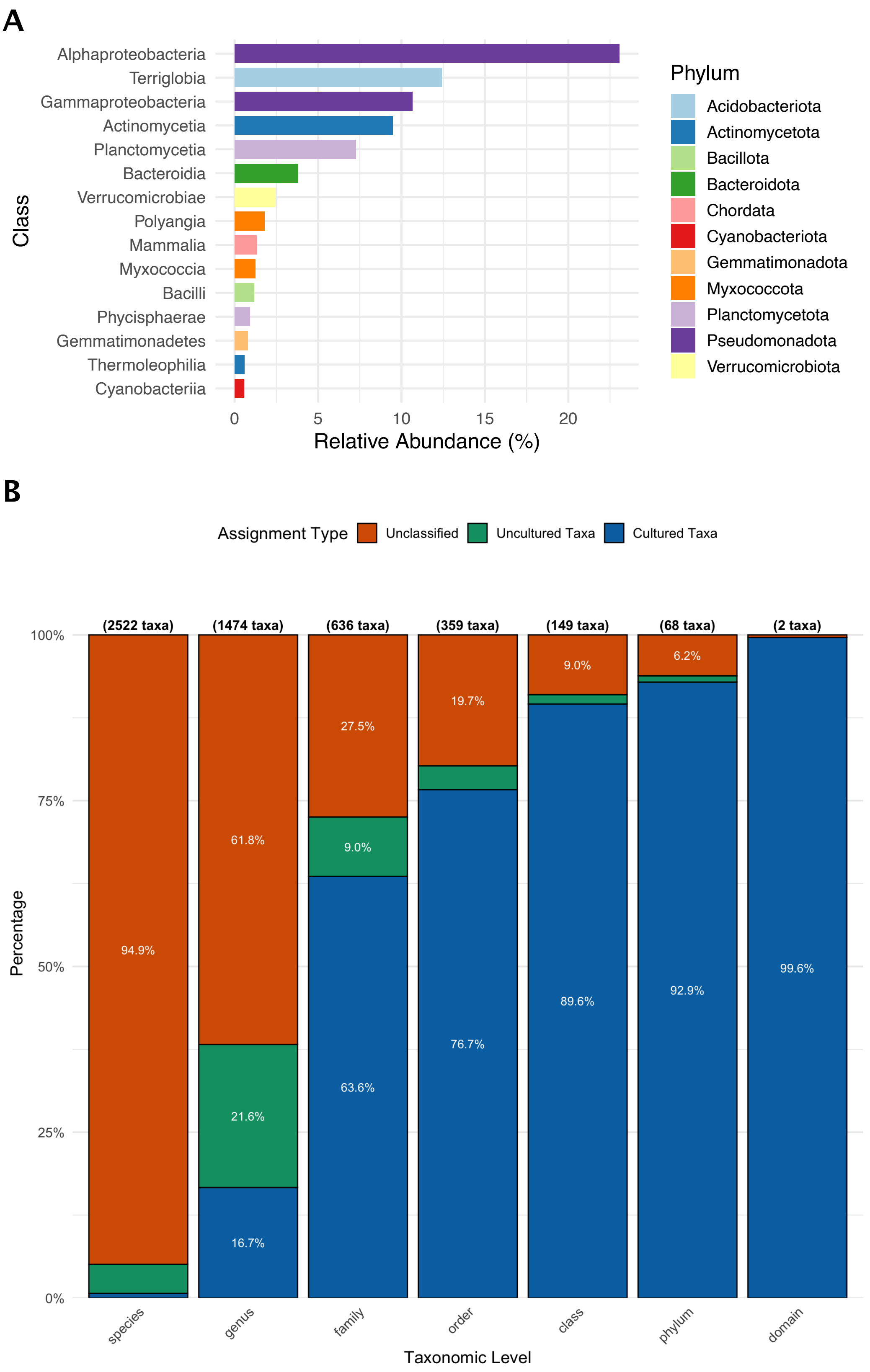

Figure 1. Taxonomic summary of the assembled contigs. **(A)** Class-level relative abundances of the assembled contigs, coloured by the phyla they belong to. **(B)** Taxonomic novelty levels of the assembled contigs across different ranks. Bars show the proportion of total assembled bases assigned to: cultured taxa (blue), where at least one GTDB representative is an isolate genome; uncultured taxa (green), where all GTDB representatives are MAGs/SAGs only; or unclassified (orange), where no assignment was returned at rank *R* (e.g. insufficient similarity or conflicting hits). The number of taxa identified at each level is shown above each bar. Most sequences could only be classified at higher taxonomic ranks, while species- and genus-level assignments were predominantly unclassified.

Taken together, these results provide robust and multi-dimensional evidence that the soil microbial community remains vastly undersampled, even with sequencing efforts far beyond typical practice. The failure to reach saturation was especially apparent

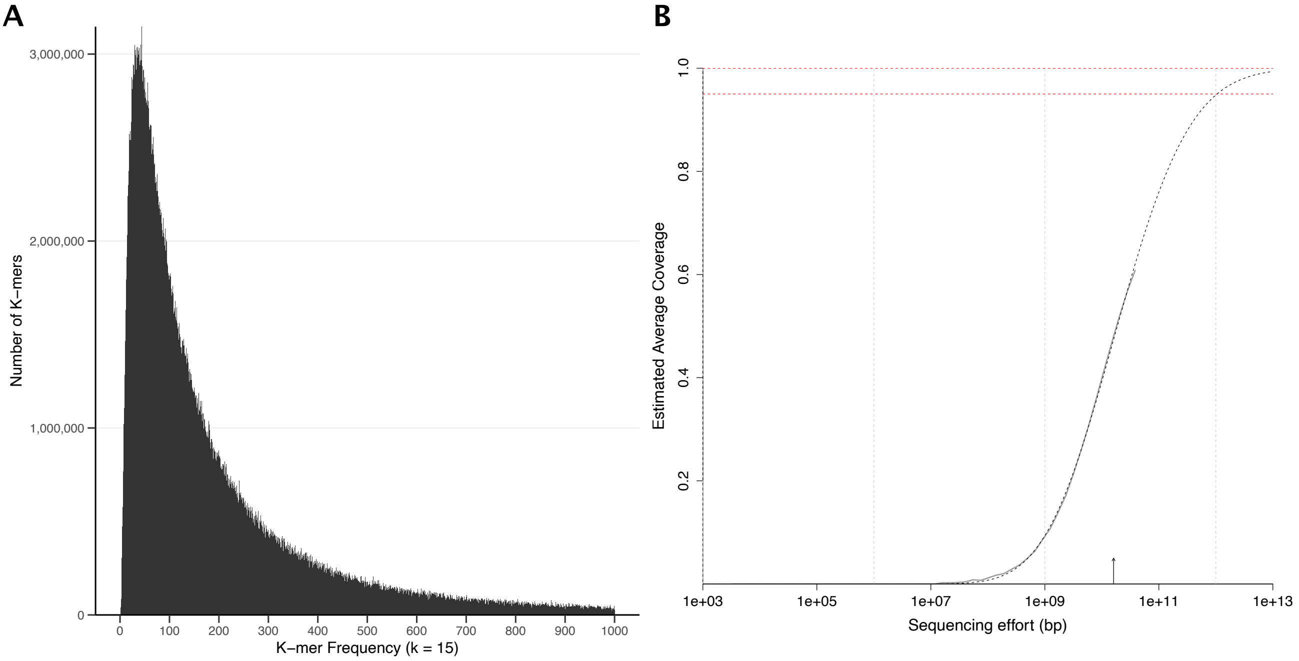

Figure 2. Sequencing depth diagnostics show that ultra-deep sequencing still undersamples the total soil diversity. **(A)** 15-mer spectrum of Nanopore reads from the soil metagenome. Bars show the number of distinct 15-mers observed at each copy number. The pronounced peak at low frequencies reflects a very large pool of unique or low-coverage k-mers, indicating extreme sequence diversity. The long right-hand tail originates from higher coverage k-mers, likely derived from abundant taxa and repetitive genomic regions. **(B)** Nonpareil analysis estimating the sequencing coverage of the pooled Illumina dataset. The curve shows the relationship between sequencing effort and estimated coverage, with the solid line representing the observed dataset and the dashed extrapolation indicating expected gains with additional sequencing. The analysis suggests that substantially more sequencing effort would be required to achieve near-complete coverage of the community.

at the species level, where the rarefaction curves continued to rise linearly across all subsampling depths. This indicates an exceptional degree of fine-scale taxonomic resolution, with many closely related but genomically distinct lineages present at low abundance. Similarly, functional clustering of coding sequences revealed a parallel pattern: at high identity thresholds, the number of distinct CDS clusters continued to grow steeply with sequencing depth, while at lower thresholds, sequences clustered more readily, reflecting both the breadth of novel gene diversity and underlying functional redundancy across lineages.

Such strain- and species-level diversity is particularly important given that many ecologically and functionally relevant traits, such as niche specialisation, symbiosis, and secondary metabolite biosynthesis, can differ even between closely related strains. In more constrained environments, such as the human gut, species-level diversity tends to plateau after a few hundred genomes [68], and marine planktonic communities exhibit relatively stable core microbiomes [69]. By contrast, the soil microbiome appears to exhibit near-limitless diversity at the genomic level.

These findings not only demonstrate the limitations of current sequencing depths but also call into question the accuracy of historical species richness estimates derived from much shallower data. They suggest that achieving anything approaching species-level saturation in soil will likely require terabase- to petabase-scale sequencing efforts, combined with refined computational methods to resolve low-abundance and highly diverse taxa. They demonstrate that many of the diversity estimates historically used in soil microbiome research, often based on much shallower sequencing, likely underestimate the true scale of taxonomic complexity.

Long-read metagenomics recovers thousands of novel and genome-linked BGCs

From the hybrid metagenomic assembly, we identified a total of 11 381 BGCs, including 5652 classified as complete, reflecting the improved contiguity made possible through long-read sequencing. This proportion of complete clusters (~50%) represents a substantial improvement over previous metagenomic surveys, where fewer than 10% of BGCs are typically annotated as complete [48].

Clustering the identified BGCs with BiG-SCAPE resulted in 10 215 gene cluster families, indicating that most BGCs in our dataset are non-redundant and functionally distinct. The size distribution of GCFs (Figure 4A) was highly skewed: most families consisted of singletons or doubletons, with only a handful of larger families, further illustrating the functional uniqueness and fine-grained metabolic specialisation within the community. The largest GCF contains 115 ribosomally synthesised and post-transcriptionally modified peptide (RiPP) BGCs. The cumulative ordering of the GCFs by size shows that the largest eight families account for 3.1% of all BGCs, whereas each of the remaining over ten thousand families contributes only very little to the total number (Figure 4B).

Taxonomic classification of the BGC-containing contigs revealed that biosynthetic potential is broadly distributed across the phylogenetic spectrum. While well-known producers such as Actinomycetota and Pseudomonadota were represented, we also detected numerous BGCs in less-studied phyla such as Verrucomicrobiota and Acidobacteriota (Figure 4C). These groups have historically been overlooked due to cultivation barriers but appear to harbour substantial secondary metabolic capacity.

Only 108 BGCs (~ 1%) had significant matches to known GCFs in the BGC Atlas database, which compiles nearly two million BGCs from metagenomic sources, and none clustered together with reference BGCs from MIBiG. This confirms earlier observations that soil metagenomes are rich in biosynthetic novelty [15, 70], but also demonstrates that even with massive reference expansions, a typical soil still contains an overwhelming majority of uncharacterised BGCs. Notably, our dataset contains more novel BGCs than reported in most large-scale studies of marine or human microbiomes, where the rate of novel BGC discovery is often constrained by lower microbial diversity and shorter contigs [70, 48].

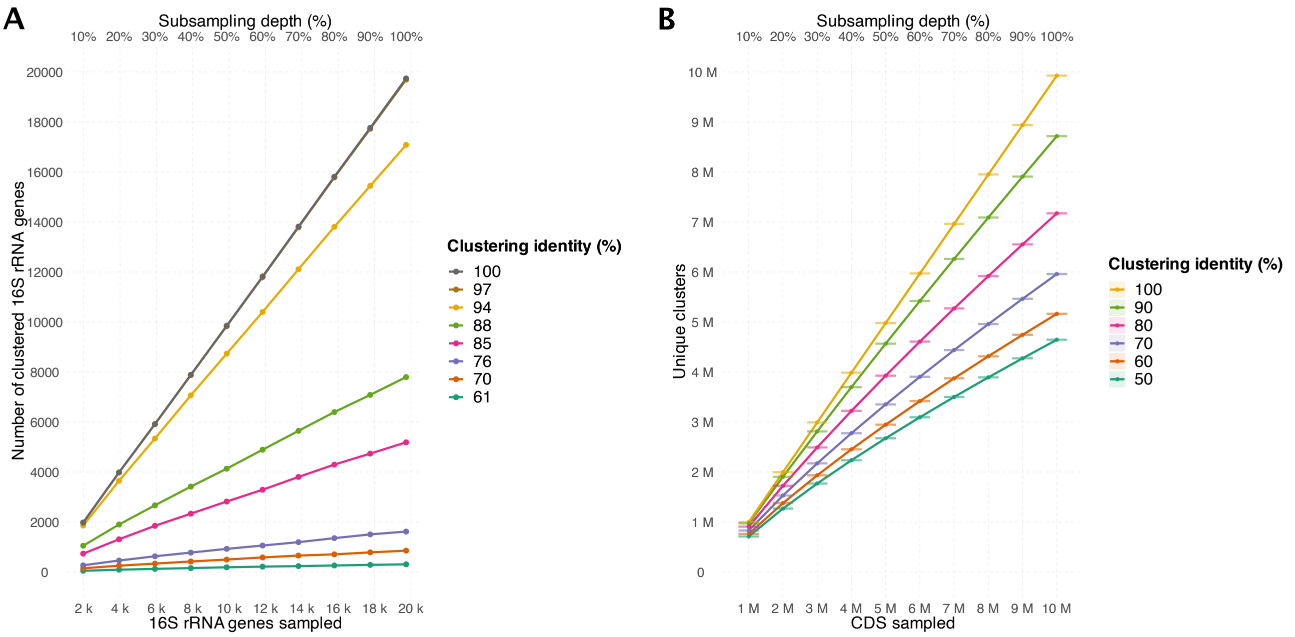

Figure 3. Rarefaction curves of full-length 16S rRNA gene sequences extracted from Nanopore reads (A) and CDSs (B) at different clustering thresholds. **(A)** The number of observed full-length 16S rRNA gene sequences is plotted against the number of sampled genes at clustering thresholds ranging from 61% to 100% sequence identity. The curves at high-identity thresholds, such as 97% and 100%, increase in a near-linear fashion, indicating that the vast majority of taxonomic diversity remains undiscovered, while the increase is less steep at lower clustering thresholds. **(B)** Rarefaction curves of predicted coding sequences (CDSs) from the final assembly, clustered at identity thresholds from 50% to 100%, with a bi-directional coverage requirement of 80%. The number of unique protein clusters continues to rise steeply even after sampling 10 million CDSs, demonstrating that the functional gene repertoire of the soil community is also far from saturated.

Hybrid assembly recovers hundreds of genomes with high taxonomic and biosynthetic novelty

From the hybrid assembly, we recovered 837 metagenome-assembled genomes (MAGs), of which 466 met the MIMAG standards for high- or medium-quality [71]. High- and medium-quality MAGs ranged in size from 1 Mbp (Patescibacteria) to 15 Mbp (Planctomycetota) (Figure 5 and Supplementary Table S1). All MAGs recovered represented unique species-level units, defined by > 0.05 pairwise Mash distance ($\geq$ 95% ANI). These genomes spanned a wide range of bacterial phyla typical of soils, including Acidobacteriota, Pseudomonadota, Verrucomicrobiota, Actinomycetota, and Myxococcota. Remarkably, only four of the 837 MAGs had species-level matches ($\geq$95% ANI) in the GTDB database, and none had cultured representatives.

Thirteen MAGs were assigned to phyla with no cultured members, such as Desulfobacterota, and candidate phyla radiation (CPR) groups FCPU426 and JAJYCY01, harbouring between 2 and 19 BGCs (Figure 5). At other taxonomic levels, 9, 45, 155, and 611 MAGs belong to novel lineages that correspond to class, order, family, and genus levels, respectively.

Many of the recovered MAGs are assigned to taxonomic groups that do not correspond to any known families or even classes in current reference databases (Figure 5). This points to a substantial fraction of microbial diversity in soil that remains undescribed, even at higher taxonomic ranks. In addition to their taxonomic novelty, many of these genomes contain a large number of BGCs, in some cases reaching 30 distinct clusters within a single genome. This suggests that the potential for secondary metabolite production is not restricted to well-characterised soil taxa (such as Actinomycetota), but is also widespread among poorly characterised lineages.

Of all BGCs identified, 6279 could be linked to metagenome-assembled genomes, enabling genome-resolved exploration of natural product potential. This represents one of the largest genome-resolved inventories of BGCs from a single environmental sample to date.

The ability to recover thousands of complete and genome-linked BGCs from a single soil metagenome demonstrates the power of ultra-deep long-read sequencing in capturing microbial metabolic potential. In addition, the high novelty rate and wide taxonomic distribution of these clusters emphasise a vast and largely untapped chemical repertoire unlikely to be captured through shallow sequencing or cultivation-based surveys alone.

Discussion

Soil diversity still outruns ultra-deep sequencing

Our study presents one of the most comprehensive metagenomic investigations of a single soil microbiome to date, combining ultra-deep Oxford Nanopore and Illumina sequencing to yield nearly 300 Gbp of data. This effort enabled high-quality hybrid assemblies and genome-resolved analysis of taxonomic and functional potential in a temperate forest soil sample, providing an empirical benchmark for the capabilities and current limitations of metagenomic sequencing in one of the most complex microbial habitats on Earth.

Despite the ultra-deep sequencing depth, our analyses demonstrate that the microbial diversity of the sample remains far from saturated. Rarefaction curves, k-mer profiles, 16S rRNA gene and marker gene analyses, and functional clustering of CDSs consistently indicate that substantially more sequencing would be required to approach near-complete coverage of the community diversity, with a non-parametric model estimate on the order of ~ 10 Tbp of raw sequencing data to reach high-coverage targets (Figure 2). By comparison, MAG recovery in human-gut communities reaches saturation with ~ 5–10 Gbp per sample, and 25 Gbp is estimated to be enough even to classify taxa of very low abundances (< 1e-06) [72]. Similarly, Ni et al. [73] estimate this number to be around 7

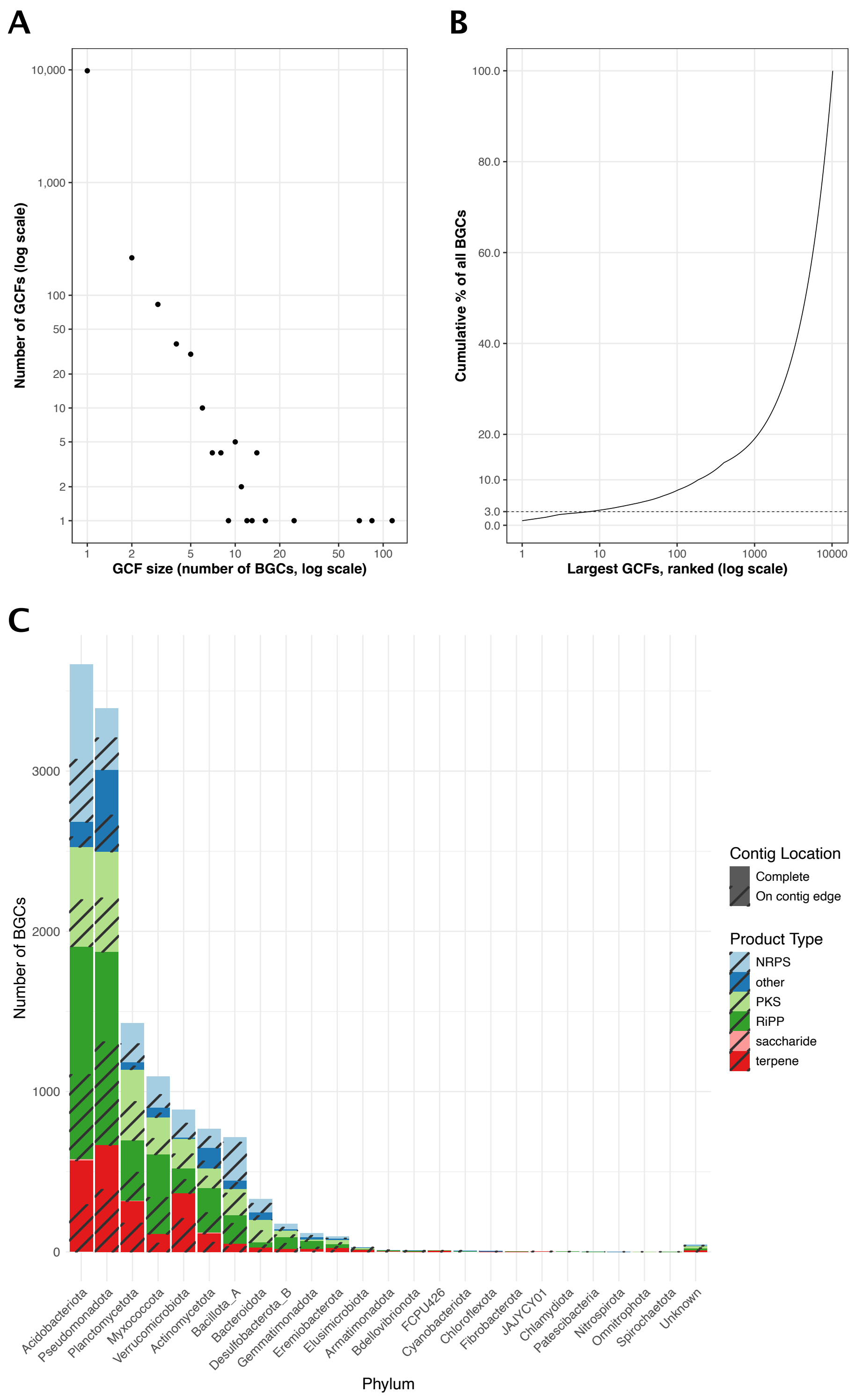

Figure 4. The diversity and taxonomic composition of the biosynthetic gene clusters. **(A)** The size distribution of gene cluster families (GCFs). Each dot represents one distinct GCF size in the dataset (11 381 biosynthetic gene clusters grouped into 10 215 GCFs). The x-axis shows the family size (number of BGCs), the y-axis shows the number of families of that size; both axes are $\log_{10}$-scaled. The plot reveals a heavy tail: 9813 families (96.1 %) are singletons, only 20 families contain $\geq$10 BGCs, and the largest family comprises 115 BGCs. **(B)** Cumulative contribution of the largest GCFs to the total BGC dataset. Families are ranked from largest to smallest along the $\log_{10}$-scaled x-axis; the y-axis tracks the running percentage of all 11 381 BGCs accounted for as each additional family is added. The eight largest GCFs explain 3.1% of all BGCs, whereas 7939 families are required to reach 80%. The rapid rise followed by a very shallow tail shows that a small handful of families contribute to about 3% of BGCs in the dataset, while thousands of minor families are unique. **(C)** Distribution of biosynthetic gene cluster (BGC) product types across bacterial phyla. Each bar represents the total number of BGCs assigned to a given phylum, coloured by predicted product type. Striped regions indicate the proportion of BGCs classified as "on-contig-edge" by antiSMASH. Phyla are ordered by total BGC count, with "Unknown" denoting contigs for which no confident phylum-level classification could be assigned.

Gbp to detect gene content of organisms with relative abundances over 1% in human faecal samples.

Ultra-deep campaigns such as Tara Oceans have shown that planktonic marine communities also benefit from multi-terabase sequencing, yielding thousands of novel genes and genomes and greatly refining oceanic reference catalogues. Yet, the incremental discovery of truly new sequence space in the ocean has begun to flatten at these depths [69, 74, 75]. In soils, by contrast, every addi-

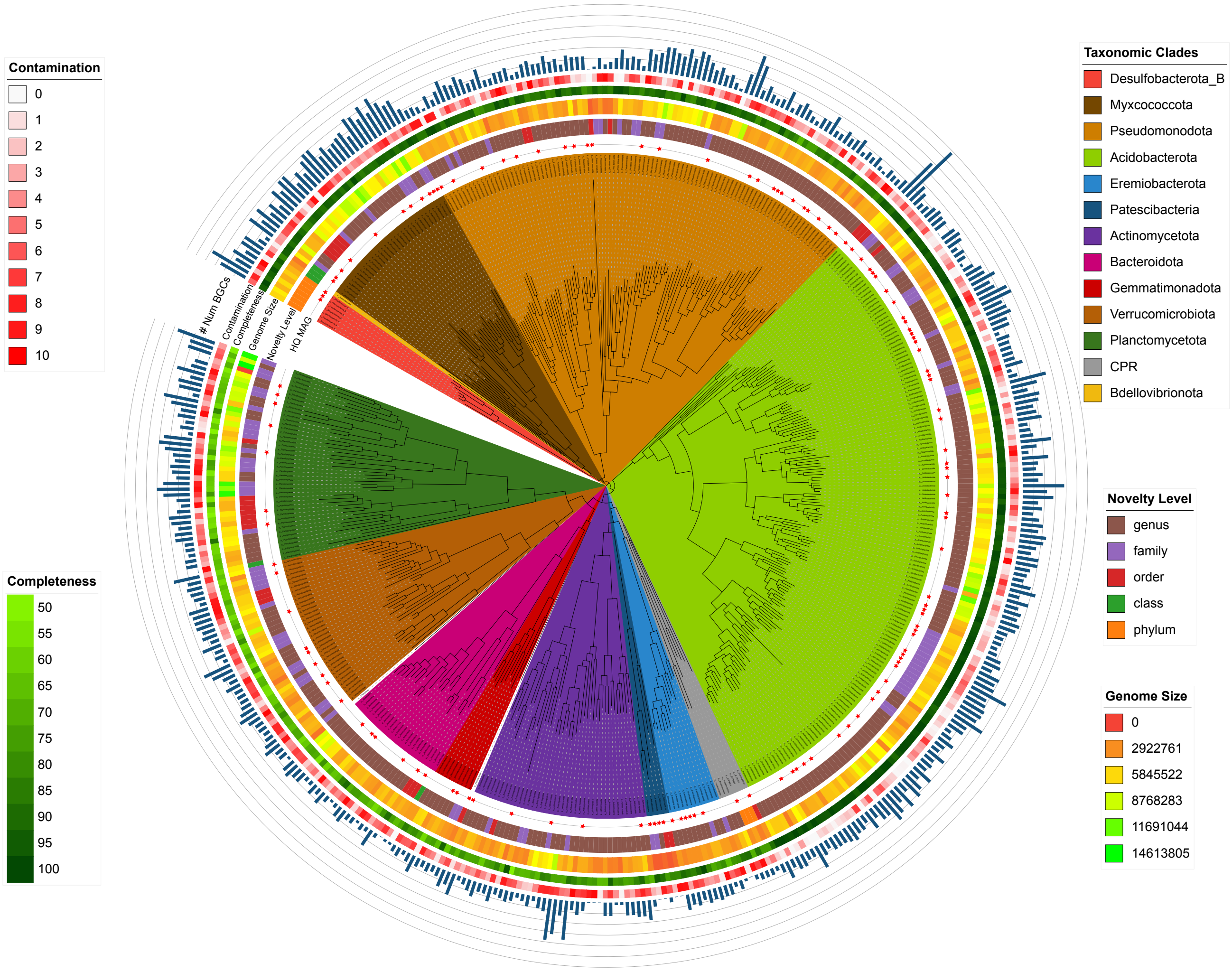

Figure 5. Genome quality statistics and taxonomic classification of newly reconstructed high-quality and medium-quality metagenome-assembled genomes (MAGs). Each leaf represents a MAG, coloured by its assigned phylum. Concentric rings around the leaves indicate, from innermost to outermost: (i) HQ MAGs, (ii) novelty level (genus to phylum), (iii) genome size (bp), (iv) completeness (%), (v) contamination (%), and (vi) the number of BGCs encoded by the MAG (range: 0-30).

tional sequencing effort still brings a steeper growth of previously unseen sequence diversity, and MAGs, with no hint of saturation.

Because our analysis derives from a single temperate Cambisol soil sample, these results should be read as a depth benchmark rather than a census of soils at a global scale. We expect the heavy-tailed diversity and lack of saturation to generalise to other complex soils; however, the taxonomic composition and biosynthetic potential may vary with soil type, season, agricultural use, and other factors.

During the revision of this manuscript, three independent studies using broader sampling and alternative designs reached similar conclusions, placing our single-site depth benchmark in a broader context. A multi-habitat long-read resource sequenced 154 Danish soils with an emphasis on expanding reference space across habitats, and recovered more than 15 000 previously undescribed species and thousands of complete BGCs, demonstrating how long reads broadly improve genome recovery and downstream annotation across terrestrial microbiomes [76]. They also demonstrated a near-linear rarefaction relationship between the number of MAGs that are recovered from the soil samples with the number of species they correspond to. Complementarily, another analysis of publicly available short-read sequencing efforts of soil samples estimated that multi-terabase sequencing efforts per sample may be needed to approach near-complete saturation, consistent with our observation that soil diversity remains unsaturated at current depths [77]. They also demonstrate that large co-assembly efforts of short-read soil metagenomics improve the coverage, yet still fall short of capturing it completely. Additionally, Burian et. al. [78] introduced the first terabase-scale long-read sequencing effort of a single soil sample, focusing on the recovery of nonribosomal peptide (NRPS) BGCs. They generated 2.5 Tbp of raw long-read data from one forest soil, where the NRPS discovery scaled linearly up to the 2.5 of the generated data, not capturing the full diversity of NRPS BGCs in the single soil sample.

Uncharted novelty of soil microbes and their functional capabilities

Taxonomic profiling of assembled contigs revealed the presence of a diverse range of phyla in the soil, including the expected dominance of well-known soil phyla, such as Acidobacteriota, Actinomycetota, and Pseudomonadota, alongside under-studied lineages. Yet, over 95% of contigs could not be assigned to a species currently present in databases, and less than 0.7% could be linked to a species with at least one cultured representative. We recovered MAGs that represent candidate novel phyla, highlighting the ability of genome-resolved metagenomics to reveal deeply divergent lineages missed by conventional approaches. Only four MAGs recovered in this study share $\geq$ 95% ANI with genomes in GTDB, and none correspond to cultured isolates, reaffirming how incompletely current databases represent soil ecosystems. These lineages

not only expand the bacterial tree of life but, in many cases, also harbour unique biosynthetic gene clusters, suggesting functional capacities that remain entirely uncharted.

The scale of biosynthetic diversity uncovered is similarly striking. We identified over 11 000 BGCs, more than 5600 of which are complete and many of which could be linked to MAGs. Fewer than 1% of these BGCs matched known families in public databases, suggesting that soils remain a massive and underexplored reservoir for natural product discovery. In other words, the chemistry encoded in a single forest soil sample still sits almost entirely outside the reach of current reference catalogues. The heavy-tailed GCF distribution we observe, where more than 96% of families are singletons, suggests that every additional increment of sequencing continues to reveal functionally distinct BGCs, rather than finding copies of known ones.

These BGCs come from virtually every major bacterial phylum detected in the sample, from well-studied Actinomycetota and Pseudomonadota to under-explored Acidobacteriota, Verrucomicrobiota, and candidate phyla radiation groups. This phylogenetic breadth and the underestimated functional diversity of soil microbes indicate that the soil's metabolic repertoire is not restricted to a few prolific lineages but is dispersed across the microbial tree of life, with implications for microbial interactions, nutrient cycling, defensive traits, and overall ecosystem stability.

Furthermore, our dataset shows that long-read sequencing can address common limitations of metagenomics approaches, including incomplete recovery of BGCs and fragmented assemblies, thereby enabling genome-resolved assessments of secondary metabolism. In contrast to earlier studies depending exclusively on short-read data [48], our approach yields a high proportion of complete BGCs and facilitates the association of biosynthetic capacity with uncultured taxa.

Conclusions

Together, these results redefine the scale of the unknown in soil microbiomes. They demonstrate that even the deepest sequencing efforts to date barely scratch the surface of microbial and metabolic diversity, particularly in complex environments. Moving forward, terabase- to petabase-scale sequencing will likely be necessary to achieve species-level saturation in soil. However, deeper sequencing alone will not be sufficient. Advances in bioinformatics for assembly, binning, and functional annotation, combined with complementary data from metatranscriptomics, metabolomics, and single-cell approaches, will be essential for translating sequence data into ecological and biochemical insight.

In conclusion, this study sets a new reference point for what is currently achievable in soil metagenomics. By combining ultra-deep sequencing with advanced computational methods, we provide an updated, data-driven view of soil microbial and biosynthetic diversity. The dataset offers an empirical baseline for evaluating sequencing depth, genome recovery, and biosynthetic novelty in complex environments. As such, it can serve as a valuable resource for guiding future studies, benchmarking new tools, and refining diversity estimates. Our findings highlight both the power of current technologies and the need for continued investment in sequencing and analysis to uncover the vast, largely uncharacterised microbial life in soil ecosystems.

Potential implications

Beyond its soil-ecological focus, this ultra-deep, genome-resolved dataset can (i) act as a benchmark for soil diversity in future studies. (ii) It supplies hundreds of genomes from previously unseen lineages to extend genome databases and taxonomies such as MGnify [79, 80] and GTDB [29], and refine the bacterial tree of life. (iii) It contains >11 000 largely novel and complete BGCs, many linked to host genomes, which can extend the secondary metabolite databases such as BGC Atlas [48] and the Secondary Metabolism Collaboratory (SMC) [81]; (iv) and this phylogenetically diverse and novel collection of genomes and BGCs can be used as a training set for machine-learning models that predict compound functions [82] and structures [83], and the ecological traits of microbes [84].

Availability of source code and requirements

- Project name: Soil Metagenome Pipeline
- Project home page: https://github.com/ZiemertLab/soil-metagenome-pipeline
- WorkflowHub SEEK ID: https://workflowhub.eu/workflows/1960?version=1
- License: GNU General Public License v3.0

System requirements

- Operating system(s): Linux
- Programming language: Nextflow DSL2, Python, bash
- Other requirements: conda, SLURM
- Hardware requirements: >64 threads, >128GB RAM

Data availability

The raw sequencing data generated during the current study are available in the ENA repository [1]. The identified biosynthetic gene clusters (BGCs), and extracted MAGs are available via Zenodo for unrestricted reuse [2, 3]. The computational analysis pipeline has been made available on GitHub [85] with the GNU General Public License v3.0 and deposited on workflowhub.eu [65] as a NextFlow pipeline.

Additional Files

Supplementary Fig. S1. Distribution of predicted proteins into COG categories.

Supplementary Fig. S2. 17-mer spectrum of Illumina reads.

Supplementary Fig. S3. 17-mer spectrum of Nanopore reads

Supplementary Fig. S4. Length distribution of the 16S rRNA gene sequences extracted from raw Nanopore reads.

Supplementary Fig. S5. The number of occurrences of each marker gene from bac120 set in raw Nanopore reads.

Supplementary Table S1. The details of the extracted MAGs, including their completeness and contamination values, taxonomic classification, assembly statistics, genome annotations statistics, and the number of BGCs identified.

Declarations

List of abbreviations

ANI: average nucleotide identity; BGC: biosynthetic gene cluster; bp: base pairs; CDS: coding sequence; CPR: Candidate phyla radiation; DNA: deoxyribonucleic acid; ENA: European Nucleotide Archive; Gbp: giga (billion) base pairs; GCF: gene cluster family; GTDB: Genome Taxonomy Database; HQ: high quality; MAG: metagenome-assembled genome; MIBiG: Minimum Information about a Biosynthetic Gene Cluster; MIMAG: Minimum Information

about a Metagenome-Assembled Genome; MQ: medium quality; NGS: next-generation sequencing; NRPS: non-ribosomal peptide synthetase; kbp: kilo (thousand) base pairs; Mbp: mega (million) base pairs; ONT: Oxford Nanopore Technologies; PKS: polyketide synthase; Q-score: quality score (Phred); RiPP: ribosomally synthesised and post-transcriptionally modified peptide

Ethical Approval

Not applicable

Consent for publication

Not applicable

Competing Interests

The authors declare that they have no competing interests.

Funding

This work was supported by Bundesministerium für Bildung und Forschung (BMBF), MicroMatrix Project, 161L0284C to N.Z., and Deutsches Zentrum für Infektionsforschung (DZIF), Applied Natural Products for Genome Mining, TTU09.716 to N.Z.

Authors' Contributions

C.B. and N.Z. wrote the main manuscript. C.B. performed the data analysis and prepared the figures. T.N. conducted the soil sampling and DNA isolation. E.A.B. carried out the Nanopore sequencing. C.G. performed basecalling and preliminary quality control of the sequencing data. C.B., S.O., and N.Z. conceptualised the study. All authors reviewed the manuscript.

Acknowledgements

The authors acknowledge the support by the High Performance and Cloud Computing Group at the Zentrum für Datenverarbeitung of the University of Tübingen and the Federal Ministry of Education and Research (BMBF) through grant no. 031 A535A. We thank the Interfaculty Institute for Biomedical Informatics (IBMI) at the University of Tübingen for providing the computational resources essential for this study. NGS sequencing methods were performed with the support of the DFG-funded NGS Competence Center Tübingen (INST 37/1049-1). Additionally, we thank the Deutsche Forschungsgemeinschaft (DFG, German Research Foundation) under Germany's Excellence Strategy—EXC 2124—390838134 for structural support.

References

1. Bağcı C, Negri T, Buena-Atienza E, Gross C, Ossowski S, Ziemert N, BioProject PRJEB89893 [Dataset]. ENA; 2025. https://www.ebi.ac.uk/ena/browser/view/PRJEB89893.
2. Bağcı C, Negri T, Buena-Atienza E, Gross C, Ossowski S, Ziemert N, MAGs and their antiSMASH annotations from an ultra-deep sequencing effort of a forest sample in Schönbuch, Germany [Dataset]. Zenodo; 2025. https://doi.org/10.5281/zenodo.15866786.
3. Bağcı C, Negri T, Buena-Atienza E, Gross C, Ossowski S, Ziemert N, antiSMASH and eggNOG mapper annotations from an ultra-deep sequencing effort of a forest sample in Schönbuch, Germany [Dataset]. Zenodo; 2025. https://doi.org/10.5281/zenodo.17162541.
4. Fierer N. Embracing the unknown: disentangling the complexities of the soil microbiome. Nature Reviews Microbiology 2017;15(10):579–590. https://doi.org/10.1038/nrmicro.2017.87.
5. Roesch LF, Fulthorpe RR, Riva A, Casella G, Hadwin AK, Kent AD, et al. Pyrosequencing enumerates and contrasts soil microbial diversity. The ISME Journal 2007;1(4):283–290. https://doi.org/10.1038/ismej.2007.53.
6. Torsvik V, Øvreås L. Microbial diversity and function in soil: from genes to ecosystems. Current Opinion in Microbiology 2002;5(3):240–245. https://doi.org/10.1016/S1369-5274(02)00324-7.
7. Jousset A, Bienhold C, Chatzinotas A, Gallien L, Gobet A, Kurm V, et al. Where less may be more: how the rare biosphere pulls ecosystems strings. The ISME Journal 2017;11(4):853–862. https://doi.org/10.1038/ismej.2016.174.
8. Lok C. Mining the microbial dark matter. Nature 2015;522(7556):270. https://doi.org/10.1038/522270a.
9. Hug LA, Baker BJ, Anantharaman K, Brown CT, Probst AJ, Castelle CJ, et al. A new view of the tree of life. Nature Microbiology 2016;1(5):1–6. https://doi.org/10.1038/nmicrobiol.2016.48.
10. Lloyd KG, Steen AD, Ladau J, Yin J, Crosby L. Phylogenetically novel uncultured microbial cells dominate earth microbiomes. mSystems 2018;3(5):e00055–18. https://doi.org/10.1128/mSystems.00055-18.
11. Nascimento Lemos L, Manoharan L, William Mendes L, Monteiro Venturini A, Satler Pylro V, Tsai SM. Metagenome assembled-genomes reveal similar functional profiles of CPR/Patescibacteria phyla in soils. Environmental Microbiology Reports 2020;12(6):651–655. https://doi.org/10.1111/1758-2229.12880.
12. Nayfach S, Roux S, Seshadri R, Udwary D, Varghese N, Schulz F, et al. A genomic catalog of Earth's microbiomes. Nature Biotechnology 2021;39(4):499–509. https://doi.org/10.1038/s41587-020-0718-6.
13. Parks DH, Rinke C, Chuvochina M, Chaumeil PA, Woodcroft BJ, Evans PN, et al. Recovery of nearly 8,000 metagenome-assembled genomes substantially expands the tree of life. Nature Microbiology 2017;2(11):1533–1542. https://doi.org/10.1038/s41564-017-0012-7.
14. Bertrand D, Shaw J, Kalathiyappan M, Ng AHQ, Kumar MS, Li C, et al. Hybrid metagenomic assembly enables high-resolution analysis of resistance determinants and mobile elements in human microbiomes. Nature Biotechnology 2019;37(8):937–944. https://doi.org/10.1038/s41587-019-0191-2.
15. Van Goethem MW, Osborn AR, Bowen BP, Andeer PF, Swenson TL, Clum A, et al. Long-read metagenomics of soil communities reveals phylum-specific secondary metabolite dynamics. Communications Biology 2021;4(1):1302. https://doi.org/10.1038/s42003-021-02809-4.
16. Agustinho DP, Fu Y, Menon VK, Metcalf GA, Treangen TJ, Sedlazeck FJ. Unveiling microbial diversity: harnessing long-read sequencing technology. Nature Methods 2024;21(6):954–966. https://doi.org/10.1038/s41592-024-02262-1.
17. Wick RR, Judd LM, Holt KE. Assembling the perfect bacterial genome using Oxford Nanopore and Illumina sequencing. PLOS Computational Biology 2023;19(3):e1010905. https://doi.org/10.1371/journal.pcbi.1010905.
18. Zhang T, Li H, Jiang M, Hou H, Gao Y, Li Y, et al. Nanopore sequencing: flourishing in its teenage years. Journal of Genetics and Genomics 2024;https://doi.org/10.1016/j.jgg.2024.09.007.
19. Mantri SS, Negri T, Sales-Ortells H, Angelov A, Peter S, Neidhardt H, et al. Metagenomic sequencing of multiple soil horizons and sites in close vicinity revealed novel secondary

metabolite diversity. mSystems 2021;6(5):e01018–21. https://doi.org/10.1128/msystems.01018-21.
20. Negri T, Mantri S, Angelov A, Peter S, Muth G, Eustáquio AS, et al. A rapid and efficient strategy to identify and recover biosynthetic gene clusters from soil metagenomes. Applied Microbiology and Biotechnology 2022;106(8):3293–3306. https://doi.org/10.1007/s00253-022-11917-y.
21. Chen S. Ultrafast one-pass FASTQ data preprocessing, quality control, and deduplication using fastp. iMeta 2023;2(2):e107. https://doi.org/10.1002/imt2.107.
22. Kolmogorov M, Bickhart DM, Behsaz B, Gurevich A, Rayko M, Shin SB, et al. metaFlye: scalable long-read metagenome assembly using repeat graphs. Nature Methods 2020;17(11):1103–1110. https://doi.org/10.1038/s41592-020-00971-x.
23. Oxford Nanopore Technologies, GitHub: medaka (Version 2.0.1). GitHub; 2025. https://github.com/nanoporetech/medaka/releases/tag/v2.0.1.
24. Hu J, Fan J, Sun Z, Liu S. NextPolish: a fast and efficient genome polishing tool for long-read assembly. Bioinformatics 2020;36(7):2253–2255. https://doi.org/10.1093/bioinformatics/btz891.
25. Li H, Durbin R. Fast and accurate short read alignment with Burrows–Wheeler transform. Bioinformatics 2009;25(14):1754–1760. https://doi.org/10.1093/bioinformatics/btp324.
26. Li H. Minimap2: pairwise alignment for nucleotide sequences. Bioinformatics 2018;34(18):3094–3100. https://doi.org/10.1093/bioinformatics/bty191.
27. Li D, Liu CM, Luo R, Sadakane K, Lam TW. MEGAHIT: an ultra-fast single-node solution for large and complex metagenomics assembly via succinct de Bruijn graph. Bioinformatics 2015;31(10):1674–1676. https://doi.org/10.1093/bioinformatics/btv033.
28. Kim J, Steinegger M. Metabuli: sensitive and specific metagenomic classification via joint analysis of amino acid and DNA. Nature Methods 2024;21(6):971–973. https://doi.org/10.1038/s41592-024-02273-y.
29. Parks DH, Chuvochina M, Rinke C, Mussig AJ, Chaumeil PA, Hugenholtz P. GTDB: an ongoing census of bacterial and archaeal diversity through a phylogenetically consistent, rank normalized and complete genome-based taxonomy. Nucleic Acids Research 2022;50(D1):D785–D794. https://doi.org/10.1093/nar/gkab776.
30. Rhie A, Nurk S, Cechova M, Hoyt SJ, Taylor DJ, Altemose N, et al. The complete sequence of a human Y chromosome. Nature 2023;621(7978):344–354. https://doi.org/10.1038/s41586-023-06457-y.
31. Steinegger M, Söding J. MMseqs2 enables sensitive protein sequence searching for the analysis of massive data sets. Nature Biotechnology 2017;35(11):1026–1028. https://doi.org/10.1038/nbt.3988.
32. Mohamadi H, Khan H, Birol I. ntCard: a streaming algorithm for cardinality estimation in genomics data. Bioinformatics 2017;33(9):1324–1330. https://doi.org/10.1093/bioinformatics/btw832.
33. Rodriguez-R LM, Gunturu S, Tiedje JM, Cole JR, Konstantinidis KT. Nonpareil 3: fast estimation of metagenomic coverage and sequence diversity. mSystems 2018;3(3):e00039–18. https://doi.org/10.1128/msystems.00039-18.
34. Seemann T, GitHub: Barrnap (Version 0.9). GitHub; 2025. https://github.com/tseemann/barrnap/releases/tag/0.9.
35. Rognes T, Flouri T, Nichols B, Quince C, Mahé F. VSEARCH: a versatile open source tool for metagenomics. PeerJ 2016;4:e2584. https://doi.org/10.7717/peerj.2584.
36. Schwengers O, Jelonek L, Dieckmann MA, Beyvers S, Blom J, Goesmann A. Bakta: rapid and standardized annotation of bacterial genomes via alignment-free sequence identification. Microbial Genomics 2021;7(11):000685. https://doi.org/10.1099/mgen.0.000685.
37. Galperin MY, Vera Alvarez R, Karamycheva S, Makarova KS, Wolf Y, Landsman D, et al. COG database update 2024. Nucleic Acids Research 2024 11;53(D1):D356–D363. https://doi.org/10.1093/nar/gkae983.
38. Hernández-Plaza A, Szklarczyk D, Botas J, Cantalapiedra CP, Giner-Lamia J, Mende DR, et al. eggNOG 6.0: enabling comparative genomics across 12 535 organisms. Nucleic Acids Research 2023;51(D1):D389–D394. https://doi.org/10.1093/nar/gkac1022.
39. Kanehisa M, Furumichi M, Tanabe M, Sato Y, Morishima K. KEGG: new perspectives on genomes, pathways, diseases and drugs. Nucleic Acids Research 2017;45(D1):D353–D361. https://doi.org/10.1093/nar/gkw1092.
40. The Gene Ontology Consortium SA Aleksander, Balhoff J, Carbon S, Cherry JM, Drabkin HJ, Ebert D, et al. The Gene Ontology knowledgebase in 2023. Genetics 2023;224(1):iyad031. https://doi.org/10.1093/genetics/iyad031.
41. Cantarel BL, Coutinho PM, Rancurel C, Bernard T, Lombard V, Henrissat B. The Carbohydrate-Active EnZymes database (CAZy): an expert resource for Glycogenomics. Nucleic Acids Research 2009;37(suppl_1):D233–D238. https://doi.org/10.1093/nar/gkn663.
42. Mistry J, Chuguransky S, Williams L, Qureshi M, Salazar GA, Sonnhammer EL, et al. Pfam: The protein families database in 2021. Nucleic Acids Research 2021;49(D1):D412–D419. https://doi.org/10.1093/nar/gkaa913.
43. Cantalapiedra CP, Hernández-Plaza A, Letunic I, Bork P, Huerta-Cepas J. eggNOG-mapper v2: functional annotation, orthology assignments, and domain prediction at the metagenomic scale. Molecular Biology and Evolution 2021;38(12):5825–5829. https://doi.org/10.1093/molbev/msab293.
44. Steinegger M, Söding J. Clustering huge protein sequence sets in linear time. Nature Communications 2018;9(1):2542. https://doi.org/10.1038/s41467-018-04964-5.
45. Blin K, Shaw S, Augustijn HE, Reitz ZL, Biermann F, Alanjary M, et al. antiSMASH 7.0: new and improved predictions for detection, regulation, chemical structures and visualisation. Nucleic Acids Research 2023;51(W1):W46–W50. https://doi.org/10.1093/nar/gkad344.
46. Draisma A, Loureiro C, Louwen NL, Kautsar SA, Navarro-Muñoz JC, Doering DT, et al. BiG-SCAPE 2.0 and BiG-SLiCE 2.0: scalable, accurate and interactive sequence clustering of metabolic gene clusters. bioRxiv 2025;https://doi.org/10.1101/2025.08.20.671210.
47. Zdouc MM, Blin K, Louwen NL, Navarro J, Loureiro C, Bader CD, et al. MIBiG 4.0: advancing biosynthetic gene cluster curation through global collaboration. Nucleic Acids Research 2025;53(D1):D678–D690. https://doi.org/10.1093/nar/gkae1115.
48. Bağcı C, Nuhamunada M, Goyat H, Ladanyi C, Sehnal L, Blin K, et al. BGC Atlas: a web resource for exploring the global chemical diversity encoded in bacterial genomes. Nucleic Acids Research 2025;53(D1):D618–D624. https://doi.org/10.1093/nar/gkae953.
49. Kautsar SA, van der Hooft JJ, de Ridder D, Medema MH. BiG-SLiCE: A highly scalable tool maps the diversity of 1.2 million biosynthetic gene clusters. GigaScience 2021;10(1):giaa154. https://doi.org/10.1093/gigascience/giaa154.
50. Wang Z, You R, Han H, Liu W, Sun F, Zhu S. Effective binning of metagenomic contigs using contrastive multi-view representation learning. Nature Communications 2024;15(1):585. https://doi.org/10.1038/s41467-023-44290-z.
51. Liu CC, Dong SS, Chen JB, Wang C, Ning P, Guo Y, et al. MetaDecoder: a novel method for clustering metagenomic contigs. Microbiome 2022;10(1):46. https://doi.org/10.1186/s40168-022-01237-8.

52. Pan S, Zhao XM, Coelho LP. SemiBin2: self-supervised contrastive learning leads to better MAGs for short-and long-read sequencing. Bioinformatics 2023;39(Supplement_1):i21–i29. https://doi.org/10.1093/bioinformatics/btad209.
53. Nissen JN, Johansen J, Allesøe RL, Sønderby CK, Armenteros JJA, Grønbech CH, et al. Improved metagenome binning and assembly using deep variational autoencoders. Nature Biotechnology 2021;39(5):555–560. https://doi.org/10.1038/s41587-020-00777-4.
54. Parks DH, Imelfort M, Skennerton CT, Hugenholtz P, Tyson GW. CheckM: assessing the quality of microbial genomes recovered from isolates, single cells, and metagenomes. Genome Research 2015;25(7):1043–1055. https://doi.org/10.1101/gr.186072.114.
55. Hyatt D, Chen GL, LoCascio PF, Land ML, Larimer FW, Hauser LJ. Prodigal: prokaryotic gene recognition and translation initiation site identification. BMC Bioinformatics 2010;11:1–11. https://doi.org/10.1186/1471-2105-11-119.
56. Sahlin K. Strobealign: flexible seed size enables ultra-fast and accurate read alignment. Genome Biology 2022;23(1):260. https://doi.org/10.1186/s13059-022-02831-7.
57. Kutuzova S, Piera P, Nor Nielsen K, Olsen NS, Riber L, Gobbi A, et al. Binning meets taxonomy: TaxVAMB improves metagenome binning using bi-modal variational autoencoder. bioRxiv 2024;https://doi.org/10.1101/2024.10.25.620172.
58. Rühlemann MC, Wacker EM, Ellinghaus D, Franke A. MAGScoT: a fast, lightweight and accurate bin-refinement tool. Bioinformatics 2022;38(24):5430–5433. https://doi.org/10.1093/bioinformatics/btac694.
59. Eddy SR. Accelerated profile HMM searches. PLOS Computational Biology 2011;7(10):e1002195. https://doi.org/10.1371/journal.pcbi.1002195.
60. Haft DH, Selengut JD, Richter RA, Harkins D, Basu MK, Beck E. TIGRFAMs and genome properties in 2013. Nucleic Acids Research 2012;41(D1):D387–D395. https://doi.org/10.1093/nar/gks1234.
61. Chklovski A, Parks DH, Woodcroft BJ, Tyson GW. CheckM2: a rapid, scalable and accurate tool for assessing microbial genome quality using machine learning. Nature Methods 2023;20(8):1203–1212. https://doi.org/10.1038/s41592-023-01940-w.
62. Ondov BD, Treangen TJ, Melsted P, Mallonee AB, Bergman NH, Koren S, et al. Mash: fast genome and metagenome distance estimation using MinHash. Genome Biology 2016;17:1–14. https://doi.org/10.1186/s13059-016-0997-x.
63. Chaumeil PA, Mussig AJ, Hugenholtz P, Parks DH. GTDB-Tk v2: memory friendly classification with the genome taxonomy database. Bioinformatics 2022;38(23):5315–5316. https://doi.org/10.1093/bioinformatics/btac672.
64. Letunic I, Bork P. Interactive Tree of Life (iTOL) v6: recent updates to the phylogenetic tree display and annotation tool. Nucleic Acids Research 2024;52(W1):W78–W82. https://doi.org/10.1093/nar/gkae268.
65. Bağcı C, Soil Metagenome Pipeline. WorkflowHub; 2025. https://workflowhub.eu/workflows/1960. https://doi.org/10.48546/workflowhub.workflow.1960.1.
66. Blanco-Míguez A, Beghini F, Cumbo F, McIver LJ, Thompson KN, Zolfo M, et al. Extending and improving metagenomic taxonomic profiling with uncharacterized species using MetaPhlAn 4. Nature Biotechnology 2023;41(11):1633–1644. https://doi.org/10.1038/s41587-023-01688-w.
67. Chen J, Jia Y, Sun Y, Liu K, Zhou C, Liu C, et al. Global marine microbial diversity and its potential in bioprospecting. Nature 2024;633(8029):371–379. https://doi.org/10.1038/s41586-024-07891-2.
68. Pasolli E, Asnicar F, Manara S, Zolfo M, Karcher N, Armanini F, et al. Extensive unexplored human microbiome diversity revealed by over 150,000 genomes from metagenomes spanning age, geography, and lifestyle. Cell 2019;176(3):649–662. https://doi.org/10.1016/j.cell.2019.01.001.
69. Sunagawa S, Coelho LP, Chaffron S, Kultima JR, Labadie K, Salazar G, et al. Structure and function of the global ocean microbiome. Science 2015;348(6237):1261359. https://doi.org/10.1126/science.1261359.
70. Navarro-Muñoz JC, Selem-Mojica N, Mullowney MW, Kautsar SA, Tryon JH, Parkinson EI, et al. A computational framework to explore large-scale biosynthetic diversity. Nature Chemical Biology 2020;16(1):60–68. https://doi.org/10.1038/s41589-019-0400-9.
71. Bowers RM, Kyrpides NC, Stepanauskas R, Harmon-Smith M, Doud D, Reddy T, et al. Minimum information about a single amplified genome (MISAG) and a metagenome-assembled genome (MIMAG) of bacteria and archaea. Nature Biotechnology 2017;35(8):725–731. https://doi.org/10.1038/nbt.3893.
72. Kim CY, Lee M, Yang S, Kim K, Yong D, Kim HR, et al. Human reference gut microbiome catalog including newly assembled genomes from under-represented Asian metagenomes. Genome Medicine 2021;13:1–20. https://doi.org/10.1186/s13073-021-00950-7.
73. Ni J, Yan Q, Yu Y. How much metagenomic sequencing is enough to achieve a given goal? Scientific Reports 2013;3(1):1968. https://doi.org/10.1038/srep01968.
74. Duarte CM, Ngugi DK, Alam I, Pearman J, Kamau A, Eguiluz VM, et al. Sequencing effort dictates gene discovery in marine microbial metagenomes. Environmental Microbiology 2020;22(11):4589–4603. https://doi.org/10.1111/1462-2920.15182.
75. Nishimura Y, Yoshizawa S. The OceanDNA MAG catalog contains over 50,000 prokaryotic genomes originated from various marine environments. Scientific Data 2022;9(1):305. https://doi.org/10.1038/s41597-022-01392-5.
76. Sereika M, Mussig AJ, Jiang C, Knudsen KS, Jensen TBN, Petriglieri F, et al. Genome-resolved long-read sequencing expands known microbial diversity across terrestrial habitats. Nature Microbiology 2025;p. 1–13. https://doi.org/10.1038/s41564-025-02062-z.
77. Johansen PL, Chatzigiannidou I, Berzina L, Kristiansen K, Brix S. Unveiling soil microbial diversity through ultra-deep short-read metagenomic sequencing and co-assembly. iMeta 2025;p. e70075. https://doi.org/10.1002/imt2.70075.
78. Burian J, Boer RE, Hernandez Y, Morales-Amador A, Jiang L, Bhattacharjee A, et al. Bioactive molecules unearthed by terabase-scale long-read sequencing of a soil metagenome. Nature Biotechnology 2025;p. 1–8. https://doi.org/10.1038/s41587-025-02810-w.
79. Richardson L, Allen B, Baldi G, Beracochea M, Bileschi ML, Burdett T, et al. MGnify: the microbiome sequence data analysis resource in 2023. Nucleic Acids Research 2023;51(D1):D753–D759. https://doi.org/10.1093/nar/gkac1080.
80. Gurbich TA, Almeida A, Beracochea M, Burdett T, Burgin J, Cochrane G, et al. MGnify genomes: a resource for biome-specific microbial genome catalogues. Journal of Molecular Biology 2023;435(14):168016. https://doi.org/10.1016/j.jmb.2023.168016.
81. Udwary DW, Doering DT, Foster B, Smirnova T, Kautsar SA, Mouncey NJ. The secondary metabolism collaboratory: a database and web discussion portal for secondary metabolite biosynthetic gene clusters. Nucleic Acids Research 2025;53(D1):D717–D723. https://doi.org/10.1093/nar/gkae1060.
82. Larralde M, Zeller G. Machine learning inference of natural product chemistry across biosynthetic gene cluster types. bioRxiv 2025;https://doi.org/10.1101/2025.03.13.642868.
83. Skinnider MA, Johnston CW, Gunabalasingam M, Merwin NJ, Kieliszek AM, MacLellan RJ, et al. Comprehensive prediction of secondary metabolite structure and biologi-

cal activity from microbial genome sequences. Nature Communications 2020;11(1):6058. https://doi.org/10.1038/s41467-020-19986-1.
84. Li Z, Riley WJ, Marschmann GL, Karaoz U, Shirley IA, Wu Q, et al. A framework for integrating genomics, microbial traits, and ecosystem biogeochemistry. Nature Communications 2025;16(1):2186. https://doi.org/10.1038/s41467-025-57386-5.
85. Bağcı C, GitHub: Soil Metagenome Pipeline [Source Code]. GitHub; 2025. https://github.com/ZiemertLab/soil-metagenome-pipeline.

Supplementary Table

Click here to access/download
Supplementary Material
Supplementary Table S1.xlsx

Click here to access/download
Supplementary Material
Supplementary Figures.pdf

Personal Cover
Click here to access/download;Personal Cover;CoverLetter.docx

EBERHARD KARLS
UNIVERSITÄT
TÜBINGEN

Angewandte Naturstoffgenomik

Universität Tübingen · Prof. Dr. Nadine Ziemert ·
Interfakultäres Institut für Mikrobiologie und Infektionsmedizin Tübingen (IMIT)
Angewandte Naturstoffgenomik · Auf der Morgenstelle 24 · 72076 Tübingen

Prof. Dr. Nadine Ziemert

Auf der Morgenstelle 24
72076 Tübingen
Telefon +49 7071 29-78841
Telefax +49 7071 29-5979
Nadine.ziemert @uni-tuebingen.de

Scott Edmunds, PhD
Editor-in-Chief
GigaScience

3. July 2025

Dear Editors,

We are pleased to submit our manuscript entitled "Ultra-deep long-read metagenomics captures diverse taxonomic and biosynthetic potential of soil microbes" for consideration in GigaScience.

This study presents one of the deepest metagenomic sequencing efforts to date on a single soil sample, combining 148 Gbp of Oxford Nanopore long-read data with 122 Gbp of Illumina short reads. Our hybrid assembly strategy enabled the recovery of 837 metagenome-assembled genomes (MAGs), including 466 high- and medium-quality genomes, nearly all of which represent previously uncharacterized taxa.

Using a combination of k-mer, 16S rRNA, and single-copy marker gene analysis, our rarefaction analysis demonstrates that even at this exceptional sequencing depth, soil microbial diversity remains unsaturated. These findings provide a critical empirical benchmark for future studies aiming to quantify microbial complexity in environmental samples. In addition, we identified over 11,000 biosynthetic gene clusters (BGCs), the vast majority of which have no close matches in current reference databases, underscoring the largely untapped biosynthetic potential of soil microbiomes.

We believe this work is well aligned with GigaScience's mission to publish data-rich studies and promote transparency and reproducibility in life science research. The preprint version of this manuscript (https://doi.org/10.1101/2025.05.28.656579) has already attracted significant attention in the community, reflecting strong interest in efforts to quantify microbial and biosynthetic diversity in environmental samples at ultra-deep resolution.

The raw sequencing data and assembled genomes have already been deposited and made publicly available in the European Nucleotide Archive (ENA). If the manuscript is considered, we will make all associated analysis results, including biosynthetic gene cluster annotations and detailed analysis of the MAGs, publicly available via GigaDB.

All authors have approved the manuscript and declare no competing interests. This work is not under consideration elsewhere.

Thank you for considering our submission. We look forward to your feedback.

Sincerely,

Interfakultäres Institut für Mikrobiologie und Infektionsmedizin (IMIT)
Translational Genome Mining · Auf der Morgenstelle 28 · 72076 Tübingen
Telefon +49 7071 29-78840 · Telefax +49 7071 29-5979
Nadine.ziemert@uni-tuebingen.de

Prof. Dr. Nadine Ziemert

Dear Editor,

We thank you and the reviewers for the constructive and insightful feedback, which has substantially improved our manuscript. Below we provide a point-by-point response to each comment. Reviewers' comments are shown in italics, followed by our responses. We would like note that we have also addressed the editorial comment and made our computational workflow available on GitHub and deposited it on workflowhub.eu.

Reviewer #1: Using the deepest soil metagenomic sequencing to date, this study reconstructed 837 MAGs and over 11,000 BGCs. The results show that even with such deep sequencing, only a fraction of the soil microbial diversity was captured, highlighting the contribution of ultra-deep metagenomics to microbial studies and vast unknown microbial diversity and functional potentials. Although the authors have provided insights for future research on soil microbes using high-depth sequencing and a novel hybrid assembly strategy, there are several major concerns I should be point out:

1. A primary concern with the current analysis is the use of all recovered MAGs, regardless of quality. Although 466 of the 837 MAGs met established high- or medium-quality standards (MIMAG criteria), the inclusion of the remaining low-quality MAGs is problematic. Such genomes are highly susceptible to errors, including fragmentation and contamination, which can lead to spurious findings in taxonomic assignments and the assessment of biosynthetic potential. To enhance the rigor and validity of the study, it is recommended to remove the low-quality MAGs before proceeding with further analysis.

All analyses in our original submission were already based on either the full-assembly (taxonomic novelty of the contigs, for example) or restricted to MAGs that met high- or medium-quality MIMAG standards (466 of 837 bins). We have now made this filtering step more explicit in the Methods and Results to avoid any possible confusion. Figure 5 (previously Figure 1) and all associated analyses continue to reflect only these high- and medium-quality MAGs.

2. The study yielded 10.5 GB of co-assembled contigs with an N50 of 77.8 kb. However, the analysis appears to have only predicted BGCs from recovered MAGs. This approach overlooks the vast majority of the high-quality assembly data and likely underestimates the total biosynthetic potential. We recommend a more comprehensive analysis by predicting BGCs directly from the entire co-assembled contigs. This would likely yield a greater and more diverse results, providing a more complete picture of the community's metabolic capabilities.

We apologise for the lack of clarity. We clarify in the revised manuscript that BGC prediction was performed on the entire hybrid co-assembly, not only on MAGs. We have restructured the Results section to make this explicit and avoid confusion.

3. Normally, 16S community analysis is considered the gold standard for studying microbial diversity. Here, the metagenomic results from different sequencing depths could be compared with the 16S diversity analysis results to see what proportion of the diversity can actually be recovered by metagenomic sequencing depth.

We respectfully disagree that amplicon sequencing would be appropriate in this context. 16S rRNA amplicon surveys have well-documented limitations, including primer bias, inability to resolve strain-level diversity, and systematic underestimation of richness in highly complex environments such as soil. Our goal was to use metagenomic data to provide a genome-resolved, quantitative benchmark of diversity; adding a parallel amplicon study would not improve this resolution and might even mislead by suggesting lower diversity than truly exists.

Some minor comments:
4. At least three versions of GTDB database were used?

We now clarify in the Methods which GTDB versions were used by which tools: v214.1 for taxonomic assignments (MMseqs2, Metabuli), v95 for SemiBin2 (pretrained model), and r220 for GTDB-Tk classification. At the time of analysis these were the most recent versions compatible with each tool.

5. In "Rarefaction analysis" part, how many 16S rRNA sequences were extracted, and how many OTUs were clustered?

We now provide these numbers on Figure 3 and provide a supplementary Figure on the size distribution of the extracted 16S rRNA seqeunces. We have also removed the term "OTU" to avoid implying a taxonomic rank, and we frame these as "clustered 16S rRNA gene sequences."

6. The method and criteria to assess novel BGCs comparing against the MIBiG 4.0 and BGC Atlas databases

We added a detailed description of novelty assessment in the Methods, including the BiG-SCAPE and BiG-SLiCE distance thresholds used and cross-checking against MIBiG 4.0 and BGC Atlas.

7. First part of the results section seems to overlap with the methods section

We retained a brief description of key steps in the Results to aid readers who jump directly to this section. This summary is concise and limited to the minimum needed for readability.

8. Figure 1A and 2B can be arranged in one line.

The figure numbering has changed: the content that was previously Figure 1 is now part of Figure 5 (MAG novelty). If Figure 2A and 2B are meant, the relative abundances for taxonomic assignments and novelty are more interpretable when presented separately. We believe that combining them would compromise readability but are happy to follow the journal's typesetting team's recommendation if they prefer to merge.

9. In figure4, the rarefaction lines for 100 and 97 cluster level are totally coincident. It is quite intriguing. This makes me wonder if the Nanopore data was not corrected during the 16S sequence prediction. Nanopore data has a high error rate, and using the raw data directly without correction would lead to significant errors.

We appreciate this comment and have clarified our approach in the revised manuscript. The 97% and 100% clustering curves are indeed very close but not identical. The slight convergence likely reflects a combination of true strain-level diversity and the inherent error rate of Nanopore reads. Error correction is not feasible at this depth because most 16S sequences originate from low-abundance taxa with limited coverage. Nevertheless, we validated the extracted sequences by aligning them to the Illumina-only assembly. There were only 176 full-length 16S rRNA genes detected in the short-read assembly, due to its fragmented nature. Of these 176, 12 match to the Nanopore reads with >99.9% accuracy, 16 with > 99.0% accuracy, and 79 with > 97% accuracy (Data not shown). Considering some might be missing in full length in the Nanopore sequencing, more than half of the 16S rRNAs predicted from Nanopore reads should still have above 97% accuracy.

Reviewer #2: By applying ultra-deep hybrid metagenomic sequencing (270 Gbp total; 148 Gbp Nanopore + 122 Gbp Illumina) to a single Cambisol forest soil sample, the authors achieve near-unprecedented recovery of soil microbial diversity and biosynthetic potential. The work demonstrates the vast, untapped microbial and genetic resource content in soils—particularly in terms of biosynthetic gene clusters (BGCs) and uncultivated microbial lineages. Furthermore, the study underscores the necessity of employing longer and deeper sequencing strategies in order to access the full extent of the soil microbial "dark matter." However, the following points are raised for the authors' consideration:

1. The study is based on a single soil sample, which limits ecological generalizability and the interpretability of the dataset. A brief discussion of how representative this sample might be would help contextualize the findings.

We agree and have added a statement to the Discussion clarifying that this study represents a depth benchmark for a representative Cambisol soil rather than a global census. We note that the heavy-tailed diversity distribution is expected to hold for other complex soils, but taxonomic composition may vary with soil type, season, and land use.

2. The authors state that "Full-length 16S rRNA gene sequences were extracted from Nanopore reads using Barrnap [47] (v0.9)." However, it is unclear how the authors define or validate that the extracted 16S rRNA sequences are indeed "full-length." In typical metagenomic datasets—especially from complex environments like soil—16S rRNA gene sequences are often fragmented due to challenges in assembly, repetitive regions, and read truncation. I recommend the authors provide a length distribution summary of the extracted 16S rRNA gene sequences. This information is critical for assessing the completeness and taxonomic resolution of 16S-based analyses.

We now provide the length distribution of extracted 16S rRNA sequences in the Supplementary data and specify that only sequences flagged as "partial" by Barrnap were excluded.

3. In Figure 2B, the authors present the taxonomic novelty of assembled contigs, with bar plots indicating the proportion of sequences assigned to cultured taxa (blue), uncultured taxa (green), and completely unclassified taxa (orange). However, it appears that the proportion of uncultured taxa—those detected in other metagenomic datasets but lacking cultured representatives—is surprisingly low at certain taxonomic levels (e.g., only ~5% at the species level were uncultured). Given the known high proportion of uncultured lineages in soil microbiomes, this result seems unexpected. Could the authors clarify how these proportions were calculated? Specifically:

** What database and criteria were used to distinguish between "uncultured" vs. "unclassified"?*
** Were contigs with ambiguous or low-confidence taxonomic assignments excluded or grouped into*

"unclassified"?
A more detailed explanation—perhaps in the figure legend or Supplementary Methods—would help readers better interpret the novelty metrics presented in this important figure.

We expanded the Methods and figure legends to explain the database sources (GTDB metadata), the criteria for "cultured," "uncultured," and "unclassified," and how ambiguous assignments were handled.

4. The manuscript states that >99% of BGCs are novel. While this is likely true given soil diversity, it would be helpful to describe the criteria used to define "novelty" (e.g., BiG-SCAPE clustering distance to known MIBiG entries). A summary of the major BGC types (NRPS, PKS, RiPPs, terpenes, etc.) and their taxonomic affiliations would also enhance interpretability.

As noted above, we added the BiG-SCAPE and BiG-SLiCE distance thresholds to the Methods. Figure 4C summarises BGC product types by phylum, providing a clearer view of the taxonomic distribution of biosynthetic capacity.

5. The manuscript's analytical structure raises some concerns. The flow of results appears somewhat fragmented: the authors begin by analyzing MAGs, then shift to contig-based taxonomic novelty, followed by sequencing depth diagnostics (e.g., rarefaction of 16S sequences), and finally return to MAG-based BGC analysis. This sequence may confuse readers and obscure the relationship between the different analytical layers. A clearer explanation of the rationale for this order, or a reorganization to reflect the analytical logic (e.g., assembly → gene catalog → MAGs → functional potential), could improve the manuscript's coherence.

We agree that the flow of the presentation of results in the initial submission was difficult to follow, and have reorganized the Results to follow a more intuitive flow: assembly → taxonomic classification → diversity analyses → functional potential (BGCs) → MAGs. We believe this greatly improves readability.

6. The manuscript would benefit from a more integrated functional perspective. While the BGC analysis is comprehensive, there is little discussion of non-BGC functional annotation—either at the contig level (gene catalog) or the MAG level. Functional profiling using KEGG, eggNOG, or CAZy annotations could provide broader insights into the ecological roles of the recovered genomes and improve the interpretability of the massive sequencing effort.

We performed this analysis and made the data available on zenodo for the total assembly. However, we think discussing this in more detail is beyond the scope of the manuscript. However, this gave us the idea of using coding sequences as another proxy to evaluate the (functional) diversity of the sample. We performed a rarefaction of the functional space and added it to the main manuscript as well.
