# Supplementary material for: Ultra-deep long-read metagenomics captures diverse taxonomic and biosynthetic potential of soil microbes
Source: Gigascience. 2025 Oct 24;14:giaf135. doi: 10.1093/gigascience/giaf135 (PMC12690461; doi:10.1093/gigascience/giaf135)
Supplement: giaf135_Authors_Response_To_Reviewer_Comments_Original_Submission [file giaf135_authors_response_to_reviewer_comments_original_submission.pdf]

Author's Response To Reviewer Comments

Dear Editor,

We thank you and the reviewers for the constructive and insightful feedback, which has substantially improved our manuscript. Below we provide a point-by-point response to each comment. Reviewers' comments are shown in italics, followed by our responses. We would like note that we have also addressed the editorial comment and made our computational workflow available on GitHub and deposited it on workflowhub.eu.

Reviewer #1: Using the deepest soil metagenomic sequencing to date, this study reconstructed 837 MAGs and over 11,000 BGCs. The results show that even with such deep sequencing, only a fraction of the soil microbial diversity was captured, highlighting the contribution of ultra-deep metagenomics to microbial studies and vast unknown microbial diversity and functional potentials. Although the authors have provided insights for future research on soil microbes using high-depth sequencing and a novel hybrid assembly strategy, there are several major concerns I should be point out:

1. A primary concern with the current analysis is the use of all recovered MAGs, regardless of quality. Although 466 of the 837 MAGs met established high- or medium-quality standards (MIMAG criteria), the inclusion of the remaining low-quality MAGs is problematic. Such genomes are highly susceptible to errors, including fragmentation and contamination, which can lead to spurious findings in taxonomic assignments and the assessment of biosynthetic potential. To enhance the rigor and validity of the study, it is recommended to remove the low-quality MAGs before proceeding with further analysis.

All analyses in our original submission were already based on either the full-assembly (taxonomic novelty of the contigs, for example) or restricted to MAGs that met high- or medium-quality MIMAG standards (466 of 837 bins). We have now made this filtering step more explicit in the Methods and Results to avoid any possible confusion. Figure 5 (previously Figure 1) and all associated analyses continue to reflect only these high- and medium-quality MAGs.

2. The study yielded 10.5 GB of co-assembled contigs with an N50 of 77.8 kb. However, the analysis appears to have only predicted BGCs from recovered MAGs. This approach overlooks the vast majority of the high-quality assembly data and likely underestimates the total biosynthetic potential. We recommend a more comprehensive analysis by predicting BGCs directly from the entire co-assembled contigs. This would likely yield a greater and more diverse results, providing a more complete picture of the community's metabolic capabilities.

We apologise for the lack of clarity. We clarify in the revised manuscript that BGC prediction was performed on the entire hybrid co-assembly, not only on MAGs. We have restructured the Results section to make this explicit and avoid confusion.

3. Normally, 16S community analysis is considered the gold standard for studying microbial diversity. Here, the metagenomic results from different sequencing depths could be compared with the 16S diversity analysis results to see what proportion of the diversity can actually be recovered by metagenomic sequencing depth.

We respectfully disagree that amplicon sequencing would be appropriate in this context. 16S rRNA amplicon surveys have well-documented limitations, including primer bias, inability to resolve strain-level diversity, and systematic underestimation of richness in highly complex environments such as soil. Our goal was to use metagenomic data to provide a genome-resolved, quantitative benchmark of diversity; adding a parallel amplicon study would not improve this resolution and might even mislead by suggesting lower diversity than truly exists.

Some minor comments:
4. At least three versions of GTDB database were used?

We now clarify in the Methods which GTDB versions were used by which tools: v214.1 for taxonomic assignments (MMseqs2, Metabuli), v95 for SemiBin2 (pretrained model), and r220 for GTDB-Tk classification. At the time of analysis these were the most recent versions compatible with each tool.

5. In "Rarefaction analysis" part, how many 16S rRNA sequences were extracted, and how many OTUs were clustered?

We now provide these numbers on Figure 3 and provide a supplementary Figure on the size distribution of the extracted 16S rRNA seqeunces. We have also removed the term "OTU" to avoid implying a taxonomic rank, and we frame these as "clustered 16S rRNA gene sequences."

6. The method and criteria to assess novel BGCs comparing against the MIBiG 4.0 and BGC Atlas databases

We added a detailed description of novelty assessment in the Methods, including the BiG-SCAPE and BiG-SLiCE distance thresholds used and cross-checking against MIBiG 4.0 and BGC Atlas.

7. First part of the results section seems to overlap with the methods section

We retained a brief description of key steps in the Results to aid readers who jump directly to this section. This summary is concise and limited to the minimum needed for readability.

8. Figure 1A and 2B can be arranged in one line.

The figure numbering has changed: the content that was previously Figure 1 is now part of Figure 5 (MAG novelty). If Figure 2A and 2B are meant, the relative abundances for taxonomic assignments and novelty are more interpretable when presented separately. We believe that combining them would compromise readability but are happy to follow the journal's typesetting team's recommendation if they prefer to merge.

9. In figure4, the rarefaction lines for 100 and 97 cluster level are totally coincident. It is quite intriguing. This makes me wonder if the Nanopore data was not corrected during the 16S sequence prediction. Nanopore data has a high error rate, and using the raw data directly without correction would lead to significant errors.

We appreciate this comment and have clarified our approach in the revised manuscript. The 97% and 100% clustering curves are indeed very close but not identical. The slight convergence likely reflects a combination of true strain-level diversity and the inherent error rate of Nanopore reads. Error correction is not feasible at this depth because most 16S sequences originate from low-abundance taxa with limited coverage. Nevertheless, we validated the extracted sequences by aligning them to the Illumina-only assembly. There were only 176 full-length 16S rRNA genes detected in the short-read assembly, due to its fragmented nature. Of these 176, 12 match to the Nanopore reads with >99.9% accuracy, 16 with > 99.0% accuracy, and 79 with > 97% accuracy (Data not shown). Considering some might be missing in full length in the Nanopore sequencing, more than half of the 16S rRNAs predicted from Nanopore reads should still have above 97% accuracy.

Reviewer #2: By applying ultra-deep hybrid metagenomic sequencing (270 Gbp total; 148 Gbp Nanopore + 122 Gbp Illumina) to a single Cambisol forest soil sample, the authors achieve near-unprecedented recovery of soil microbial diversity and biosynthetic potential. The work demonstrates the vast, untapped microbial and genetic resource content in soils—particularly in terms of biosynthetic gene clusters (BGCs) and uncultivated microbial lineages. Furthermore, the study underscores the necessity of employing longer and deeper sequencing strategies in order to access the full extent of the soil microbial "dark matter." However, the following points are raised for the authors' consideration:

1. The study is based on a single soil sample, which limits ecological generalizability and the interpretability of the dataset. A brief discussion of how representative this sample might be would help contextualize the

findings.

We agree and have added a statement to the Discussion clarifying that this study represents a depth benchmark for a representative Cambisol soil rather than a global census. We note that the heavy-tailed diversity distribution is expected to hold for other complex soils, but taxonomic composition may vary with soil type, season, and land use.

2. The authors state that "Full-length 16S rRNA gene sequences were extracted from Nanopore reads using Barrnap [47] (v0.9)." However, it is unclear how the authors define or validate that the extracted 16S rRNA sequences are indeed "full-length." In typical metagenomic datasets—especially from complex environments like soil—16S rRNA gene sequences are often fragmented due to challenges in assembly, repetitive regions, and read truncation. I recommend the authors provide a length distribution summary of the extracted 16S rRNA gene sequences. This information is critical for assessing the completeness and taxonomic resolution of 16S-based analyses.

We now provide the length distribution of extracted 16S rRNA sequences in the Supplementary data and specify that only sequences flagged as “partial” by Barrnap were excluded.

3. In Figure 2B, the authors present the taxonomic novelty of assembled contigs, with bar plots indicating the proportion of sequences assigned to cultured taxa (blue), uncultured taxa (green), and completely unclassified taxa (orange). However, it appears that the proportion of uncultured taxa—those detected in other metagenomic datasets but lacking cultured representatives—is surprisingly low at certain taxonomic levels (e.g., only ~5% at the species level were uncultured). Given the known high proportion of uncultured lineages in soil microbiomes, this result seems unexpected. Could the authors clarify how these proportions were calculated? Specifically:

* What database and criteria were used to distinguish between "uncultured" vs. "unclassified"?
* Were contigs with ambiguous or low-confidence taxonomic assignments excluded or grouped into "unclassified"?

A more detailed explanation—perhaps in the figure legend or Supplementary Methods—would help readers better interpret the novelty metrics presented in this important figure.

We expanded the Methods and figure legends to explain the database sources (GTDB metadata), the criteria for “cultured,” “uncultured,” and “unclassified,” and how ambiguous assignments were handled.

4. The manuscript states that >99% of BGCs are novel. While this is likely true given soil diversity, it would be helpful to describe the criteria used to define "novelty" (e.g., BiG-SCAPE clustering distance to known MIBiG entries). A summary of the major BGC types (NRPS, PKS, RiPPs, terpenes, etc.) and their taxonomic affiliations would also enhance interpretability.

As noted above, we added the BiG-SCAPE and BiG-SLiCE distance thresholds to the Methods. Figure 4C summarises BGC product types by phylum, providing a clearer view of the taxonomic distribution of biosynthetic capacity.

5. The manuscript's analytical structure raises some concerns. The flow of results appears somewhat fragmented: the authors begin by analyzing MAGs, then shift to contig-based taxonomic novelty, followed by sequencing depth diagnostics (e.g., rarefaction of 16S sequences), and finally return to MAG-based BGC analysis. This sequence may confuse readers and obscure the relationship between the different analytical layers. A clearer explanation of the rationale for this order, or a reorganization to reflect the analytical logic (e.g., assembly → gene catalog → MAGs → functional potential), could improve the manuscript's coherence.

We agree that the flow of the presentation of results in the initial submission was difficult to follow, and have reorganized the Results to follow a more intuitive flow: assembly → taxonomic classification → diversity analyses → functional potential (BGCs) → MAGs. We believe this greatly improves readability.

6. The manuscript would benefit from a more integrated functional perspective. While the BGC analysis is

comprehensive, there is little discussion of non-BGC functional annotation—either at the contig level (gene catalog) or the MAG level. Functional profiling using KEGG, eggNOG, or CAZy annotations could provide broader insights into the ecological roles of the recovered genomes and improve the interpretability of the massive sequencing effort.

We performed this analysis and made the data available on zenodo for the total assembly. However, we think discussing this in more detail is beyond the scope of the manuscript. However, this gave us the idea of using coding sequences as another proxy to evaluate the (functional) diversity of the sample. We performed a rarefaction of the functional space and added it to the main manuscript as well.

Editorial Manager®

Cookie Preference Center

We use cookies which are necessary to make our site work. We may also use additional cookies to analyze, improve and personalize our content and your digital experience. For more information, see our Cookie Policy.

You may choose not to allow some types of cookies. However, blocking some types may impact your experience of our site and the services we are able to offer. See the different category headings below to find out more or change your settings.

Allow all

Manage Consent Preferences

Strictly Necessary Cookies

Always active

These cookies are necessary for the website to function and cannot be switched off in our systems. They are usually only set in response to actions made by you which amount to a request for services, such as setting your privacy preferences, logging in or filling in forms. You can set your browser to block or alert you about these cookies, but some parts of the site will not then work.

Functional Cookies

These cookies enable the website to provide enhanced functionality and personalisation. They may be set by us or by third party providers whose services we have added to our pages. If you do not allow these cookies then some or all of these services may not function properly.

Performance Cookies

These cookies allow us to count visits and traffic sources so we can measure and improve the performance of our site. They help us to know which pages are the most and least popular and see how visitors move around the site.

Targeting Cookies

These cookies may be set through our site by our advertising partners. They may be used by those companies to build a profile of your interests and show you relevant adverts on other sites. If you do not allow these cookies, you will experience less targeted advertising.

Cookie List

Clear

Apply Cancel
Consent Leg.Interest

Confirm my choices

Powered by onetrus
