# Supplementary material for: Ultra-deep long-read metagenomics captures diverse taxonomic and biosynthetic potential of soil microbes
Source: Gigascience. 2025 Oct 24;14:giaf135. doi: 10.1093/gigascience/giaf135 (PMC12690461; doi:10.1093/gigascience/giaf135)
Supplement: giaf135_Reviewer_1_Report_Original_Submission — Zhi-Feng Zhang -- 8/2/2025 [file giaf135_reviewer_1_report_original_submission.pdf]

**Reviewer Report**

**Title: Ultra-deep long-read metagenomics captures diverse taxonomic and biosynthetic potential of soil microbes**

**Version: Original Submission** **Date:** 8/2/2025

**Reviewer name: Zhi-Feng Zhang**

**Reviewer Comments to Author:**

Using the deepest soil metagenomic sequencing to date, this study reconstructed 837 MAGs and over 11,000 BGCs. The results show that even with such deep sequencing, only a fraction of the soil microbial diversity was captured, highlighting the contribution of ultra-deep metagenomics to microbial studies and vast unknown microbial diversity and functional potentials. Although the authors have provided insights for future research on soil microbes using high-depth sequencing and a novel hybrid assembly strategy, there are several major concerns I should be point out:

1. A primary concern with the current analysis is the use of all recovered MAGs, regardless of quality. Although 466 of the 837 MAGs met established high- or medium-quality standards (MIMAG criteria), the inclusion of the remaining low-quality MAGs is problematic. Such genomes are highly susceptible to errors, including fragmentation and contamination, which can lead to spurious findings in taxonomic assignments and the assessment of biosynthetic potential. To enhance the rigor and validity of the study, it is recommended to remove the low-quality MAGs before proceeding with further analysis.
2. The study yielded 10.5 GB of co-assembled contigs with an N50 of 77.8 kb. However, the analysis appears to have only predicted BGCs from recovered MAGs. This approach overlooks the vast majority of the high-quality assembly data and likely underestimates the total biosynthetic potential. We recommend a more comprehensive analysis by predicting BGCs directly from the entire co-assembled contigs. This would likely yield a greater and more diverse results, providing a more complete picture of the community's metabolic capabilities.
3. Normally, 16S community analysis is considered the gold standard for studying microbial diversity. Here, the metagenomic results from different sequencing depths could be compared with the 16S diversity analysis results to see what proportion of the diversity can actually be recovered by metagenomic sequencing depth.

Some minor comments:

4. At least three versions of GTDB database were used?
5. In "Rarefaction analysis" part, how many 16S rRNA sequences were extracted, and how many OTUs were clustered?
6. The method and criteria to assess novel BGCs comparing against the MIBiG 4.0 and BGC Atlas databases
7. First part of the results section seems to overlap with the methods section
8. Figure 1A and 2B can be arranged in one line.
9. In figure4, the rarefaction lines for 100 and 97 cluster level are totally coincident. It is quite intriguing. This makes me wonder if the Nanopore data was not corrected during the 16S sequence prediction.

Nanopore data has a high error rate, and using the raw data directly without correction would lead to significant errors.

**Methods**

Are the methods appropriate to the aims of the study, are they well described, and are necessary controls included? Choose an item.

**Conclusions**

Are the conclusions adequately supported by the data shown? Choose an item.

**Reporting Standards**

Does the manuscript adhere to the journal's guidelines on minimum standards of reporting? Choose an item.

Choose an item.

**Statistics**

Are you able to assess all statistics in the manuscript, including the appropriateness of statistical tests used? Choose an item.

**Quality of Written English**

Please indicate the quality of language in the manuscript: Choose an item.

**Declaration of Competing Interests**

Please complete a declaration of competing interests, considering the following questions:

- Have you in the past five years received reimbursements, fees, funding, or salary from an organisation that may in any way gain or lose financially from the publication of this manuscript, either now or in the future?
- Do you hold any stocks or shares in an organisation that may in any way gain or lose financially from the publication of this manuscript, either now or in the future?
- Do you hold or are you currently applying for any patents relating to the content of the manuscript?
- Have you received reimbursements, fees, funding, or salary from an organization that holds or has applied for patents relating to the content of the manuscript?
- Do you have any other financial competing interests?
- Do you have any non-financial competing interests in relation to this paper?

If you can answer no to all of the above, write 'I declare that I have no competing interests' below. If your reply is yes to any, please give details below.

I declare that I have no competing interests

I agree to the open peer review policy of the journal. I understand that my name will be included on my report to the authors and, if the manuscript is accepted for publication, my named report including any attachments I upload will be posted on the website along with the authors' responses. I agree for my report to be made available under an Open Access Creative Commons CC-BY license (http://creativecommons.org/licenses/by/4.0/). I understand that any comments which I do not wish to be included in my named report can be included as confidential comments to the editors, which will not be published.

Choose an item.

To further support our reviewers, we have joined with Publons, where you can gain additional credit to further highlight your hard work (see: https://publons.com/journal/530/gigascience). On publication of this paper, your review will be automatically added to Publons, you can then choose whether or not to claim your Publons credit. I understand this statement.

Yes Choose an item.
