# Supplementary material for: Ultra-deep long-read metagenomics captures diverse taxonomic and biosynthetic potential of soil microbes
Source: Gigascience. 2025 Oct 24;14:giaf135. doi: 10.1093/gigascience/giaf135 (PMC12690461; doi:10.1093/gigascience/giaf135)
Supplement: giaf135_Reviewer_2_Report_Original_Submission — Bin Ma -- 8/14/2025 [file giaf135_reviewer_2_report_original_submission.pdf]

Reviewer Report

Title: Ultra-deep long-read metagenomics captures diverse taxonomic and biosynthetic potential of soil microbes

Version: Original Submission **Date:** 8/14/2025

Reviewer name: Bin Ma

Reviewer Comments to Author:

By applying ultra-deep hybrid metagenomic sequencing (270 Gbp total; 148 Gbp Nanopore + 122 Gbp Illumina) to a single Cambisol forest soil sample, the authors achieve near-unprecedented recovery of soil microbial diversity and biosynthetic potential. The work demonstrates the vast, untapped microbial and genetic resource content in soilsâ€"particularly in terms of biosynthetic gene clusters (BGCs) and uncultivated microbial lineages. Furthermore, the study underscores the necessity of employing longer and deeper sequencing strategies in order to access the full extent of the soil microbial "dark matter." However, the following points are raised for the authors' consideration:
1. The study is based on a single soil sample, which limits ecological generalizability and the interpretability of the dataset. A brief discussion of how representative this sample might be would help contextualize the findings.
2. The authors state that "Full-length 16S rRNA gene sequences were extracted from Nanopore reads using Barrnap [47] (v0.9)." However, it is unclear how the authors define or validate that the extracted 16S rRNA sequences are indeed "full-length." In typical metagenomic datasetsâ€"especially from complex environments like soilâ€"16S rRNA gene sequences are often fragmented due to challenges in assembly, repetitive regions, and read truncation. I recommend the authors provide a length distribution summary of the extracted 16S rRNA gene sequences. This information is critical for assessing the completeness and taxonomic resolution of 16S-based analyses.
3. In Figure 2B, the authors present the taxonomic novelty of assembled contigs, with bar plots indicating the proportion of sequences assigned to cultured taxa (blue), uncultured taxa (green), and completely unclassified taxa (orange). However, it appears that the proportion of uncultured taxaâ€"those detected in other metagenomic datasets but lacking cultured representativesâ€"is surprisingly low at certain taxonomic levels (e.g., only ~5% at the species level were uncultured). Given the known high proportion of uncultured lineages in soil microbiomes, this result seems unexpected. Could the authors clarify how these proportions were calculated? Specifically:
* What database and criteria were used to distinguish between "uncultured" vs. "unclassified"?
* Were contigs with ambiguous or low-confidence taxonomic assignments excluded or grouped into "unclassified"?
A more detailed explanationâ€"perhaps in the figure legend or Supplementary Methodsâ€"would help readers better interpret the novelty metrics presented in this important figure.
4. The manuscript states that >99% of BGCs are novel. While this is likely true given soil diversity, it would be helpful to describe the criteria used to define "novelty" (e.g., BiG-SCAPE clustering distance to known MIBiG entries). A summary of the major BGC types (NRPS, PKS, RiPPs, terpenes, etc.) and their

taxonomic affiliations would also enhance interpretability.
5. The manuscript's analytical structure raises some concerns. The flow of results appears somewhat fragmented: the authors begin by analyzing MAGs, then shift to contig-based taxonomic novelty, followed by sequencing depth diagnostics (e.g., rarefaction of 16S sequences), and finally return to MAG-based BGC analysis. This sequence may confuse readers and obscure the relationship between the different analytical layers. A clearer explanation of the rationale for this order, or a reorganization to reflect the analytical logic (e.g., assembly â†’ gene catalog â†’ MAGs â†’ functional potential), could improve the manuscript's coherence.
6. The manuscript would benefit from a more integrated functional perspective. While the BGC analysis is comprehensive, there is little discussion of non-BGC functional annotationâ€"either at the contig level (gene catalog) or the MAG level. Functional profiling using KEGG, eggNOG, or CAZy annotations could provide broader insights into the ecological roles of the recovered genomes and improve the interpretability of the massive sequencing effort.

Methods

Are the methods appropriate to the aims of the study, are they well described, and are necessary controls included? Choose an item.

Conclusions

Are the conclusions adequately supported by the data shown? Choose an item.

Reporting Standards

Does the manuscript adhere to the journal's guidelines on minimum standards of reporting? Choose an item.

Choose an item.

Statistics

Are you able to assess all statistics in the manuscript, including the appropriateness of statistical tests used? Choose an item.

Quality of Written English

Please indicate the quality of language in the manuscript: Choose an item.

Declaration of Competing Interests

Please complete a declaration of competing interests, considering the following questions:

- Have you in the past five years received reimbursements, fees, funding, or salary from an organisation that may in any way gain or lose financially from the publication of this manuscript, either now or in the future?
- Do you hold any stocks or shares in an organisation that may in any way gain or lose financially from the publication of this manuscript, either now or in the future?
- Do you hold or are you currently applying for any patents relating to the content of the manuscript?
- Have you received reimbursements, fees, funding, or salary from an organization that holds or has applied for patents relating to the content of the manuscript?
- Do you have any other financial competing interests?
- Do you have any non-financial competing interests in relation to this paper?

If you can answer no to all of the above, write 'I declare that I have no competing interests' below. If your reply is yes to any, please give details below.

I declare that I have no competing interests

I agree to the open peer review policy of the journal. I understand that my name will be included on my report to the authors and, if the manuscript is accepted for publication, my named report including any attachments I upload will be posted on the website along with the authors' responses. I agree for my report to be made available under an Open Access Creative Commons CC-BY license (http://creativecommons.org/licenses/by/4.0/). I understand that any comments which I do not wish to be included in my named report can be included as confidential comments to the editors, which will not be published.

Choose an item.

To further support our reviewers, we have joined with Publons, where you can gain additional credit to further highlight your hard work (see: https://publons.com/journal/530/gigascience). On publication of this paper, your review will be automatically added to Publons, you can then choose whether or not to claim your Publons credit. I understand this statement.

Yes Choose an item.
